# Supplementary figures and images for: Wide-Scale Analysis of Human Functional Transcription Factor Binding Reveals a Strong Bias towards the Transcription Start Site
Source: PLoS One. 2007 Aug 29;2(8):e807. doi: 10.1371/journal.pone.0000807 (PMC1950076; doi:10.1371/journal.pone.0000807)

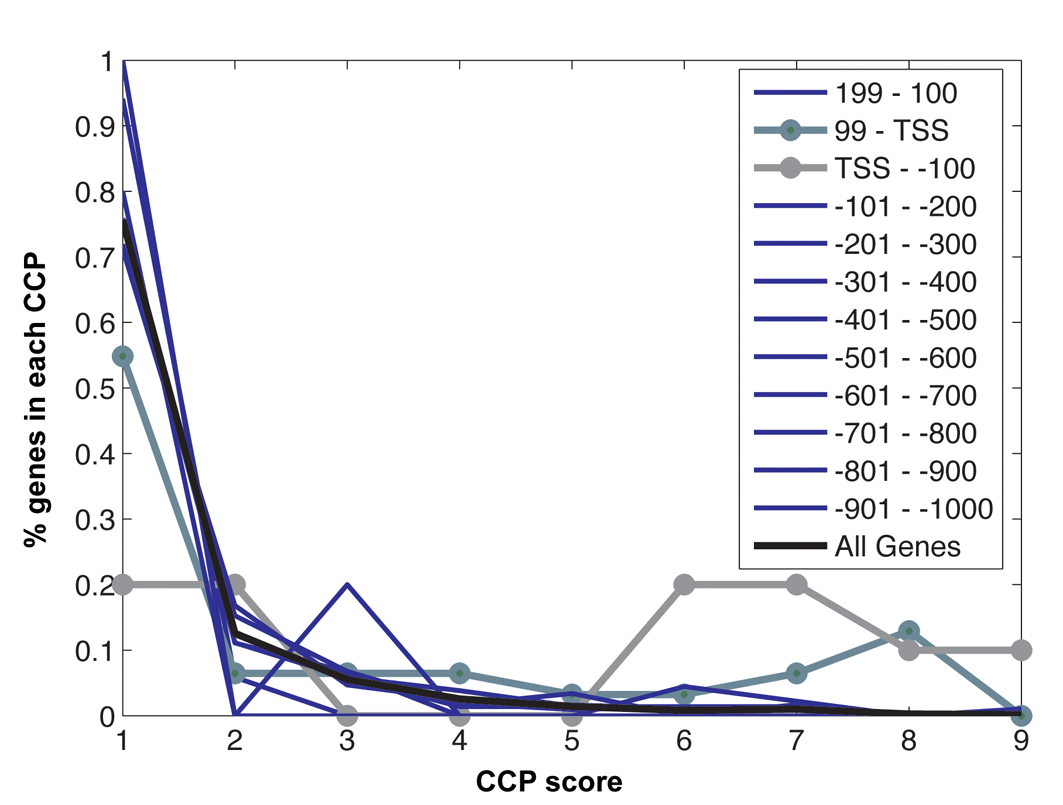

Supplement: Figure S1 — Distributions of the CCP scores of 12 groups of genes that contain the CHR binding motif in their promoters, at 12 different location windows (of 100bp) with respect to the TSS. The black curve is our background: the CCP score distribution of all the genes in the experiment(Whitfield et al. 2002). (2.60 MB TIF) [file pone.0000807.s001.tif]

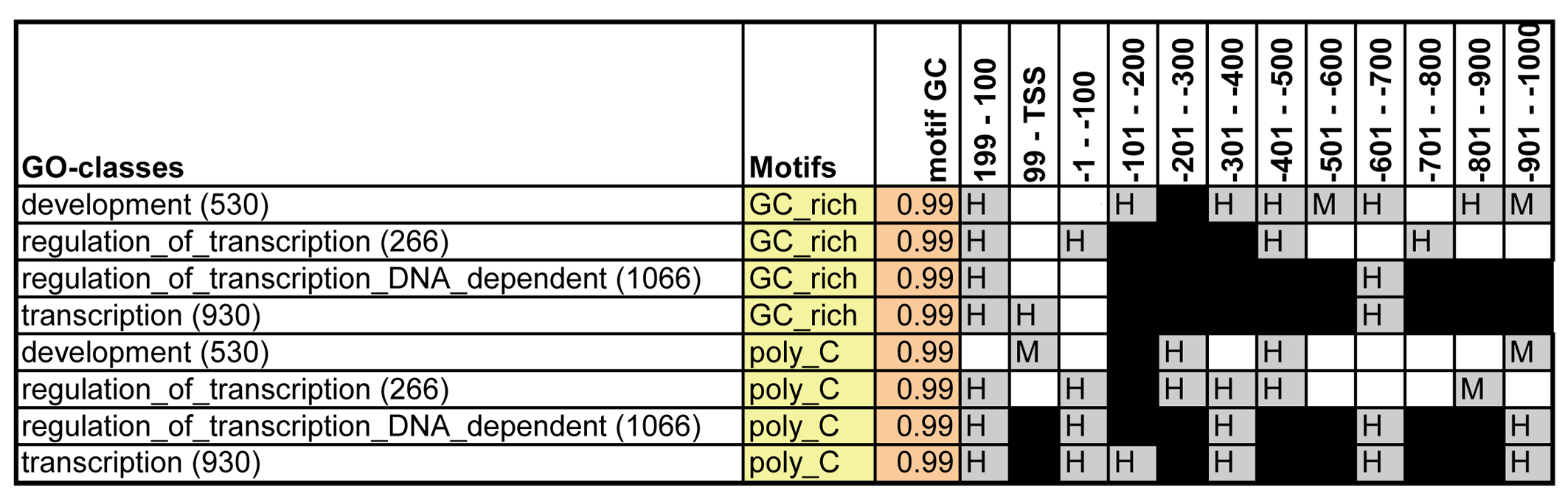

Supplement: Figure S2 — Over representation of GC and C rich “artificial” motifs The hits found in various windows for two “artificial” PSSMs that contain G/C or C with high probability (Table S1) were over represented in human and mouse. MGLC analysis was done for the different windows and for all GO classes (Table S2) as explained in the text. Thirty five MGLCs were found, all of which are associated with one of the 4 GO classes that comprise the transcriptional GO group. (4.23 MB TIF) [file pone.0000807.s002.tif]

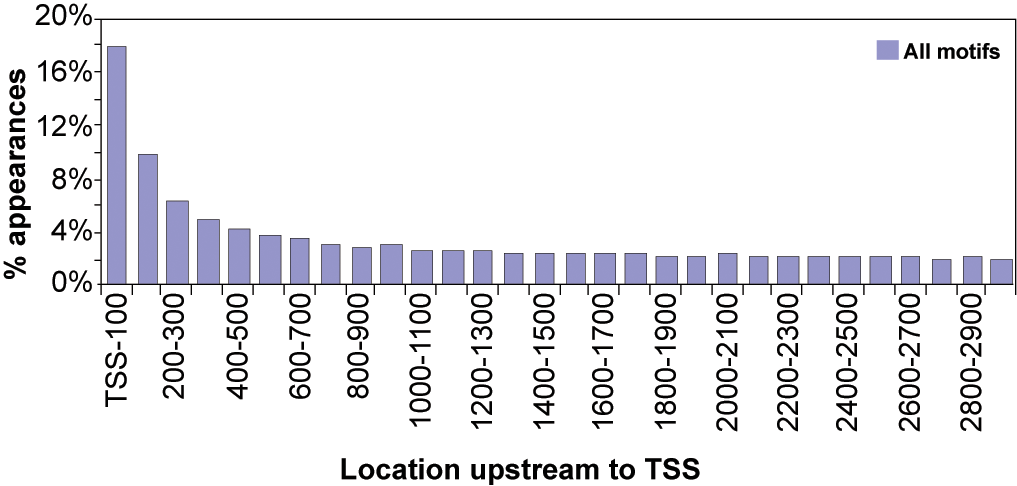

Supplement: Figure S3 — Location distribution of conserved BSs, with high similarity in human, mouse and rat. Location bias analysis of conserved BSs, with high similarity in human, mouse and rat, that were downloaded from the UCSC browser[65] and are based on PSSMs obtained from the Transfac Matrix Database v8.3 created by Biobase [66] . The motifs were aligned according to their distance from the closest TSS and the histogram of these distances was plotted. The total number of conserved binding sites of these motifs is 39829; 11829 (30%) of these were found in the first 200bp, compared to about 4% found in each of the other 200bp long windows between 400bp to 3000bp upstream. (1.51 MB TIF) [file pone.0000807.s003.tif]
