# Supplementary material for: Wide-Scale Analysis of Human Functional Transcription Factor Binding Reveals a Strong Bias towards the Transcription Start Site
Source: PLoS One. 2007 Aug 29;2(8):e807. doi: 10.1371/journal.pone.0000807 (PMC1950076; doi:10.1371/journal.pone.0000807)
Supplement: Table S1 — PSSMs used in our work: TATA PSSM provided by MatIspector (Quandt et al. 1995), and two artificial motifs that were generated by us: poly C motif and GC rich motif. The rows are the different nucleotides; the columns represent the nucleotides' position j in the motif. Each entry mj (n) is the probability to find nucleotide n at position j. (0.01 MB PDF) [file pone.0000807.s004.pdf]

TATA

	1	2	3	4	5	6	7	8	9	10	11
A	0.158	0.043	0.898	0.01	0.903	0.684	0.919	0.567	0.397	0.145	0.214
C	0.372	0.12	0.003	0.028	0.003	0.003	0.01	0.008	0.115	0.346	0.377
G	0.389	0.048	0.008	0.008	0.015	0.003	0.053	0.115	0.402	0.384	0.328
T	0.081	0.789	0.092	0.954	0.079	0.31	0.018	0.31	0.087	0.125	0.081

poly C motif

	1	2	3	4	5	6	7	8	9	10	11	12	13	14	15	16	17	18	19	20	21	22	23	24
A	0.01	0.01	0.01	0.01	0.01	0.01	0.001	0.001	0.001	0.001	0.001	0.001	0.001	0.001	0.001	0.001	0.001	0.001	0.01	0.01	0.01	0.01	0.01	0.01
C	0.97	0.97	0.97	0.97	0.97	0.97	0.997	0.997	0.997	0.997	0.997	0.997	0.997	0.997	0.997	0.997	0.997	0.997	0.97	0.97	0.97	0.97	0.97	0.97
G	0.01	0.01	0.01	0.01	0.01	0.01	0.001	0.001	0.001	0.001	0.001	0.001	0.001	0.001	0.001	0.001	0.001	0.001	0.01	0.01	0.01	0.01	0.01	0.01
T	0.01	0.01	0.01	0.01	0.01	0.01	0.001	0.001	0.001	0.001	0.001	0.001	0.001	0.001	0.001	0.001	0.001	0.001	0.01	0.01	0.01	0.01	0.01	0.01

GC rich motif

	1	2	3	4	5	6	7	8	9	10	11	12	13	14	15	16	17	18	19	20	21	22	23	24
A	0.01	0.01	0.01	0.005	0.005	0.005	0.001	0.001	0.001	0.001	0.001	0.001	0.001	0.001	0.001	0.001	0.001	0.001	0.005	0.005	0.005	0.01	0.01	0.01
C	0.49	0.49	0.49	0.495	0.495	0.495	0.499	0.499	0.499	0.499	0.499	0.499	0.499	0.499	0.499	0.499	0.499	0.499	0.495	0.495	0.495	0.49	0.49	0.49
G	0.49	0.49	0.49	0.495	0.495	0.495	0.499	0.499	0.499	0.499	0.499	0.499	0.499	0.499	0.499	0.499	0.499	0.499	0.495	0.495	0.495	0.49	0.49	0.49
T	0.01	0.01	0.01	0.005	0.005	0.005	0.001	0.001	0.001	0.001	0.001	0.001	0.001	0.001	0.001	0.001	0.001	0.001	0.005	0.005	0.005	0.01	0.01	0.01
