# Supplementary material for: Wide-Scale Analysis of Human Functional Transcription Factor Binding Reveals a Strong Bias towards the Transcription Start Site
Source: PLoS One. 2007 Aug 29;2(8):e807. doi: 10.1371/journal.pone.0000807 (PMC1950076; doi:10.1371/journal.pone.0000807)
Supplement: Table S2 — Full results for over-representation of binding sites. Comprehensive study of the appearances of 414 vertebrate BSs with known PSSMs [34] in the promoters of 8110 human and 23,400 mouse genes. We used groups of human genes that belong to 134 different functional GO classes G, to search for each one of 414 motifs M in twelve 100bp-long windows W in the interval [+200, −1000] bp with respect to the TSS. We examined windows of different sizes (200bp ,300bp, 400bp and 700bp), with and without overlap between the windows. In this paper we focous mainly on the analysis of the 100bp window since its gave us better location specificity and better control on the background GC content. In total, for the 100bp windows, 414×134×12 = 665,712 independent analyses were carried out, one for every (M,G,W) combination. The analysis was carried out for human and mouse, and the statistically significant (M,G,W) combinations are shown. Each row represents an (M,G) pair (first two columns) with a statistically significant over-representation score (passed FDR of 0.10-see text), in at least one location window in Human (grey with the letter H) or Mouse (grey with the letter M) or in both (black box). The BS symbol and its GC content are also given. (0.87 MB PDF) [file pone.0000807.s005.pdf]

100bp Window

GO group	Motifs	motif GC content	199 - 100	99 - TSS	-1 - -100	-101 - -200	-201 - -300	-301 - -400	-401 - -500	-501 - -600	-601 - -700	-701 - -800	-801 - -900	-901 - -1000	GO-classes	Unique motifs
transcription (930)	SP1.01	0.78	H	H	0	H					0			H	transcription GO	1
regulation of transcription DNA dependent (1066)	SP1.01	0.78	H	H	0	H					0				transcription GO	
regulation of transcription (266)	SP1.01	0.78	0	0	0	0	H	0	H	M	0	0	0	0	transcription GO	
transcription (930)	WT1.01	0.77	H	H	H						0	H	H		transcription GO	2
regulation of transcription DNA dependent (1066)	WT1.01	0.77			0						H	H			transcription GO	
development (530)	WT1.01	0.77	0	0	0	H		0	H	H	0	0	0	0	transcription GO	
regulation of transcription (266)	WT1.01	0.77	H	0	H	H		H	H	0	0	H	0	0	transcription GO	
regulation of transcription DNA dependent (1066)	HES1.02	0.76	0	0	0	M	H	0	H	H	0	0	H	0	transcription GO	3
transcription (930)	MAZR.01	0.76	H		H	H					0	H	M	H	transcription GO	4
regulation of transcription DNA dependent (1066)	MAZR.01	0.76	H	H	H	H					M	H		H	transcription GO	
development (530)	MAZR.01	0.76	0	0	0	H	H		H	M	0	H	0	0	transcription GO	
regulation of transcription (266)	MAZR.01	0.76	0	H	0	H	0	0	H	M	0	0	M	0	transcription GO	
transcription (930)	ZNF202.01	0.73	H	H	0	H		H		M	H		0	0	transcription GO	5
regulation of transcription DNA dependent (1066)	ZNF202.01	0.73	H	0	0	H		H		M	H		M	H	transcription GO	
development (530)	ZNF202.01	0.73	0	0	H		H	0	H	0	0	0	0	0	transcription GO	
transcription (930)	AP2.01	0.73	H	0	0	H				H	H	H	H		transcription GO	6
regulation of transcription DNA dependent (1066)	AP2.01	0.73	H	0	0	M				H	H	H	H		transcription GO	
development (530)	AP2.01	0.73	0	0	0	0		M	H	0	H	0	H	0	transcription GO	
regulation of transcription (266)	AP2.01	0.73	0	0	0					0	H	H	H	H	transcription GO	
transcription (930)	ZF5.01	0.72	0	0	0	H				H	H	H	H	H	transcription GO	7
regulation of transcription DNA dependent (1066)	ZF5.01	0.72	H	0	0	H				H	H	H	H		transcription GO	
regulation of transcription (266)	ZF5.01	0.72	0	0	0	H		0	H	0	0	0	0	H	transcription GO	
transcription (930)	ZF9.01	0.72	H	H	0	0	H		H	H	H		M	H	transcription GO	8
regulation of transcription DNA dependent (1066)	ZF9.01	0.72	H	H	0	0	H		H	H				H	transcription GO	
development (530)	ZF9.01	0.72	0	0	0	0	0	M	0	0	0	0	H	0	transcription GO	
regulation of transcription DNA dependent (1066)	EGR3.01	0.71	H	0	0	0	H		H	H	0	0	M	H	transcription GO	9
regulation of transcription (266)	EGR3.01	0.71	0	0	0	0	0	0	0	0	H	0	M	0	transcription GO	
transcription (930)	NRF1_01	0.71	H	H	H	H				H	H	H	H	H	transcription GO	10
regulation of transcription DNA dependent (1066)	NRF1_01	0.71	H	H	H					H	H	H	H	H	transcription GO	
transcription (930)	MYCMAX.03	0.7	0	0	0		H	H	0	H	0	H	0	0	transcription GO	11
regulation of transcription DNA dependent (1066)	MYCMAX.03	0.7	0	0	0		H	0	H	H	0	H	0	0	transcription GO	
transcription (930)	ZBP89.01	0.7	H			0			H	M	0	0	H	0	transcription GO	12
regulation of transcription DNA dependent (1066)	ZBP89.01	0.7	H						H	M	0	H	0	0	transcription GO	
development (530)	ZBP89.01	0.7	0	0	H		H	0	H	M	0	H	M	0	transcription GO	
transcription (930)	NGFIC.01	0.69	H	M	0	0	H	H	H	0	0	0	0	0	transcription GO	13
regulation of transcription DNA dependent (1066)	NGFIC.01	0.69	H	M	0	0	H	H	H	0	H	0	0	0	transcription GO	
transcription (930)	CKROX_01	0.68	H		M	H	H		H		0	H	0	0	transcription GO	14
regulation of transcription DNA dependent (1066)	CKROX_01	0.68	H		M						H	H		0	transcription GO	
development (530)	CKROX_01	0.68	0	0	0	H	H		H	0	0		0	H	transcription GO	
regulation of transcription (266)	CKROX_01	0.68	H	0		H	H	H	H	M	0	H	0	0	transcription GO	
transcription (930)	EGR1.02	0.68	H		H	H		H			H	H	H	H	transcription GO	15
regulation of transcription DNA dependent (1066)	EGR1.02	0.68	H		0							H			transcription GO	
development (530)	EGR1.02	0.68	0	0	0	0	H	H	0	0	0	0	0	M	transcription GO	
regulation of transcription (266)	EGR1.02	0.68	H	0	0			H	H	0	0	0	0	H	transcription GO	
regulation of transcription DNA dependent (1066)	BKLF.01	0.68	0	0	0	0	0	0	H	0	0	0	M	0	transcription GO	16
transcription (930)	GC.01	0.68	0	0	0	0	H	H		M	0	0	0	0	transcription GO	17
regulation of transcription DNA dependent (1066)	GC.01	0.68	H	0	0	0	H	H	H	M	0	0	M	0	transcription GO	
development (530)	MZF1.01	0.68	0	0	H	H	0	H	M	0	H	0	0	0	transcription GO	18
regulation of transcription DNA dependent (1066)	EGR1.01	0.67	H	0	0	H		0	H	M	H	0	H	H	transcription GO	19
regulation of transcription DNA dependent (1066)	MTF-1.01	0.65	0	0	0	H	0		0	M	0	H	0	0	transcription GO	20
transcription (930)	MAZ.01	0.65	H	0	0	H		H			H	H	H	H	transcription GO	21

regulation of transcription DNA dependent (1066)	MAZ.01	0.65	H	H	0	H						H		H	transcription GO	
development (530)	MAZ.01	0.65	0	0	0	H	0	H	H	M	0	H	0	H	transcription GO	
regulation of transcription (266)	MAZ.01	0.65	0	0	0	H	H		H	M	0	H	0	0	transcription GO	
transcription (930)	ZIC2_01	0.65	H	0	0	0	H	M	0	0	0	M	0	0	transcription GO	22
regulation of transcription DNA dependent (1066)	ZIC2_01	0.65	H	0	0	0	H	0	0	0	0	M	0	0	transcription GO	
regulation of transcription DNA dependent (1066)	ATF6.01	0.64	0	0	0	M	0	M	0	H	0	0	H	0	transcription GO	23
transcription (930)	PLAG1_01	0.63	H	0	H	H	0	H		M	H	H	H	H	transcription GO	24
regulation of transcription DNA dependent (1066)	PLAG1_01	0.63	H	M	H	H	H				H		H	H	transcription GO	
development (530)	PLAG1_01	0.63	0	M	H	H	H	H	H	0	H	H	0	0	transcription GO	
regulation of transcription (266)	PLAG1_01	0.63	0	H	H	H	0	0	0	M	0	0	0	H	transcription GO	
transcription (930)	AHRARNT.02	0.62	0	0	0	0	0	H	0	0	0	0	0	M	transcription GO	25
regulation of transcription DNA dependent (1066)	AHRARNT.02	0.62	0	0	0	0	0	H	0	0	H	0	0	M	transcription GO	
regulation of transcription DNA dependent (1066)	HES1.01	0.62	0	0	0	M	H	H		0	0	H		0	transcription GO	26
regulation of transcription DNA dependent (1066)	MUSCLE_INI.02	0.62	0	0	0	H		H	0	H	0	0	0	0	transcription GO	27
regulation of transcription DNA dependent (1066)	MUSCLE_INI.01	0.61	0	0	0	H	H	M	0	H	0	0	0	0	transcription GO	28
regulation of transcription DNA dependent (1066)	ZNF76_143_01	0.6	0	0	0	0	0	0	M	H	H	0	0	0	transcription GO	29
transcription (930)	CDE.01	0.6	M	H	H	H	H		H		H	0	H	H	transcription GO	30
regulation of transcription DNA dependent (1066)	CDE.01	0.6	0	H	0	H			H				H	H	transcription GO	
regulation of transcription DNA dependent (1066)	ZBRK1_01	0.59	0	0	0	0	0	M	0	0	0	0	H	0	transcription GO	31
transcription (930)	E2F.02	0.59	0	H	H	H	0	0	H	H		0	0		transcription GO	32
regulation of transcription DNA dependent (1066)	E2F.02	0.59	0	0	0	H	0	0	H	H		0	0		transcription GO	
transcription (930)	TAXCREB.01	0.58	0	0	0	0	0	H	M	0	0	M	0	0	transcription GO	33
development (530)	PAX5.01	0.57	0	0	0	0	0	0	0	H	0	M	0	0	transcription GO	34
regulation of transcription DNA dependent (1066)	GAGA.01	0.56		0	H	0	H	H	H	M	0	0	M	0	transcription GO	35
development (530)	GAGA.01	0.56	0	0	H	H	H		H	0	0	0	0	0	transcription GO	
transcription (930)	E2F.03	0.54	0	H	H	H	0	H	0	0	M	0	0	H	transcription GO	36
regulation of transcription DNA dependent (1066)	E2F.03	0.54	0	H	H	H	0	H	0	0	M	0	0	H	transcription GO	
development (530)	GKLF.01	0.49	0		0	0	H	H	0	0	0	0	0	0	transcription GO	37
regulation of transcription DNA dependent (1066)	CAAT.01	0.49	0	0	H	H		0	0	0	0	0	0	0	transcription GO	38
development (530)	MTATA.01	0.47	0	0		0	0	0	0	0	0	0	0	0	transcription GO	39
protein amino acid phosphorylation (373)	SP1.01	0.78	0	0	H	0	0	H	M	H	0	H	0	0	general GO	
transcription RNA polyII promoter (190)	SP1.01	0.78	0	0	0	0	0	H	0	0	0	0	M	0	general GO	
small GTPase mediated signal transduction (124)	SP1.01	0.78	H		H	H	H	H	H	0	0	0	0	0	general GO	
intracellular protein transport (191)	SP1.01	0.78	H	M	H	H	0	0	0	0	0	0	0	0	general GO	
regulation of transcription from RNA polymerase II	SP1.01	0.78		H	0	0	0	0	0	0	0	0	0	0	general GO	
transcription RNA polyII promoter (190)	WT1.01	0.77	0	0	0	H	M	H	0	0	0	0	0	0	general GO	
regulation of transcription from RNA polymerase II	MAZR.01	0.76	M	0	H	H	0	H	0	M	0	0	0	0	general GO	
Wnt receptor signaling pathway (66)	AP2.01	0.73	H	0	0	H	H	0	0	0	M	0	0	0	general GO	
small GTPase mediated signal transduction (124)	ZF5.01	0.72	0	M	H	H	0	0	0	0	0	0	0	0	general GO	
transcription RNA polyII promoter (190)	NRF1_01	0.71	0	0	0	0	0		0	0	0	0	0	0	general GO	
intracellular protein transport (191)	NRF1_01	0.71	0	0		H	0	0	0	0	0	0	0	0	general GO	
nervous system development (225)	ZBP89.01	0.7	0	0	H	H	0	0	0	0	0	0	M	0	general GO	
potassium ion transport (108)	ZBP89.01	0.7	0	0	0	0	0	0	M	H	0	0	0	0	general GO	
regulation of transcription from RNA polymerase II	CKROX_01	0.68	M	H	0	M	0	H	0	0	0	0	0	0	general GO	
nervous system development (225)	CKROX_01	0.68	0	0	H	H	0	0	H	0	0	0		0	general GO	
small GTPase mediated signal transduction (124)	EGR1.02	0.68	H			H	H	0	H	0	0	0	0	0	general GO	
Wnt receptor signaling pathway (66)	EGR1.02	0.68	0	M	0	H	0	0	0	H	0	0	0	0	general GO	
protein amino acid phosphorylation (373)	GC.01	0.68	0	0	0	0	0	0		0	0	0	0	0	general GO	
intracellular protein transport (191)	GC.01	0.68	0	M	H	0	0	0	0	0	0	0	0	0	general GO	
intracellular protein transport (191)	EGR1.01	0.67	H	M	H	0	0	0	0	0	0	0	0	0	general GO	
chromatin modification (53)	HES1.01	0.62	0	0	0	0	0	0	0	M	0	H	0	0	general GO	
protein biosynthesis (197)	GABP.01	0.62	0	H		0	0	0	0	0	0	0	0	0	general GO	40
cation transport (99)	NRSF.01	0.62	0	0	M	0	0	0	0	0	0	0	H	0	general GO	41
inflammatory response (163)	NFKAPPAB.01	0.6	0	0		M	0	0	0	0	0	0	0	0	general GO	42
immune response (366)	NFKAPPAB.01	0.6	0	0			0	0	0	0	0	0	0	0	general GO	
intracellular protein transport (191)	ATF6.02	0.6	0		H	0	0	0	0	0	0	0	0	0	general GO	43
transcription RNA polyII promoter (190)	CDE.01	0.6	0	0	0	0	0	0	0	0	0		0	0	general GO	
response to DNA damage stimulus (117)	E2F.02	0.59	0		H	0	0	0	0	0	0	0	0	0	general GO	
nuclear mRNA splicing via spliceosome (94)	NRF2.01	0.57	0	0	H	0		0	0	0	0	0	0	0	general GO	44
protein biosynthesis (197)	NRF2.01	0.57	0	H		0	0	0	0	0	0	0	0	0	general GO	
protein targeting (37)	NRF2.01	0.57	0	0		0	0	0	0	0	0	0	0	0	general GO	
cation transport (99)	NRSE.01	0.57	0	0	M	0	0	0	0	0	0	0	H	0	general GO	45
signal transduction (1189)	NFKAPPAB.02	0.57	0	0		0	0	0	0	0	0	0	0	0	general GO	46
chemotaxis (98)	NFKAPPAB.02	0.57	0	0	H	M	0	0	0	0	0	0	0	0	general GO	

inflammatory response (163)	NFKAPPAB.02	0.57	0	0	H		0	0	0	0	0	0	0	0	general GO	
immune response (366)	NFKAPPAB.02	0.57	0	0			0	0	H	0	0	0	0	0	general GO	
chemotaxis (98)	NFKAPPAB65.01	0.55	0	0	H	M	0	0	0	0	0	0	0	0	general GO	47
inflammatory response (163)	NFKAPPAB65.01	0.55	0	0			0	0	0	0	0	0	0	0	general GO	
immune response (366)	NFKAPPAB65.01	0.55	0	0			0	0	H	0	0	0	0	0	general GO	
cell cell signaling (268)	NFKAPPAB65.01	0.55	0	0	H	M	0	0	0	0	0	0	0	0	general GO	
protein biosynthesis (197)	CETS1P54.01	0.55	0	H		0	0	0	0	0	0	0	0	0	general GO	48
protein biosynthesis (197)	YY1.01	0.55	0		0	0	0	0	0	0	0	0	0	0	general GO	49
chemotaxis (98)	CREL.01	0.55	0	0	H	M	0	0	0	0	0	0	0	0	general GO	50
inflammatory response (163)	CREL.01	0.55	0	0		M	0	0	0	0	0	0	0	0	general GO	
immune response (366)	CREL.01	0.55	0	0		0	0	0	H	0	0	0	0	0	general GO	
protein biosynthesis (197)	ELK1.02	0.55	0	H		0	0	0	0	0	0	0	0	0	general GO	51
signal transduction (1189)	NFKAPPAB.03	0.55	0	0		0	0	0	0	0	0	0	0	0	general GO	52
inflammatory response (163)	NFKAPPAB.03	0.55	0	0			0	0	0	0	0	0	0	0	general GO	
immune response (366)	NFKAPPAB.03	0.55	0	0			0	0	0	0	0	0	0	0	general GO	
response to virus (55)	NFKAPPAB.03	0.55	0	0	H	M	0	0	0	0	0	0	0	0	general GO	
inflammatory response (163)	MYCMAX.02	0.54	H	0	0	0	0	M	0	0	0	0	0	0	general GO	53
protein biosynthesis (197)	ELF2.01	0.54	0	H	M	0	0	0	0	0	0	0	0	0	general GO	54
protein biosynthesis (197)	FLI.01	0.53	0	H		0	0	0	0	0	0	0	0	0	general GO	55
DNA repair (148)	FLI.01	0.53	0	0	M	H	0	0	0	0	0	0	0	0	general GO	
response to DNA damage stimulus (117)	FLI.01	0.53	H	0	M	0	0	0	0	0	0	0	0	0	general GO	
protein biosynthesis (197)	STAF.01	0.53	0	0		0	0	0	0	0	0	0	0	0	general GO	56
protein biosynthesis (197)	STAT1.01	0.52	0	0		0	0	0	0	0	0	0	0	0	general GO	57
signal transduction (1189)	HIVEP1_01	0.52	0	0		0	0	0	0	0	0	0	0	0	general GO	58
chemotaxis (98)	HIVEP1_01	0.52	0	0	H		0	0	0	0	0	0	0	0	general GO	
inflammatory response (163)	HIVEP1_01	0.52	0	0	H		0	0	0	0	0	0	0	0	general GO	
immune response (366)	HIVEP1_01	0.52	0	0		H	0	0	0	0	0	0	0	0	general GO	
sensory perception (201)	VMAF.01	0.52	0	0	H	M	0	0	0	0	0	0	0	0	general GO	59
mRNA processing (148)	NFY.01	0.52	0	0	0	H	0	M	0	0	0	0	0	0	general GO	60
cell cycle (273)	NFY.01	0.52	0	0	H		H	0	0	0	0	0	0	0	general GO	
cell division (103)	NFY.01	0.52	0	0	H	M	H	0	0	0	0	0	0	0	general GO	
mitosis (86)	NFY.01	0.52	0	0		M	H	0	0	0	0	0	0	0	general GO	
nucleosome assembly (71)	NFY.01	0.52	0	0		H	H	0	0	0	0	0	0	0	general GO	
steroid biosynthesis (42)	NFY.01	0.52	0	0	M	H	0	0	0	0	0	0	0	0	general GO	
regulation of cyclin dependent protein kinase activity	NFY.01	0.52	0	0	0	0	H	0	M	0	0	0	0	0	general GO	
inflammatory response (163)	ETS1.01	0.52	0	0	0		0	0	0	0	0	0	0	0	general GO	61
immune response (366)	ETS1.01	0.52	0	0		H	0	0	0	0	0	0	0	0	general GO	
cellular defense response (68)	ETS1.01	0.52	H	0		0	0	0	0	0	0	0	0	0	general GO	
protein biosynthesis (197)	ELK1.01	0.52	0	H		0	0	0	0	0	0	0	0	0	general GO	62
immune response (366)	AML3.01	0.5	0	H			0	0	0	H	0	0	0	0	general GO	63
DNA metabolism (47)	E2F.01	0.5	0		0	0	0	0	0	0	0	0	0	0	general GO	64
immune response (366)	SRF.02	0.49	0	0	H	0	M	0	0	0	0	0	0	0	general GO	65
muscle development (107)	SRF.02	0.49	0	0	H		H	0	0	0	0	0	0	0	general GO	
cell cycle (273)	CAAT.01	0.49	0	0	H	M	H	0	0	0	0	0	0	0	general GO	
mitosis (86)	CAAT.01	0.49	0	0		0	0	0	0	0	0	0	0	0	general GO	
nucleosome assembly (71)	CAAT.01	0.49	0	0	H		H	0	0	0	0	0	0	0	general GO	
lipid biosynthesis (54)	CAAT.01	0.49	0	0	M	H	0	0	0	0	0	0	0	0	general GO	
steroid biosynthesis (42)	CAAT.01	0.49	0	0	M	H	0	0	0	0	0	0	0	0	general GO	
intracellular signaling cascade (257)	TAL1BETAE47.01	0.49	0	0	0	0	0	H	0	0	M	0	0	0	general GO	66
pregnancy (41)	AREB6.01	0.49	H	M	0	0	0	0	0	0	0	0	0	0	general GO	67
immune response (366)	PU1.01	0.48	0	0		0	0	0	0	0	0	0	0	0	general GO	68
inflammatory response (163)	ETS2.01	0.48	0	H	H		0	0	0	0	0	0	0	0	general GO	69
immune response (366)	ETS2.01	0.48	0	H		H	0	0	0	0	0	0	0	0	general GO	
cell cycle (273)	NFY.02	0.48	0	0	H	M	0	0	0	0	0	0	0	0	general GO	70
cell division (103)	NFY.02	0.48	0	0	H	M	H	0	0	0	0	0	0	0	general GO	
mitosis (86)	NFY.02	0.48	0	0		M	0	0	0	0	0	0	0	0	general GO	
chemotaxis (98)	NFE2L2.01	0.47	0	H	0	M	0	0	0	0	0	0	0	0	general GO	71
immune response (366)	NFE2L2.01	0.47	0	0		M	0	H	0	0	0	0	0	0	general GO	
cell cell signaling (268)	MTATA.01	0.47	0	0		0	0	0	0	0	0	0	0	0	general GO	
sensory perception of smell (40)	TAL1BETAITF2.01	0.47	H	0	0	M	0	0	0	0	0	0	0	0	general GO	72
mRNA processing (148)	NFY.03	0.47	0	0	H	0	0	M	0	0	0	0	0	0	general GO	73
cell cycle (273)	NFY.03	0.47	0	0	H	M	0	0	0	0	0	0	0	0	general GO	
cell division (103)	NFY.03	0.47	0	0	H		0	0	0	0	0	0	0	0	general GO	
mitosis (86)	NFY.03	0.47	0	0	H	M	0	0	0	0	0	0	0	0	general GO	

nucleosome assembly (71)	NFY.03	0.47	0	0	H		H	0	0	0	0	0	0	0	general GO	
steroid biosynthesis (42)	NFY.03	0.47	0	0	M	H	0	0	0	0	0	0	0	0	general GO	
immune response (366)	SRF.03	0.47	H	0	0	0	M	0	0	0	0	0	0	0	general GO	74
muscle development (107)	SRF.03	0.47	0	0	H		H	0	0	0	0	0	0	0	general GO	
defense response (100)	ARE.01	0.47	0	M	H	0	0	0	0	0	0	0	0	0	general GO	75
sensory perception (201)	GKLF_02	0.46	0	0		0	0	0	0	0	0	0	0	0	general GO	76
immune response (366)	STAT6.01	0.46	0	H	0		0	H	0	0	0	0	0	0	general GO	77
immune response (366)	MIT.01	0.46	H	0	0	H	M	0	0	0	0	0	0	0	general GO	78
inflammatory response (163)	CEBP.02	0.46	M	0	0	H	0	0	0	0	0	0	0	0	general GO	79
chemotaxis (98)	PXRCAR.01	0.45	0	M	H	0	0	0	0	0	0	0	0	0	general GO	80
immune response (366)	PXRCAR.01	0.45	0	0	H	M	0	0	0	0	0	0	0	0	general GO	
sensory perception (201)	PTX1.01	0.45	0	0	M	H	0	0	0	0	0	0	0	0	general GO	81
visual perception (142)	PTX1.01	0.45	0	0		0	0	H	0	0	0	0	0	0	general GO	
immune response (366)	GATA1.01	0.45	M	0	H	0	0	0	0	0	0	H	0	0	general GO	82
immune response (366)	RTR.01	0.45	0	M	H	H	0	0	0	H	0	0	0	0	general GO	83
inflammatory response (163)	MYT1L.01	0.44	H	M	0	0	0	0	0	0	0	0	0	0	general GO	84
immune response (366)	MYT1L.01	0.44	0	0	H	M	0	0	0	0	0	0	0	0	general GO	
ubiquitin cycle (154)	VMYB.05	0.44	0	0	0	0	M	0	H	0	0	0	0	0	general GO	85
immune response (366)	BCL6.02	0.44	0	0	0		0	0	0	0	0	0	0	0	general GO	86
inflammatory response (163)	TCF11MAFG.01	0.43	0		H	0	0	0	0	0	0	0	0	0	general GO	87
G protein coupled receptor protein signaling pathway	TCF11MAFG.01	0.43	0	0	H	M	0	0	0	0	0	0	0	0	general GO	
immune response (366)	AP1.01	0.43	0	0		0	0	H	0	0	0	0	0	0	general GO	88
inflammatory response (163)	ISRE.01	0.43	0	0	H	M	0	0	0	0	0	0	0	0	general GO	89
immune response (366)	ISRE.01	0.43	0				0	0	0	0	0	0	0	0	general GO	
response to virus (55)	ISRE.01	0.43	0	H	H		0	H	0	0	0	0	0	0	general GO	
immune response (366)	IRF2.01	0.43	0				0	0	0	0	0	0	0	0	general GO	90
response to virus (55)	IRF2.01	0.43	0	0		H	0	H	0	0	0	0	0	0	general GO	
innate immune response (46)	IRF2.01	0.43	0	M	H	0	0	0	0	0	0	0	0	0	general GO	
sensory perception (201)	GSC.01	0.43	0	0		H	0	0	0	0	0	0	0	0	general GO	91
visual perception (142)	GSC.01	0.43	0	0		0	0	0	0	0	0	0	0	0	general GO	
inflammatory response (163)	IRF3.01	0.42	0	0	H	M	0	0	0	0	0	0	0	0	general GO	92
immune response (366)	IRF3.01	0.42	H	0			0	0	0	0	0	0	0	0	general GO	
response to virus (55)	IRF3.01	0.42	H	0	H		0	0	0	0	0	0	0	0	general GO	
sensory perception (201)	SRF.01	0.42	H	0		H	0	0	0	0	0	0	0	0	general GO	93
inflammatory response (163)	SRF.01	0.42	0	0		0	0	0	0	0	0	0	0	0	general GO	
immune response (366)	SRF.01	0.42	H	0	H	0	M	0	0	0	0	0	0	0	general GO	
muscle development (107)	SRF.01	0.42	H	0	H		0	0	0	0	0	0	0	0	general GO	
homophilic cell adhesion (70)	GSH1.01	0.42	0	0	0	0	0	H	0	0	M	0	0	0	general GO	94
immune response (366)	PRDM1.01	0.41	0	0		H	0	0	0	0	0	0	0	0	general GO	95
response to virus (55)	PRDM1.01	0.41	0	0	0	0	0	H	0	0	M	0	0	0	general GO	
immune response (366)	HMGIY.01	0.41	0	0			0	0	0	0	H	0	0	0	general GO	96
cell matrix adhesion (56)	HBP1_01	0.41	0	0	0	0	M	0	H	0	H	0	0	0	general GO	97
immune response (366)	RORA1.01	0.41	0	M	0	0	H	0	0	H	0	0	0	0	general GO	98
sensory perception (201)	GFI1.01	0.41	0	0		0	0	0	0	0	0	0	0	0	general GO	99
immune response (366)	GFI1.01	0.41	M	0	0	H	0	H	0	0	0	0	0	0	general GO	
cell cell signaling (268)	PHOX2_01	0.41	M	0	H	0	0	0	0	0	0	0	0	0	general GO	100
immune response (366)	PSE_02	0.41	0	0	0		0	0	0	0	0	0	0	0	general GO	101
cell division (103)	CHR.01	0.41	0	H		0	0	0	0	0	0	0	0	0	general GO	102
mitosis (86)	CHR.01	0.41	0	H		0	0	0	0	0	0	0	0	0	general GO	
immune response (366)	MTBF.01	0.4		0	H	0	0	0	H	0	0	0	0	0	general GO	103
immune response (366)	IRF1.01	0.4	H				0	0	0	0	0	0	0	0	general GO	104
response to virus (55)	IRF1.01	0.4	0	H		H	0	H	0	0	H	0	0	0	general GO	
defense response (100)	IRF1.01	0.4	0	0		H	0	H	0	0	0	0	0	0	general GO	
inflammatory response (163)	CEBPB.01	0.4	0	0	H		0	H	0	0	0	0	0	0	general GO	105
sensory perception (201)	CRX.01	0.39	0	0		0	H	0	0	0	0	0	0	0	general GO	106
visual perception (142)	CRX.01	0.39	0	0		0	0	0	0	0	0	0	0	0	general GO	
inflammatory response (163)	AARE.01	0.39	0	0		0	0	0	0	0	0	0	0	0	general GO	107
immune response (366)	AARE.01	0.39	0	0		H	H	0	0	0	0	0	0	0	general GO	
proteolysis (297)	AIRE.01	0.39	0	0	0	0	M	H	0	0	0	0	H	0	general GO	108
cell cell signaling (268)	LTATA_01	0.39	0	0		0	0	0	0	0	0	0	0	0	general GO	109
epidermis development (59)	LTATA_01	0.39	0	0		0	0	0	0	0	0	0	0	0	general GO	
inflammatory response (163)	BRN2.01	0.39	0	0	H		0	0	0	0	0	0	0	0	general GO	110
inflammatory response (163)	IRF4.01	0.39	0	0	0	M	H	0	0	0	0	0	0	0	general GO	111
immune response (366)	IRF4.01	0.39	H			H	0	0	0	0	0	0	0	0	general GO	

G protein coupled receptor protein signaling pathway	IRF4.01	0.39	M	0	H	0	0	0	0	0	0	0	0	0	general GO	
visual perception (142)	OTX2.01	0.39	0	0		0	0	0	0	0	0	0	0	0	general GO	112
protein biosynthesis (197)	OTX2.01	0.39	0	0	0	0	0	M	0	0	H	H	0	0	general GO	
immune response (366)	STAT5.01	0.39	H	H	H		H	0	0	0	0	0	0	0	general GO	113
immune response (366)	BCL6.01	0.39	0	H	H		H	M	0	0	0	0	0	0	general GO	114
immune response (366)	OCT1.04	0.38			H	H	0	0	0	0	0	0	0	0	general GO	115
immune response (366)	GFI1B.01	0.38	0	0	0		0	0	0	0	0	0	0	0	general GO	116
RNA processing (58)	GFI1B.01	0.38	M	0	0	0	0	0	0	0	0	0	0	H	general GO	
immune response (366)	AMEF2.01	0.38	0	0	H		0	0	0	0	0	0	0	0	general GO	117
inflammatory response (163)	NFAT.01	0.38	0	0	M		H	0	0	0	0	0	0	0	general GO	118
immune response (366)	NFAT.01	0.38	M	H		H	0	0	0	0	0	0	0	0	general GO	
inflammatory response (163)	CDX2.01	0.38	0	0	H		0	0	0	H	0	0	0	0	general GO	119
immune response (366)	CDX2.01	0.38	M	0		H	H	0	0	0	0	0	0	0	general GO	
inflammatory response (163)	MMEF2.01	0.37	0	0		H	0	0	0	0	0	0	0	0	general GO	120
immune response (366)	GATA1.05	0.36		H	0	0	0	0	0	0	0	0	0	0	general GO	121
sensory perception (201)	XFD3.01	0.36	0	H		0	0	0	0	0	0	0	0	0	general GO	122
inflammatory response (163)	IRF7.01	0.36	M	0	H	0	0	0	0	0	0	0	0	0	general GO	123
immune response (366)	IRF7.01	0.36	H	H			0	0	0	0	0	0	0	0	general GO	
sensory perception (201)	OCT1P.01	0.36	0	0	H		H	H	H	0	0	0	0	0	general GO	124
homophilic cell adhesion (70)	OCT1P.01	0.36	0	0	0	M	0	0	0	0	H	0	0	0	general GO	
immune response (366)	OCT.01	0.35	H			0	0	0	H	0	0	0	0	0	general GO	125
sensory perception (201)	SIX3.01	0.35	0	0		H	H	0	0	0	0	0	0	0	general GO	126
visual perception (142)	SIX3.01	0.35	0	0		0	H	H	0	0	0	0	0	H	general GO	
immune response (366)	PDX1_G_SAAB3	0.35	M	0	0	H	0	0	0	0	0	0	0	0	general GO	127
inflammatory response (163)	CDX1.01	0.35	M	0		0	0	0	0	0	0	0	0	0	general GO	128
immune response (366)	CDX1.01	0.35	H	0		H	0	0	0	0	0	0	0	0	general GO	
immune response (366)	NKX25.02	0.35	M	0	0	H	0	H	0	0	0	0	0	0	general GO	129
sensory perception (201)	FREAC2.01	0.35	0	0	0	0	0	M	0	0	H	0	0	0	general GO	130
immune response (366)	OCT1.02	0.34	H	M	0	0	0	0	H	0	0	0	0	0	general GO	131
inflammatory response (163)	XFD1.01	0.34	0	0	M	H	H	0	0	0	0	0	0	0	general GO	132
immune response (366)	XFD1.01	0.34	H	0	M	H	0	0	0	0	0	0	0	0	general GO	
immune response (366)	GSH2_01	0.34	M	0	0	0	0	0	H	0	0	0	0	0	general GO	133
signal transduction (1189)	EVI1.02	0.33	0	0		0	0	0	0	0	0	0	0	0	general GO	134
immune response (366)	EVI1.02	0.33	0	M	H	0	H	0	0	0	0	0	0	0	general GO	
G protein coupled receptor protein signaling pathway	EVI1.02	0.33	0	0		0	0	0	0	0	0	0	0	0	general GO	
inflammatory response (163)	XFD2.01	0.32	0	0	M	0	0	0	0	0	H	0	0	0	general GO	135
immune response (366)	XFD2.01	0.32	H	0		H	H	0	0	0	H	0	0	0	general GO	
innate immune response (46)	LMX1B.01	0.32	0	0	M	H	0	0	0	0	H	0	0	0	general GO	136
proteolysis (297)	HNF1.03	0.31	0	0		0	0	0	0	0	0	0	0	0	general GO	137
transport (940)	HNF1.03	0.31	0	0		H	0	0	0	0	0	0	0	0	general GO	
blood coagulation (70)	HNF1.03	0.31	M	0		0	0	0	0	0	0	0	0	0	general GO	
sodium ion transport (75)	HNF1.03	0.31	0	0		0	0	0	0	0	0	0	0	0	general GO	
signal transduction (1189)	EVI1.01	0.31	0	0		0	0	0	0	0	0	0	0	0	general GO	138
G protein coupled receptor protein signaling pathway	EVI1.01	0.31	H	H		H	0	0	0	0	0	0	0	0	general GO	
cell cell signaling (268)	ATATA.01	0.3	0	0		0	0	0	0	0	0	0	0	0	general GO	139
immune response (366)	MEF2.02	0.3	0	0		H	0	0	0	0	0	0	0	0	general GO	140
proteolysis (297)	HNF1.01	0.3	0	0		0	0	0	0	0	0	0	0	0	general GO	141
transport (940)	HNF1.01	0.3	0	0		0	0	0	0	0	0	0	0	0	general GO	
immune response (366)	HNF1.01	0.3	M	0	H	H	0	0	0	0	0	0	0	0	general GO	
sodium ion transport (75)	HNF1.01	0.3	0	0		0	0	0	0	0	0	0	0	0	general GO	
inflammatory response (163)	MEF2.05	0.29	0	0		H	0	0	0	0	0	0	0	0	general GO	142
immune response (366)	MEF2.05	0.29	0	0	M	H	0	0	0	0	0	0	0	0	general GO	
inflammatory response (163)	RSRFC4.02	0.29	0	0		0	0	0	0	0	0	0	0	0	general GO	143
immune response (366)	RSRFC4.02	0.29	0	0		H	0	0	0	0	0	0	0	0	general GO	
immune response (366)	MEL1_01	0.29		0	H	H	0	0	0	0	0	0	H	H	general GO	144
digestion (51)	MEL1_01	0.29	M	0	0	0	H	0	0	0	0	0	0	0	general GO	
G protein coupled receptor protein signaling pathway	GATA3.02	0.29	0	0	H	H	M	0	H	0	0	0	0	0	general GO	145
sensory perception (201)	TATA.01	0.28	0	0		H	0	0	0	0	H	0	0	0	general GO	146
chemotaxis (98)	TATA.01	0.28	0	0		0	0	0	0	0	0	0	0	0	general GO	
inflammatory response (163)	TATA.01	0.28	0	0		H	0	0	0	0	0	0	0	0	general GO	
immune response (366)	TATA.01	0.28	0	0		H	0	0	0	0	0	0	0	0	general GO	
cell cell signaling (268)	TATA.01	0.28	0	H		0	0	0	0	0	0	0	0	0	general GO	
nucleosome assembly (71)	TATA.01	0.28	0	0		0	H	0	0	0	0	0	0	0	general GO	
chromosome organization and biogenesis (83)	TATA.01	0.28	0	0		0	H	0	0	0	0	0	0	0	general GO	

epidermis development (59)	TATA.01	0.28	0	0		0	0	0	0	0	0	0	0	0	general GO	
response to virus (55)	TATA.01	0.28	0	0		0	0	H	H	H	0	0	0	0	general GO	
calcium ion homeostasis (30)	TATA.01	0.28	0	H		0	0	0	0	0	0	0	0	0	general GO	
immune response (366)	RSRFC4.01	0.28	0	0		H	0	0	0	0	0	0	0	0	general GO	147
inflammatory response (163)	TATA.02	0.28	0	0		0	0	0	0	0	0	0	0	0	general GO	148
immune response (366)	TATA.02	0.28	0	0		H	0	0	0	0	0	0	0	0	general GO	
cell cell signaling (268)	TATA.02	0.28	0	H		0	0	0	0	0	0	0	0	0	general GO	
epidermis development (59)	TATA.02	0.28	0	0		0	0	0	0	0	0	0	0	0	general GO	
calcium ion homeostasis (30)	TATA.02	0.28	0	H	M	0	0	0	0	0	0	0	0	0	general GO	
inflammatory response (163)	PIT1.01	0.27	H	H		H	H	0	0	0	0	0	0	0	general GO	149
immune response (366)	PIT1.01	0.27	H	0		0	0	0	0	0	0	0	0	0	general GO	
innate immune response (46)	FREAC7.01	0.27	M	0	H	H	0	0	0	0	0	0	0	0	general GO	150
digestion (51)	FREAC7.01	0.27	0	0		0	0	0	0	0	0	0	0	0	general GO	
digestion (51)	HFH3.01	0.27	H	0		0	0	0	0	0	0	0	0	0	general GO	151
inflammatory response (163)	HFH1.01	0.27	0	0	M	0	H	0	0	0	0	0	0	0	general GO	152
immune response (366)	HFH8.01	0.27	H	M	0	H	0	0	0	0	0	0	0	0	general GO	153
digestion (51)	HFH8.01	0.27	H	0		0	0	0	0	0	0	0	0	0	general GO	
inflammatory response (163)	BRN5.01	0.26	0	0		H	0	0	0	0	0	0	0	0	general GO	154
immune response (366)	BRN5.01	0.26	H	0	M	0	0	0	0	0	0	0	0	0	general GO	
immune response (366)	HNF3B.01	0.26	H	0		0	0	0	0	0	0	0	0	0	general GO	155
digestion (51)	HNF3B.01	0.26	H	0	M	0	0	0	0	0	0	0	0	0	general GO	
digestion (51)	HFH2.01	0.25	H	0	M	0	0	0	0	0	0	0	0	0	general GO	156
immune response (366)	NKX31.01	0.25	0	0	M	H	0	0	0	0	0	0	0	0	general GO	157
immune response (366)	EVI1.03	0.24		0		0	0	0	0	0	0	0	0	H	general GO	158
G protein coupled receptor protein signaling pathway	EVI1.03	0.24	0	H		H	0	0	0	0	0	0	0	0	general GO	
immune response (366)	OCT1.06	0.22	H		H	H	0	0	H	0	0	0	0	0	general GO	159

development (530)	GC_rich	0.99	H	0	0	H		H	H	M	H	0	H	M	transcription GO
regulation of transcription (266)	GC_rich	0.99	H	0	H				H	0	0	H	0	0	transcription GO
regulation of transcription DNA dependent (1066)	GC_rich	0.99	H	0	0						H				transcription GO
transcription (930)	GC_rich	0.99	H	H	0						H				transcription GO
development (530)	AG_rich_coding	0.5	0	M	0	H	H	H	H	0	0	0	0	0	transcription GO
immune response (366)	poly_A	0.01	H	H			0	0	0	0	0	0	0	0	general GO
development (530)	poly_C	0.99	0	M	0		H	0	H	0	0	0	0	M	transcription GO
regulation of transcription (266)	poly_C	0.99	H	0	H		H	H	H	0	0	0	M	0	transcription GO
regulation of transcription DNA dependent (1066)	poly_C	0.99	H		H			H			H			H	transcription GO
transcription (930)	poly_C	0.99	H		H	H		H			H			H	transcription GO
potassium ion transport (108)	poly_C	0.99	0	0	H	0	0	H	M	0	0	0	H	0	general GO
regulation of transcription from RNA polymerase II	poly_C	0.99	H	0	0	0	0	0	0	M	0	0	0	0	general GO

200bp Window

GO group	Motifs	motif GC	-199 - 0	1 - 200	201 - 400	401 - 600	601 - 800	801 - 1000	GO-classes
transcription (930)	SP1.01	0.78	0	0			M		transcription GO
regulation_of_transcription_DNA_dependent (1066)	SP1.01	0.78	0	0			M		transcription GO
development (530)	SP1.01	0.78	0	0	0		0	0	transcription GO
transcription (930)	WT1.01	0.77	H	0			0		transcription GO
regulation_of_transcription_DNA_dependent (1066)	WT1.01	0.77		H			0		transcription GO
regulation_of_transcription (266)	WT1.01	0.77	0	H		M	H	0	transcription GO
development (530)	WT1.01	0.77	0	H				M	transcription GO
transcription (930)	HES1.02	0.76	0	0	0		0	0	transcription GO
regulation_of_transcription_DNA_dependent (1066)	HES1.02	0.76	0	M	0		0	0	transcription GO
transcription (930)	MAZR.01	0.76	H				M		transcription GO
regulation_of_transcription_DNA_dependent (1066)	MAZR.01	0.76	H						transcription GO
regulation_of_transcription (266)	MAZR.01	0.76	0	0			0	M	transcription GO
development (530)	MAZR.01	0.76	0	H			H	H	transcription GO
transcription (930)	ZNF202.01	0.73	H	H				M	transcription GO
regulation_of_transcription_DNA_dependent (1066)	ZNF202.01	0.73	H					M	transcription GO
regulation_of_transcription (266)	ZNF202.01	0.73	M	H			0	0	transcription GO

development (530)	ZNF202.01	0.73	0			H	H	M	transcription GO
transcription (930)	AP2.01	0.73	0	H					transcription GO
regulation_of_transcription_DNA_dependent (1066)	AP2.01	0.73	M	0					transcription GO
regulation_of_transcription (266)	AP2.01	0.73	0	0		M	H	H	transcription GO
development (530)	AP2.01	0.73	0	0		H	H	H	transcription GO
transcription (930)	ZF5.01	0.72	0	0			H	H	transcription GO
regulation_of_transcription_DNA_dependent (1066)	ZF5.01	0.72		M				H	transcription GO
regulation_of_transcription (266)	ZF5.01	0.72	0	H		0	H	0	transcription GO
transcription (930)	ZF9.01	0.72	0	0			M		transcription GO
regulation_of_transcription_DNA_dependent (1066)	ZF9.01	0.72	0	0					transcription GO
transcription (930)	EGR3.01	0.71	0	0	M		0	H	transcription GO
regulation_of_transcription_DNA_dependent (1066)	EGR3.01	0.71	0	0	M		0		transcription GO
transcription (930)	NRF1_01	0.71	H	0					transcription GO
regulation_of_transcription_DNA_dependent (1066)	NRF1_01	0.71	H	0				H	transcription GO
regulation_of_transcription_DNA_dependent (1066)	MYCMAX.03	0.7	0	0		H	0	0	transcription GO
transcription (930)	ZBP89.01	0.7	H			M	M	0	transcription GO
regulation_of_transcription_DNA_dependent (1066)	ZBP89.01	0.7	H	M		M		M	transcription GO
regulation_of_transcription (266)	ZBP89.01	0.7	M	M			0	0	transcription GO
development (530)	ZBP89.01	0.7	0	H				H	transcription GO
regulation_of_transcription_DNA_dependent (1066)	HELT.01	0.69	0	0	H	H		0	transcription GO
regulation_of_transcription_DNA_dependent (1066)	NGFIC.01	0.69	0	0	M	H	H		transcription GO
transcription (930)	CKROX_01	0.68					0	0	transcription GO
regulation_of_transcription_DNA_dependent (1066)	CKROX_01	0.68						M	transcription GO
regulation_of_transcription (266)	CKROX_01	0.68	0	M			0	0	transcription GO
development (530)	CKROX_01	0.68	0	H			H	0	transcription GO
transcription (930)	EGR1.02	0.68							transcription GO
regulation_of_transcription_DNA_dependent (1066)	EGR1.02	0.68		M					transcription GO
regulation_of_transcription_DNA_dependent (1066)	HIC1_01	0.68	0	0	0	0	H		transcription GO
transcription (930)	BKLF.01	0.68	0	0	0	0	0		transcription GO
regulation_of_transcription_DNA_dependent (1066)	BKLF.01	0.68	0	0	0	0	0		transcription GO
transcription (930)	GC.01	0.68	0	0			0	0	transcription GO
regulation_of_transcription_DNA_dependent (1066)	GC.01	0.68	0	0			0	M	transcription GO
development (530)	MZF1.01	0.68	0	H	H		H	H	transcription GO
regulation_of_transcription_DNA_dependent (1066)	EGR1.01	0.67	H	0	M	0	H		transcription GO
regulation_of_transcription_DNA_dependent (1066)	TIEG.01	0.66	0	0	H	0	0		transcription GO
transcription (930)	MAZ.01	0.65	0	0		M		H	transcription GO
regulation_of_transcription_DNA_dependent (1066)	MAZ.01	0.65	0	0			M		transcription GO
regulation_of_transcription (266)	MAZ.01	0.65	0	0			H	0	transcription GO
development (530)	MAZ.01	0.65	0	H	H			H	transcription GO
development (530)	ZIC2_01	0.65	0	0	H	0	0		transcription GO
transcription (930)	PLAG1_01	0.63	M	H	M		H	H	transcription GO
regulation_of_transcription_DNA_dependent (1066)	PLAG1_01	0.63	M	H	M				transcription GO
regulation_of_transcription (266)	PLAG1_01	0.63	M	0	H		0	0	transcription GO
development (530)	PLAG1_01	0.63	0	H		H	H	0	transcription GO
transcription (930)	HES1.01	0.62	0	M	M		H	H	transcription GO
regulation_of_transcription_DNA_dependent (1066)	HES1.01	0.62	0	M			H		transcription GO
transcription (930)	MUSCLE_INI.02	0.62	0	0		H	0	0	transcription GO
regulation_of_transcription_DNA_dependent (1066)	MUSCLE_INI.02	0.62	0	0		H	0	M	transcription GO
transcription (930)	CDE.01	0.6	H		M		M	H	transcription GO
regulation_of_transcription_DNA_dependent (1066)	CDE.01	0.6	H	M	M				transcription GO
development (530)	CDE.01	0.6	0	0	0	H	0		transcription GO
transcription (930)	E2F.02	0.59	0	H	0	0		M	transcription GO
regulation_of_transcription_DNA_dependent (1066)	E2F.02	0.59	0	H	0	0		M	transcription GO
development (530)	PAX5.01	0.57	0	0	0	0		0	transcription GO
regulation_of_transcription (266)	VDR_RXR.02	0.56	0	0	0		0	0	transcription GO
transcription (930)	GAGA.01	0.56		0	M		0	H	transcription GO
regulation_of_transcription_DNA_dependent (1066)	GAGA.01	0.56		H	H		0	H	transcription GO
regulation_of_transcription (266)	GAGA.01	0.56	H		H		0	0	transcription GO
development (530)	GAGA.01	0.56	M		H	H	H	H	transcription GO
regulation_of_transcription_DNA_dependent (1066)	E2F.03	0.54	0	0	0	0	M		transcription GO
regulation_of_transcription_DNA_dependent (1066)	NFY.01	0.52	0	H		0	0	0	transcription GO
regulation_of_transcription_DNA_dependent (1066)	AG_rich_coding	0.5	M		H		0	0	transcription GO
regulation_of_transcription (266)	AG_rich_coding	0.5	M		H		H	0	transcription GO
development (530)	AG_rich_coding	0.5			H	H	0	0	transcription GO

development (530)	MTATA.01	0.47	0		0	0	0	0	transcription GO
regulation_of_transcription (266)	LMX1B.01	0.32		0	0	0	0	0	transcription GO
development (530)	TATA.01	0.28	0		0	0	0	0	transcription GO
small_GTPase_mediated_signal_transduction (124)	SP1.01	0.78			H	0	0	0	general GO
protein_transport (254)	SP1.01	0.78		H	0	0	0	0	general GO
intracellular_protein_transport (191)	SP1.01	0.78		H	0	0	0	0	general GO
Wnt_receptor_signaling_pathway (66)	WT1.01	0.77	0		0	0	H	0	general GO
small_GTPase_mediated_signal_transduction (124)	WT1.01	0.77		H	H	0	0	0	general GO
cation_transport (99)	ZNF202.01	0.73	0		0	0	0	0	general GO
small_GTPase_mediated_signal_transduction (124)	ZF9.01	0.72		H	0	0	0	0	general GO
protein_transport (254)	ZF9.01	0.72		H	0	0	0	0	general GO
intracellular_protein_transport (191)	ZF9.01	0.72		H	0	0	0	0	general GO
chromatin_modification (53)	CKROX_01	0.68	0		H	0	0	0	general GO
small_GTPase_mediated_signal_transduction (124)	EGR1.02	0.68			0	0	0	0	general GO
protein_transport (254)	EGR1.02	0.68		H	0	0	0	0	general GO
intracellular_protein_transport (191)	EGR1.02	0.68		H	0	0	0	0	general GO
muscle_development (107)	INSM1_01	0.63		0	0	0	0	0	general GO
protein_biosynthesis (197)	GABP.01	0.62	H		0	0	0	0	general GO
protein_complex_assembly (94)	P53.01	0.61	0	0	0	0	0		general GO
inflammatory_response (163)	NFKAPPAB.01	0.6	0		0	0	0	0	general GO
immune_response (366)	NFKAPPAB.01	0.6	0		0	0	0	0	general GO
chemotaxis (98)	NFKAPPAB.01	0.6	0		0	0	0	0	general GO
transcription_RNA_polyII_promoter (190)	CDE.01	0.6	0	0	0	0		0	general GO
response_to_DNA_damage_stimulus (117)	E2F.02	0.59		0	M	0	0	0	general GO
DNA_repair (148)	E2F.02	0.59		H	M	0	0	0	general GO
protein_transport (254)	WHN.01	0.59	0		0	0	0	0	general GO
protein_biosynthesis (197)	WHN.01	0.59	0			0	0	0	general GO
cell_division (103)	WHN.01	0.59	0		0	0	0	0	general GO
cell_cycle (273)	WHN.01	0.59	0	H		0	0	0	general GO
RNA_splicing (61)	NRF2.01	0.57	H		0	0	0	0	general GO
protein_transport (254)	NRF2.01	0.57		0	0	0	0	0	general GO
protein_biosynthesis (197)	NRF2.01	0.57			0	0	0	0	general GO
sensory_perception (201)	NFKAPPAB.02	0.57	0		0	0	0	0	general GO
inflammatory_response (163)	NFKAPPAB.02	0.57	0		0	0	0	0	general GO
immune_response (366)	NFKAPPAB.02	0.57	0		0	0	0	0	general GO
chemotaxis (98)	NFKAPPAB.02	0.57	0		0	0	0	0	general GO
muscle_contraction (78)	MYOD.02	0.56	0		0	0	0	0	general GO
inflammatory_response (163)	NFKAPPAB65.01	0.55	0		0	0	0	0	general GO
immune_response (366)	NFKAPPAB65.01	0.55	0		0	0	0	0	general GO
chemotaxis (98)	NFKAPPAB65.01	0.55	0		0	0	0	0	general GO
cell_surface_receptor_linked_signal_transduction (169)	NFKAPPAB65.01	0.55	0		0	0	0	0	general GO
cell_cell_signaling (268)	NFKAPPAB65.01	0.55	0		0	0	0	0	general GO
apoptosis (258)	NFKAPPAB65.01	0.55	0		H	0	0	0	general GO
protein_biosynthesis (197)	CETS1P54.01	0.55	0		0	0	0	0	general GO
protein_biosynthesis (197)	YY1.01	0.55		0	0	0	0	0	general GO
inflammatory_response (163)	CREL.01	0.55	0		0	0	0	0	general GO
immune_response (366)	CREL.01	0.55	0		0	0	0	0	general GO
chemotaxis (98)	CREL.01	0.55	0		0	0	0	0	general GO
cell_cell_signaling (268)	CREL.01	0.55	0		0	0	0	0	general GO
protein_biosynthesis (197)	ELK1.02	0.55	H		0	0	0	0	general GO
response_to_virus (55)	NFKAPPAB.03	0.55	0		0	0	0	0	general GO
inflammatory_response (163)	NFKAPPAB.03	0.55	0		0	0	0	0	general GO
immune_response (366)	NFKAPPAB.03	0.55	0		0	0	0	0	general GO
chemotaxis (98)	NFKAPPAB.03	0.55	0		0	0	0	0	general GO
cellular_defense_response (68)	NFKAPPAB.03	0.55	0		0	0	0	0	general GO
cell_cell_signaling (268)	NFKAPPAB.03	0.55	0		0	0	H	0	general GO
response_to_DNA_damage_stimulus (117)	FLI.01	0.53	0		0	0	0	0	general GO
protein_biosynthesis (197)	FLI.01	0.53	H		0	0	0	0	general GO
DNA_repair (148)	FLI.01	0.53	0		0	0	0	0	general GO
G_protein_coupled_receptor_protein_signaling_pathwa	NF1.01	0.53		0	0	0	0	0	general GO
metabolism (241)	HNF4.01	0.53	0		0	0	0	0	general GO
electron_transport (214)	HNF4.01	0.53	0		0	0	0	0	general GO
inflammatory_response (163)	HIVEP1_01	0.52	M		0	0	0	0	general GO
immune_response (366)	HIVEP1_01	0.52	0		0	0	0	0	general GO

chemotaxis (98)	HIVEP1_01	0.52	0		0	0	0	0	general GO
cell_cell_signaling (268)	HIVEP1_01	0.52	0		0	0	0	0	general GO
immune_response (366)	AML1.01	0.52	0		0	0	0	0	general GO
small_GTPase_mediated_signal_transduction (124)	ERR_01	0.52	0	0		0	0	0	general GO
steroid_biosynthesis (42)	NFY.01	0.52	0		0	0	0	0	general GO
mitosis (86)	NFY.01	0.52	0		0	0	0	0	general GO
cell_division (103)	NFY.01	0.52	0		H	0	0	0	general GO
cell_cycle (273)	NFY.01	0.52	0		H	0	0	0	general GO
immune_response (366)	ETS1.01	0.52	0		0	0	0	0	general GO
cellular_defense_response (68)	ETS1.01	0.52	0		0	0	0	0	general GO
protein_biosynthesis (197)	ELK1.01	0.52	H		0	0	0	0	general GO
immune_response (366)	AML3.01	0.5	H		0	H	0	0	general GO
immune_response (366)	RBPJK.02	0.5	H		0	0	0	0	general GO
skeletal_development (83)	AG_rich_coding	0.5	0		0	0	0	0	general GO
muscle_development (107)	TAL1ALPHAE47.01	0.5	0		0	0	0	0	general GO
muscle_development (107)	SRF.02	0.49	0		H	0	0	0	general GO
immune_response (366)	TR2.01	0.49		0	H	0	0	0	general GO
steroid_biosynthesis (42)	CAAT.01	0.49	0		0	0	0	0	general GO
mitosis (86)	CAAT.01	0.49	0		0	0	0	0	general GO
chromosome_organization_and_biogenesis (83)	CAAT.01	0.49	0		0	0	0	0	general GO
cell_division (103)	CAAT.01	0.49	0		0	0	0	0	general GO
cell_cycle (273)	CAAT.01	0.49	0		0	0	0	0	general GO
immune_response (366)	TAL1BETAE47.01	0.49	M	0		M	0	0	general GO
chemotaxis (98)	IK3.01	0.49	0		0	0	0	0	general GO
sensory_perception (201)	IK2.01	0.49		0	0	0	0	0	general GO
proteolysis (297)	AREB6.02	0.48	0		0	0	0	0	general GO
immune_response (366)	PU1.01	0.48	0		0	0	0	0	general GO
cell_surface_receptor_linked_signal_transduction (169)	PU1.01	0.48	0		0	0	0	0	general GO
inflammatory_response (163)	ETS2.01	0.48	0		0	0	0	0	general GO
immune_response (366)	ETS2.01	0.48	H		0	0	0	0	general GO
chemotaxis (98)	ETS2.01	0.48	0		0	0	0	0	general GO
cell_surface_receptor_linked_signal_transduction (169)	ETS2.01	0.48	0		0	0	0	0	general GO
steroid_biosynthesis (42)	NFY.02	0.48	0		0	0	0	0	general GO
mitosis (86)	NFY.02	0.48	0		0	0	0	0	general GO
chromosome_organization_and_biogenesis (83)	NFY.02	0.48	0		0	0	0	0	general GO
cell_cycle (273)	NFY.02	0.48	0		0	0	0	0	general GO
biosynthesis (32)	NFY.02	0.48	0		0	0	0	0	general GO
immune_response (366)	PAX2.01	0.47	0		0	0	0	0	general GO
immune_response (366)	PPARA.01	0.47		H	0	0	0	0	general GO
immune_response (366)	NFE2L2.01	0.47			H	0	0	0	general GO
muscle_development (107)	MTATA.01	0.47	0		0	0	0	0	general GO
nucleosome_assembly (71)	NFY.03	0.47	0		0	0	0	0	general GO
mitosis (86)	NFY.03	0.47	0		0	0	0	0	general GO
chromosome_organization_and_biogenesis (83)	NFY.03	0.47	0		0	0	0	0	general GO
cell_division (103)	NFY.03	0.47	0		0	0	0	0	general GO
cell_cycle (273)	NFY.03	0.47	0		0	0	0	0	general GO
muscle_development (107)	SRF.03	0.47	H		H	0	0	0	general GO
lipid_transport (41)	SRF.03	0.47		0	0	0	0	0	general GO
immune_response (366)	BACH1.01	0.47	H			0	0	0	general GO
G_protein_coupled_receptor_protein_signaling_pathwa	PLZF.01	0.47		0	0	0	0	0	general GO
immune_response (366)	ARE.01	0.47	H		0	0	0	0	general GO
sensory_perception (201)	MEL1_02	0.46	H		H	0	0	0	general GO
immune_response (366)	STAT6.01	0.46	H		H	0	0	0	general GO
sensory_perception_of_smell (40)	MIT.01	0.46		0	0	0	0	0	general GO
immune_response (366)	MIT.01	0.46		H	M	0	0	0	general GO
immune_response (366)	RP58.01	0.46		0	0	0	0	0	general GO
inflammatory_response (163)	COMP1.01	0.45	H		0	0	0	0	general GO
immune_response (366)	PXRCAR.01	0.45	0		0	0	0	0	general GO
inflammatory_response (163)	DBP.01	0.45	0		0	0	0	0	general GO
inflammatory_response (163)	BARBIE.01	0.45	0		0	0	0	0	general GO
G_protein_coupled_receptor_protein_signaling_pathwa	BRACH.01	0.45	0		0	0	0	0	general GO
visual_perception (142)	PTX1.01	0.45	0		H	0	0	0	general GO
immune_response (366)	PAX8.01	0.45	0		0	0	0	0	general GO
cell_surface_receptor_linked_signal_transduction (169)	GATA1.01	0.45	H		0	0	0	0	general GO

innate_immune_response (46)	STAT.01	0.44	0		0	0	0	0	general GO
immune_response (366)	MYT1L.01	0.44	H		0	0	0	0	general GO
immune_response (366)	STAT.01	0.44	0		0	0	0	0	general GO
immune_response (366)	TCF11.01	0.44	0	H		0	0	0	general GO
inflammatory_response (163)	BCL6.02	0.44	0		H	0	0	0	general GO
immune_response (366)	BCL6.02	0.44	0		0	0	0	0	general GO
inflammatory_response (163)	TCF11MAFG.01	0.43		H	0	H	0	0	general GO
immune_response (366)	TCF11MAFG.01	0.43			0	0	0	0	general GO
G_protein_coupled_receptor_protein_signaling_pathwa	TCF11MAFG.01	0.43	0		H	H	0	0	general GO
immune_response (366)	ILF1_01	0.43	0		H	0	0	0	general GO
signal_transduction (1189)	AP1.01	0.43	0		0	0	0	0	general GO
proteolysis (297)	AP1.01	0.43	0		0	0	0	0	general GO
inflammatory_response (163)	AP1.01	0.43	M		0	0	0	0	general GO
immune_response (366)	AP1.01	0.43	H		H	0	0	0	general GO
chemotaxis (98)	AP1.01	0.43	M		H	0	0	0	general GO
response_to_virus (55)	ISRE.01	0.43	0		H	0	0	0	general GO
inflammatory_response (163)	ISRE.01	0.43	0		0	0	0	0	general GO
immune_response (366)	ISRE.01	0.43			0	0	0	0	general GO
response_to_virus (55)	IRF2.01	0.43	0	H		0	0	0	general GO
immune_response (366)	IRF2.01	0.43			0	0	0	0	general GO
response_to_virus (55)	IRF3.01	0.42	0		0	0	0	0	general GO
inflammatory_response (163)	IRF3.01	0.42	0		0	0	0	0	general GO
immune_response (366)	IRF3.01	0.42	H		0	0	0	0	general GO
sensory_perception (201)	SRF.01	0.42	H		0	0	0	0	general GO
muscle_development (107)	SRF.01	0.42	0		0	0	0	0	general GO
inflammatory_response (163)	SRF.01	0.42	0		0	0	0	0	general GO
immune_response (366)	SRF.01	0.42	H		M	0	0	0	general GO
chemotaxis (98)	SRF.01	0.42	0		0	0	0	0	general GO
inflammatory_response (163)	PRDM1.01	0.41	M		0	0	0	0	general GO
immune_response (366)	PRDM1.01	0.41	M		0	0	0	0	general GO
defense_response (100)	PRDM1.01	0.41	0		0	0	0	0	general GO
inflammatory_response (163)	HMGIY.01	0.41	0		0	0	0	0	general GO
immune_response (366)	HMGIY.01	0.41	H		0	H	0	0	general GO
chemotaxis (98)	HMGIY.01	0.41	0		H	0	0	0	general GO
pregnancy (41)	PDX1_Gabi	0.41	0		0	0	0	0	general GO
immune_response (366)	HBP1_01	0.41		H	0	0	0	0	general GO
sensory_perception (201)	GFI1.01	0.41	0		H	0	0	0	general GO
immune_response (366)	PSE_02	0.41	0		0	0	0	0	general GO
mitosis (86)	CHR.01	0.41	H		0	0	0	0	general GO
innate_immune_response (46)	BRN4.01	0.4		0	0	0	0	0	general GO
immune_response (366)	BRN4.01	0.4		0	H	0	0	0	general GO
immune_response (366)	LEF1.02	0.4		H	H	0	0	0	general GO
cell_cell_signaling (268)	MTBF.01	0.4	0		0	0	0	0	general GO
response_to_virus (55)	IRF1.01	0.4	0			0	H	0	general GO
inflammatory_response (163)	IRF1.01	0.4	0		0	0	0	0	general GO
immune_response (366)	IRF1.01	0.4			0	0	0	0	general GO
response_to_virus (55)	CEBPB.01	0.4	0		0	0	0	0	general GO
proteolysis (297)	CEBPB.01	0.4	0		0	0	0	0	general GO
inflammatory_response (163)	CEBPB.01	0.4	0		H	0	0	0	general GO
immune_response (366)	GATA1.04	0.4		H	H	0	0	0	general GO
defense_response (100)	CEBPB.01	0.4	0		0	0	0	0	general GO
visual_perception (142)	CRX.01	0.39	0		0	0	0	0	general GO
sensory_perception (201)	CRX.01	0.39	0		H	0	0	0	general GO
immune_response (366)	OCT1.05	0.39		H	H	0	0	0	general GO
sensory_perception (201)	AARE.01	0.39	0	H	0		0	0	general GO
inflammatory_response (163)	AARE.01	0.39	0		0	0	0	0	general GO
immune_response (366)	AARE.01	0.39	0		0	0	0	0	general GO
cell_cell_signaling (268)	AARE.01	0.39	0		0	0	0	0	general GO
inflammatory_response (163)	LTATA_01	0.39	0		0	0	0	0	general GO
cell_cell_signaling (268)	LTATA_01	0.39	0		0	0	0	0	general GO
antimicrobial_humoral_response (87)	LTATA_01	0.39	0		0	0	0	0	general GO
inflammatory_response (163)	BRN2.01	0.39	0		0	0	0	0	general GO
immune_response (366)	BRN2.01	0.39	H		H	0	H	0	general GO
response_to_virus (55)	IRF4.01	0.39	0		0	0	0	0	general GO

inflammatory_response (163)	IRF4.01	0.39	M		0	0	0	0	general GO
immune_response (366)	IRF4.01	0.39			0	0	0	0	general GO
visual_perception (142)	OTX2.01	0.39	0		0	0	0	0	general GO
sensory_perception (201)	OTX2.01	0.39	0		H	0	0	0	general GO
inflammatory_response (163)	STAT5.01	0.39	0		0	0	0	0	general GO
immune_response (366)	STAT5.01	0.39	0			0	0	0	general GO
immune_response (366)	GATA1.03	0.39		H	H	0	0	0	general GO
chemotaxis (98)	GATA1.03	0.39	H		0	0	H	0	general GO
antimicrobial_humoral_response (87)	GATA1.03	0.39		H	H	0	0	0	general GO
immune_response (366)	BCL6.01	0.39	0			H	0	0	general GO
immune_response (366)	OCT1.04	0.38		H	H	0	0	0	general GO
immune_response (366)	GFI1B.01	0.38	0		0	0	H	0	general GO
nucleosome_assembly (71)	PDX1.01	0.38	0	H	H	0		0	general GO
muscle_development (107)	AMEF2.01	0.38	0		0	0	0	0	general GO
immune_response (366)	AMEF2.01	0.38	0		0	0	0	0	general GO
immune_response (366)	RORA2.01	0.38		H	H	H	0	0	general GO
inflammatory_response (163)	NFAT.01	0.38	M		H	0	0	0	general GO
immune_response (366)	NFAT.01	0.38			0	0	0	0	general GO
chemotaxis (98)	NFAT.01	0.38	0		0	0	0	0	general GO
cell_surface_receptor_linked_signal_transduction (169)	NFAT.01	0.38	0		0	0	0	0	general GO
cell_cell_signaling (268)	NFAT.01	0.38	0		0	0	0	0	general GO
G_protein_coupled_receptor_protein_signaling_pathwa	FREAC4.01	0.38	M		0	0	0	0	general GO
innate_immune_response (46)	CDX2.01	0.38		H	0	0	H	H	general GO
inflammatory_response (163)	CDX2.01	0.38	H		0	H	H	0	general GO
immune_response (366)	CDX2.01	0.38	H		H	0	0	0	general GO
chemotaxis (98)	CDX2.01	0.38	0		0	0	0	0	general GO
cell_cell_signaling (268)	CDX2.01	0.38	0		0	0	0	0	general GO
immune_response (366)	MEF2.01	0.38			0	0	0	0	general GO
inflammatory_response (163)	MMEF2.01	0.37	0		0	0	H	0	general GO
immune_response (366)	MMEF2.01	0.37	0		0	0	H	0	general GO
sensory_perception (201)	GATA2.02	0.36	0		H	H	0	0	general GO
immune_response (366)	GATA2.02	0.36	H		H	0	H	0	general GO
immune_response (366)	GATA1.05	0.36		H		0	0	0	general GO
sensory_perception (201)	XFD3.01	0.36	0		0	0	0	0	general GO
G_protein_coupled_receptor_protein_signaling_pathwa	XFD3.01	0.36	H		0	0	H	0	general GO
response_to_virus (55)	IRF7.01	0.36	H		0	0	0	0	general GO
inflammatory_response (163)	IRF7.01	0.36	M		0	0	0	0	general GO
immune_response (366)	IRF7.01	0.36	H		H	0	0	0	general GO
sensory_perception (201)	OCT1P.01	0.36	H		H	H	0	0	general GO
immune_response (366)	OCT1P.01	0.36		H	H	H	0	0	general GO
innate_immune_response (46)	OCT.01	0.35		H	0	0	0	0	general GO
immune_response (366)	OCT.01	0.35		H	H	0	0	0	general GO
visual_perception (142)	SIX3.01	0.35	0		H	0	0	H	general GO
sensory_perception (201)	SIX3.01	0.35	0		H	0	0	0	general GO
immune_response (366)	GATA2.01	0.35			0	M	0	0	general GO
inflammatory_response (163)	CDX1.01	0.35	M		0	0	0	0	general GO
immune_response (366)	CDX1.01	0.35	H		0	0	0	0	general GO
immune_response (366)	NKX25.02	0.35		H	H	0	0	0	general GO
immune_response (366)	OCT1.02	0.3		H	0	0	0	0	general GO
inflammatory_response (163)	DLX3.01	0.34	0		0	0	0	0	general GO
innate_immune_response (46)	HNF1.02	0.34	H		0	0	H	0	general GO
blood_coagulation (70)	HNF1.02	0.34	0		0	0	0	0	general GO
inflammatory_response (163)	XFD1.01	0.34	0		0	0	0	0	general GO
immune_response (366)	XFD1.01	0.34		H	0	0	0	0	general GO
immune_response (366)	HMEF2.01	0.34	H		0	0	0	0	general GO
sensory_perception (201)	GATA3.01	0.34	H		H	0	H	0	general GO
inflammatory_response (163)	GATA3.01	0.34	H		0	0	H	0	general GO
chemotaxis (98)	GATA3.01	0.34			0	0	H	0	general GO
inflammatory_response (163)	PAX4.01	0.33	0		0	0	0	0	general GO
inflammatory_response (163)	EVI1.04	0.33	0		0	0	0	0	general GO
immune_response (366)	EVI1.04	0.33	H		0	H	0	0	general GO
signal_transduction (1189)	EVI1.02	0.33	M		0	0	0	0	general GO
sensory_perception (201)	EVI1.02	0.33	0		H	H	0	0	general GO
immune_response (366)	EVI1.02	0.33		H	H	0	0	0	general GO

G_protein_coupled_receptor_protein_signaling_pathwa	EVI1.02	0.33	0		H	0	0	0	general GO
inflammatory_response (163)	BRN3.02	0.33	0		0	0	H	0	general GO
immune_response (366)	BRN2.03	0.32	H		H	0	0	0	general GO
chromosome_organization_and_biogenesis (83)	BRN2.03	0.32	0		H	0	H	0	general GO
sensory_perception (201)	XFD2.01	0.32	0		H	0	H	0	general GO
immune_response (366)	XFD2.01	0.32	M		H	0	0	0	general GO
inflammatory_response (163)	LMX1B.01	0.32	0		0	0	H	0	general GO
immune_response (366)	LMX1B.01	0.32	0		H	0	0	0	general GO
proteolysis (297)	FREAC3.01	0.32	M		0	0	0	0	general GO
inflammatory_response (163)	CART1.01	0.32	0		0	0	0	0	general GO
immune_response (366)	CART1.01	0.32	H		0	0	0	0	general GO
cell_surface_receptor_linked_signal_transduction (169)	EN1.01	0.32	0		0	0	0	0	general GO
transport (940)	HNF1.03	0.31	0		0	0	0	0	general GO
sodium_ion_transport (75)	HNF1.03	0.31	0		0	0	0	0	general GO
immune_response (366)	HNF1.03	0.31	H		0	0	0	0	general GO
blood_coagulation (70)	HNF1.03	0.31	0		0	0	0	0	general GO
signal_transduction (1189)	EVI1.01	0.31	M		0	0	0	0	general GO
G_protein_coupled_receptor_protein_signaling_pathwa	EVI1.01	0.31	H		0	0	0	0	general GO
synaptic_transmission (153)	MSX.01	0.3	0		0	0	0	0	general GO
immune_response (366)	ATATA.01	0.3	0		0	0	0	0	general GO
immune_response (366)	EVI1.06	0.3		H	H	H	0	0	general GO
cell_cell_signaling (268)	ATATA.01	0.3	0		0	0	0	0	general GO
muscle_development (107)	MEF2.02	0.3	0		0	0	0	0	general GO
inflammatory_response (163)	MEF2.02	0.3	0		0	0	0	0	general GO
immune_response (366)	MEF2.02	0.3	0		0	0	0	0	general GO
inflammatory_response (163)	MEF2.03	0.3	0		0	0	0	0	general GO
immune_response (366)	MEF2.03	0.3	H		0	0	0	0	general GO
transport (940)	HNF1.01	0.3	0		0	0	0	0	general GO
sodium_ion_transport (75)	HNF1.01	0.3	0		0	0	0	0	general GO
proteolysis (297)	HNF1.01	0.3	H		0	0	0	0	general GO
innate_immune_response (46)	HNF1.01	0.3		H	0	0	H	0	general GO
immune_response (366)	HNF1.01	0.3		H	0	0	0	0	general GO
blood_coagulation (70)	HNF1.01	0.3	H		0	0	0	0	general GO
immune_response (366)	MEF2.05	0.29			0	0	0	0	general GO
inflammatory_response (163)	RSRFC4.02	0.29	0		0	0	0	0	general GO
immune_response (366)	RSRFC4.02	0.29	H		0	0	0	0	general GO
immune_response (366)	MEL1_01	0.29		H		0	0	H	general GO
immune_response (366)	MEF2.04	0.29	H		0	0	0	0	general GO
immune_response (366)	EVI1.05	0.29		H	H	0	0	0	general GO
G_protein_coupled_receptor_protein_signaling_pathwa	EVI1.05	0.29	H		0	0	0	0	general GO
sensory_perception (201)	TATA.01	0.28	0		0	0	H	0	general GO
inflammatory_response (163)	TATA.01	0.28	0		0	0	0	0	general GO
immune_response (366)	TATA.01	0.28	M		0	0	0	0	general GO
epidermis_development (59)	TATA.01	0.28	0		0	0	0	0	general GO
digestion (51)	TATA.01	0.28	0		0	0	0	0	general GO
chromosome_organization_and_biogenesis (83)	TATA.01	0.28	0		H	0	0	0	general GO
chemotaxis (98)	TATA.01	0.28	0		0	0	0	0	general GO
cell_differentiation (192)	TATA.01	0.28	0		0	0	0	0	general GO
cell_cell_signaling (268)	TATA.01	0.28	H		0	0	0	0	general GO
calcium_ion_homeostasis (30)	TATA.01	0.28	0		0	0	0	0	general GO
immune_response (366)	RSRFC4.01	0.28	0		0	0	0	0	general GO
inflammatory_response (163)	TATA.02	0.28	0		0	0	0	0	general GO
immune_response (366)	TATA.02	0.28	0		0	0	0	0	general GO
epidermis_development (59)	TATA.02	0.28	0		0	0	0	0	general GO
cell_cell_signaling (268)	TATA.02	0.28	0		0	0	0	0	general GO
inflammatory_response (163)	MYT1.02	0.28	0		0	0	0	0	general GO
immune_response (366)	MYT1.02	0.28	H		H	0	0	0	general GO
inflammatory_response (163)	PIT1.01	0.27	H		0	0	0	0	general GO
immune_response (366)	PIT1.01	0.27	H		H	0	0	0	general GO
blood_coagulation (70)	SATB1.01	0.27		0	0	0	0	0	general GO
inflammatory_response (163)	HFH1.01	0.27	0		0	0	0	0	general GO
proteolysis (297)	HFH8.01	0.27	0		0	0	0	0	general GO
immune_response (366)	HFH8.01	0.27		H	0	0	0	0	general GO
inflammatory_response (163)	MYT1.01	0.27	M		H	0	0	0	general GO

immune_response (366)	OCT1.01	0.26		H	0	H	0	0	general GO
immune_response (366)	BRN5.01	0.26	H		0	0	0	0	general GO
steroid_metabolism (54)	HNF3B.01	0.26	0		0	0	0	0	general GO
immune_response (366)	NKX31.01	0.25	H		0	0	0	0	general GO
immune_response (366)	EVI1.03	0.24		H	H	0	0	H	general GO
G_protein_coupled_receptor_protein_signaling_pathwa	EVI1.03	0.24	H		H	0	0	0	general GO
immune_response (366)	BRIGHT.01	0.22	0		0	0	0	0	general GO
innate_immune_response (46)	OCT1.06	0.22		H	0	0	0	0	general GO
inflammatory_response (163)	OCT1.06	0.22	H		0	M	0	0	general GO
immune_response (366)	OCT1.06	0.22			0	0	0	0	general GO
proteolysis (297)	LHX3.01	0.14		H	0	0	0	0	general GO
immune_response (366)	LHX3.01	0.14	0		0	0	0	0	general GO

inflammatory_response (163)	poly_A	0.01	0		0	0	0	0	
immune_response (366)	poly_A	0.01	H		0	0	0	0	
transcription (930)	GC_rich	0.99	H						&H
regulation_of_transcription_DNA_dependent (1066)	GC_rich	0.99		M					&H
development (530)	GC_rich	0.99	0	0		M	0	M	
ubiquitin_cycle (154)	GC_rich	0.99		H	0	0	0	0	
intracellular_protein_transport (191)	GC_rich	0.99		H	0	0	0	0	
transcription (930)	poly_C	0.99					M		&H
regulation_of_transcription_DNA_dependent (1066)	poly_C	0.99							&H
regulation_of_transcription (266)	poly_C	0.99	M	0			0	M	
development (530)	poly_C	0.99	M	H		H		M	
Wnt_receptor_signaling_pathway (66)	poly_C	0.99	0		H	H	0	0	
transcription_RNA_polyII_promoter (190)	poly_C	0.99	0	0		0	0	0	
potassium_ion_transport (108)	poly_C	0.99	0	H	H		0	0	

300bp Window

GO group	Motifs	motif GC	-199 - 100	101 - 400	401 - 700	701 - 1000	GO-classes
transcription (930)	GC_rich	0.99					transcription GO
transcription (930)	poly_C	0.99					transcription GO
transcription (930)	SP1.01	0.78	H				transcription GO
transcription (930)	WT1.01	0.77	H				transcription GO
transcription (930)	HES1.02	0.76	0			H	transcription GO
transcription (930)	MAZR.01	0.76					transcription GO
transcription (930)	ZNF202.01	0.73					transcription GO
transcription (930)	AP2.01	0.73	H				transcription GO
transcription (930)	ZF5.01	0.72	H			H	transcription GO
transcription (930)	ZF9.01	0.72	H				transcription GO
transcription (930)	EGR3.01	0.71	H				transcription GO
transcription (930)	NRF1_01	0.71	H				transcription GO
transcription (930)	MYCMAX.03	0.7	0		H	H	transcription GO
transcription (930)	ZBP89.01	0.7				H	transcription GO
transcription (930)	HELT.01	0.69	H	H		H	transcription GO
transcription (930)	NGFIC.01	0.69	H		H	H	transcription GO
transcription (930)	CKROX_01	0.68					transcription GO
transcription (930)	EGR1.02	0.68					transcription GO
transcription (930)	HIC1_01	0.68	0	H	0		transcription GO
transcription (930)	BKLF.01	0.68	0	0	H		transcription GO
transcription (930)	GC.01	0.68	0	H			transcription GO
transcription (930)	MZF1.01	0.68	H	M		H	transcription GO
transcription (930)	MTF-1.01	0.65	0		0	0	transcription GO
transcription (930)	MAZ.01	0.65	H				transcription GO
transcription (930)	ZIC2_01	0.65	H	H	0		transcription GO
transcription (930)	ATF6.01	0.64	0		H	0	transcription GO
transcription (930)	PLAG1_01	0.63					transcription GO
transcription (930)	EBVR.01	0.63	H	0	0		transcription GO
transcription (930)	AHRARNT.0	0.62	0	H	H		transcription GO
transcription (930)	HES1.01	0.62	H		H	H	transcription GO
transcription (930)	MUSCLE_INI	0.62	0		H	H	transcription GO
transcription (930)	GABP.01	0.62	0	M	H	H	transcription GO
transcription (930)	MUSCLE_INI	0.61	0		H	0	transcription GO
transcription (930)	CDE.01	0.6					transcription GO
transcription (930)	AHR.01	0.59	H	H	H	M	transcription GO
transcription (930)	E2F.02	0.59		H			transcription GO
transcription (930)	WHN.01	0.59	H	H	0		transcription GO
transcription (930)	PAX5.01	0.57	0	H	M	H	transcription GO
transcription (930)	GAGA.01	0.56				H	transcription GO
transcription (930)	E2F.03	0.54	H	H			transcription GO
transcription (930)	AC_rich_codi	0.5		0	0	H	transcription GO
transcription (930)	AG_rich_cod	0.5			M	0	transcription GO
transcription (930)	GKLF.01	0.49	M	H	0	0	transcription GO
transcription (930)	CAAT.01	0.49	0	H	M	0	transcription GO
transcription (930)	NFY.02	0.48	0	H	M	0	transcription GO
regulation_of_transcription_from_RNA_polymerase_	CDE.01	0.6	H	H	H		transcription GO
regulation_of_transcription_DNA_dependent (1066)	GC_rich	0.99					transcription GO
regulation_of_transcription_DNA_dependent (1066)	poly_C	0.99					transcription GO
regulation_of_transcription_DNA_dependent (1066)	SP1.01	0.78	H				transcription GO
regulation_of_transcription_DNA_dependent (1066)	WT1.01	0.77					transcription GO
regulation_of_transcription_DNA_dependent (1066)	HES1.02	0.76	0			H	transcription GO

regulation_of_transcription_DNA_dependent (1066)	MAZR.01	0.76					transcription GO
regulation_of_transcription_DNA_dependent (1066)	ZNF202.01	0.73					transcription GO
regulation_of_transcription_DNA_dependent (1066)	AP2.01	0.73					transcription GO
regulation_of_transcription_DNA_dependent (1066)	ZF5.01	0.72				H	transcription GO
regulation_of_transcription_DNA_dependent (1066)	ZF9.01	0.72	H				transcription GO
regulation_of_transcription_DNA_dependent (1066)	EGR3.01	0.71	H				transcription GO
regulation_of_transcription_DNA_dependent (1066)	NRF1_01	0.71	H				transcription GO
regulation_of_transcription_DNA_dependent (1066)	MYCMAX.03	0.7	0		H	H	transcription GO
regulation_of_transcription_DNA_dependent (1066)	NFKAPPAB5	0.7	0	M	0		transcription GO
regulation_of_transcription_DNA_dependent (1066)	ZBP89.01	0.7					transcription GO
regulation_of_transcription_DNA_dependent (1066)	HELT.01	0.69	H			H	transcription GO
regulation_of_transcription_DNA_dependent (1066)	NGFIC.01	0.69	H		H		transcription GO
regulation_of_transcription_DNA_dependent (1066)	CKROX_01	0.68					transcription GO
regulation_of_transcription_DNA_dependent (1066)	EGR1.02	0.68					transcription GO
regulation_of_transcription_DNA_dependent (1066)	HIC1_01	0.68	0	0	H		transcription GO
regulation_of_transcription_DNA_dependent (1066)	BKLF.01	0.68	0	0	H		transcription GO
regulation_of_transcription_DNA_dependent (1066)	EGR2.01	0.68	H	H	H		transcription GO
regulation_of_transcription_DNA_dependent (1066)	GC.01	0.68	0				transcription GO
regulation_of_transcription_DNA_dependent (1066)	MZF1.01	0.68		M		H	transcription GO
regulation_of_transcription_DNA_dependent (1066)	EGR1.01	0.67	H	H	H		transcription GO
regulation_of_transcription_DNA_dependent (1066)	MTF-1.01	0.65	0		H	0	transcription GO
regulation_of_transcription_DNA_dependent (1066)	MAZ.01	0.65					transcription GO
regulation_of_transcription_DNA_dependent (1066)	ZIC2_01	0.65	H		H		transcription GO
regulation_of_transcription_DNA_dependent (1066)	ATF6.01	0.64	0		H	H	transcription GO
regulation_of_transcription_DNA_dependent (1066)	PLAG1_01	0.63					transcription GO
regulation_of_transcription_DNA_dependent (1066)	AHRARNT.0	0.62	0	H	H		transcription GO
regulation_of_transcription_DNA_dependent (1066)	HES1.01	0.62	H			H	transcription GO
regulation_of_transcription_DNA_dependent (1066)	MUSCLE_INI	0.62	0		H		transcription GO
regulation_of_transcription_DNA_dependent (1066)	GABP.01	0.62	0	M	H	H	transcription GO
regulation_of_transcription_DNA_dependent (1066)	NMYC.01	0.62	0		H	0	transcription GO
regulation_of_transcription_DNA_dependent (1066)	MUSCLE_INI	0.61	0		H		transcription GO
regulation_of_transcription_DNA_dependent (1066)	RREB1.01	0.6	H	H	H	M	transcription GO
regulation_of_transcription_DNA_dependent (1066)	CDE.01	0.6	H				transcription GO
regulation_of_transcription_DNA_dependent (1066)	AHR.01	0.59	0	H	H	M	transcription GO
regulation_of_transcription_DNA_dependent (1066)	E2F.02	0.59		H			transcription GO
regulation_of_transcription_DNA_dependent (1066)	WHN.01	0.59	0	H	0		transcription GO
regulation_of_transcription_DNA_dependent (1066)	TAXCREB.0	0.58	0	H	M	M	transcription GO
regulation_of_transcription_DNA_dependent (1066)	PAX9.01	0.57	0		H	0	transcription GO
regulation_of_transcription_DNA_dependent (1066)	PAX5.01	0.57	0	H		H	transcription GO
regulation_of_transcription_DNA_dependent (1066)	PAX5.03	0.56	0		0	0	transcription GO
regulation_of_transcription_DNA_dependent (1066)	VDR_RXR.0	0.56	H	0	M	0	transcription GO
regulation_of_transcription_DNA_dependent (1066)	GAGA.01	0.56				H	transcription GO
regulation_of_transcription_DNA_dependent (1066)	TAXCREB.0	0.56	0	0	M	H	transcription GO
regulation_of_transcription_DNA_dependent (1066)	E2F.03	0.54	H	H	H		transcription GO
regulation_of_transcription_DNA_dependent (1066)	NFY.01	0.52	0			0	transcription GO
regulation_of_transcription_DNA_dependent (1066)	AG_rich_cod	0.5				0	transcription GO
regulation_of_transcription_DNA_dependent (1066)	GKLF.01	0.49		H	0	0	transcription GO
regulation_of_transcription_DNA_dependent (1066)	CAAT.01	0.49	0		0	0	transcription GO
regulation_of_transcription_DNA_dependent (1066)	NFY.02	0.48	0		0	0	transcription GO
regulation_of_transcription (266)	GC_rich	0.99	H		H		transcription GO
regulation_of_transcription (266)	poly_C	0.99					transcription GO
regulation_of_transcription (266)	SP1.01	0.78	H		M	M	transcription GO
regulation_of_transcription (266)	WT1.01	0.77	H		H		transcription GO
regulation_of_transcription (266)	MAZR.01	0.76	H				transcription GO
regulation_of_transcription (266)	ZNF202.01	0.73				M	transcription GO
regulation_of_transcription (266)	AP2.01	0.73	0			H	transcription GO
regulation_of_transcription (266)	ZF5.01	0.72	0		H	H	transcription GO
regulation_of_transcription (266)	ZF9.01	0.72	0		H	0	transcription GO
regulation_of_transcription (266)	EGR3.01	0.71	H	M	H	M	transcription GO
regulation_of_transcription (266)	ZBP89.01	0.7				H	transcription GO
regulation_of_transcription (266)	NGFIC.01	0.69	H	M	H	M	transcription GO
regulation_of_transcription (266)	CKROX_01	0.68					transcription GO
regulation_of_transcription (266)	EGR1.02	0.68	H			H	transcription GO
regulation_of_transcription (266)	EGR2.01	0.68	H	0	0	M	transcription GO

regulation_of_transcription (266)	MTF-1.01	0.65	0		0	0	transcription GO
regulation_of_transcription (266)	MAZ.01	0.65				H	transcription GO
regulation_of_transcription (266)	PLAG1_01	0.63				H	transcription GO
regulation_of_transcription (266)	HES1.01	0.62	0		0	H	transcription GO
regulation_of_transcription (266)	CDE.01	0.6	H		H		transcription GO
regulation_of_transcription (266)	E2F.02	0.59	0	0		H	transcription GO
regulation_of_transcription (266)	TAXCREB.01	0.58	0	H	M	M	transcription GO
regulation_of_transcription (266)	VDR_RXR.02	0.56	H	H		0	transcription GO
regulation_of_transcription (266)	GAGA.01	0.56		H	M	H	transcription GO
regulation_of_transcription (266)	AG_rich_codi	0.5			M	H	transcription GO
regulation_of_transcription (266)	GKLF.01	0.49		H	0	0	transcription GO
regulation_of_transcription (266)	CAAT.01	0.49	0		0	0	transcription GO
regulation_of_transcription (266)	PBX1_MEIS1	0.44	0		0	0	transcription GO
development (530)	GC_rich	0.99	H				transcription GO
development (530)	poly_C	0.99			H		transcription GO
development (530)	SP1.01	0.78	0	H	M		transcription GO
development (530)	WT1.01	0.77	H				transcription GO
development (530)	HES1.02	0.76	0	0		0	transcription GO
development (530)	MAZR.01	0.76	H			H	transcription GO
development (530)	ZNF202.01	0.73					transcription GO
development (530)	AP2.01	0.73	H				transcription GO
development (530)	ZF9.01	0.72	0	0	H		transcription GO
development (530)	NFKAPPAB5	0.7	0	H	M	H	transcription GO
development (530)	HAND2_E12	0.7	0	M	H	0	transcription GO
development (530)	ZBP89.01	0.7	H				transcription GO
development (530)	NGFIC.01	0.69	0		0	0	transcription GO
development (530)	CKROX_01	0.68	0				transcription GO
development (530)	EGR1.02	0.68	0		H	H	transcription GO
development (530)	HIC1_01	0.68	0	H	H		transcription GO
development (530)	EGR2.01	0.68	0	M	0	H	transcription GO
development (530)	MZF1.01	0.68	H	H	H		transcription GO
development (530)	EGR1.01	0.67	0	M	H	0	transcription GO
development (530)	MAZ.01	0.65	H	H			transcription GO
development (530)	ZIC2_01	0.65	0	H	0		transcription GO
development (530)	PLAG1_01	0.63					transcription GO
development (530)	INSM1_01	0.63	0	0	H		transcription GO
development (530)	EBVR.01	0.63	0	H		0	transcription GO
development (530)	MUSCLE_INI	0.62	0		H	H	transcription GO
development (530)	CDE.01	0.6	0	0	H		transcription GO
development (530)	E2F.02	0.59	0	0	H		transcription GO
development (530)	PAX5.01	0.57	0	0	H	M	transcription GO
development (530)	GAGA.01	0.56		H		H	transcription GO
development (530)	AG_rich_codi	0.5		H	0	H	transcription GO
development (530)	GKLF.01	0.49	M	H	0	M	transcription GO
development (530)	MTATA.01	0.47		0	0	0	transcription GO
development (530)	AMEF2.01	0.38		0	0	0	transcription GO
Wnt_receptor_signaling_pathway (66)	GC_rich	0.99			H	H	general GO
Wnt_receptor_signaling_pathway (66)	WT1.01	0.77	0	H	M	0	general GO
Wnt_receptor_signaling_pathway (66)	ZNF202.01	0.73	M	H	0	H	general GO
Wnt_receptor_signaling_pathway (66)	ZF5.01	0.72	M	0	H	0	general GO
Wnt_receptor_signaling_pathway (66)	NRF1_01	0.71	H	0	M	H	general GO
Wnt_receptor_signaling_pathway (66)	NGFIC.01	0.69	H		0	H	general GO
Wnt_receptor_signaling_pathway (66)	EGR2.01	0.68	0	0	M	H	general GO
Wnt_receptor_signaling_pathway (66)	EGR1.01	0.67	H	M	0	H	general GO
Wnt_receptor_signaling_pathway (66)	CDE.01	0.6	H	M	H	0	general GO
Wnt_receptor_signaling_pathway (66)	PAX5.01	0.57	0		0	M	general GO
visual_perception (142)	HOX_PBX_0	0.48		0	0	0	general GO
visual_perception (142)	PTX1.01	0.45		H	0	0	general GO
visual_perception (142)	CRX.01	0.39		0	0	0	general GO
visual_perception (142)	OTX2.01	0.39		H	0	0	general GO
visual_perception (142)	CDX2.01	0.38	M	H	0	0	general GO
visual_perception (142)	SIX3.01	0.35		H	H	0	general GO
visual_perception (142)	EVI1.04	0.33	M	H	0	0	general GO
ubiquitin_dependent_protein_catabolism (72)	WHN.01	0.59		0	0	0	general GO

ubiquitin_cycle (154)	GC_rich	0.99		0	0	0	general GO
ubiquitin_cycle (154)	HES1.01	0.62		0	0	0	general GO
ubiquitin_cycle (154)	MUSCLE_INI	0.62		0	0	0	general GO
ubiquitin_cycle (154)	CDE.01	0.6		H	H	0	general GO
ubiquitin_cycle (154)	FLI.01	0.53		H	0	0	general GO
transport (940)	XBP1.01	0.47	H	M	0	0	general GO
transport (940)	OTX2.01	0.39	M	0	0	H	general GO
transport (940)	HNF1.03	0.31		0	0	0	general GO
transport (940)	HNF1.01	0.3		0	0	0	general GO
transmembrane_receptor_tyrosine_kinase_signaling	GAGA.01	0.56	H	M	0	0	general GO
transcription_RNA_polyII_promoter (190)	GC_rich	0.99	H		0	0	general GO
transcription_RNA_polyII_promoter (190)	poly_C	0.99	0		0	0	general GO
transcription_RNA_polyII_promoter (190)	SP1.01	0.78	0		0	0	general GO
transcription_RNA_polyII_promoter (190)	WT1.01	0.77	0		0	0	general GO
transcription_RNA_polyII_promoter (190)	ZNF202.01	0.73	0		0	0	general GO
transcription_RNA_polyII_promoter (190)	AP2.01	0.73	0	H		H	general GO
transcription_RNA_polyII_promoter (190)	EGR3.01	0.71	0	M	H	0	general GO
transcription_RNA_polyII_promoter (190)	NRF1_01	0.71	0		0	0	general GO
transcription_RNA_polyII_promoter (190)	HIC1_01	0.68	0	0	0		general GO
transcription_RNA_polyII_promoter (190)	MTF-1.01	0.65	0		0	0	general GO
transcription_RNA_polyII_promoter (190)	PLAG1_01	0.63	M	H	0	H	general GO
transcription_RNA_polyII_promoter (190)	CDE.01	0.6	0	H	M		general GO
synaptic_transmission (153)	NRSE.01	0.57	H		0	0	general GO
synaptic_transmission (153)	GAGA.01	0.56	0	0	M	H	general GO
synaptic_transmission (153)	ISL1.01	0.43	H	M	0	0	general GO
steroid_biosynthesis (42)	NFY.01	0.52	M	H	0	0	general GO
steroid_biosynthesis (42)	CAAT.01	0.49	M	H	0	0	general GO
steroid_biosynthesis (42)	NFY.03	0.47	M		0	0	general GO
sodium_ion_transport (75)	FXRE.01	0.51	0	M	H	0	general GO
sodium_ion_transport (75)	HNF1.03	0.31	H	M	0	0	general GO
sodium_ion_transport (75)	HNF1.01	0.3	H	M	0	0	general GO
small_GTPase_mediated_signal_transduction (124)	GC_rich	0.99		H	0	0	general GO
small_GTPase_mediated_signal_transduction (124)	SP1.01	0.78		H	H	0	general GO
small_GTPase_mediated_signal_transduction (124)	WT1.01	0.77		H	H	0	general GO
small_GTPase_mediated_signal_transduction (124)	ZF5.01	0.72		H	0	H	general GO
small_GTPase_mediated_signal_transduction (124)	ZF9.01	0.72		H	H	0	general GO
small_GTPase_mediated_signal_transduction (124)	EGR3.01	0.71	H	0	H	M	general GO
small_GTPase_mediated_signal_transduction (124)	NRF1_01	0.71		H	0	0	general GO
small_GTPase_mediated_signal_transduction (124)	NGFIC.01	0.69		0	0	0	general GO
small_GTPase_mediated_signal_transduction (124)	EGR1.02	0.68		H	0	0	general GO
small_GTPase_mediated_signal_transduction (124)	EGR2.01	0.68	H	0	0	M	general GO
small_GTPase_mediated_signal_transduction (124)	GC.01	0.68		H	0	0	general GO
small_GTPase_mediated_signal_transduction (124)	PLAG1_01	0.63		0	0	0	general GO
skeletal_development (83)	GAGA.01	0.56	H		H	0	general GO
signal_transduction (1189)	NFKAPPAB.(	0.57		0	0	0	general GO
signal_transduction (1189)	HIVEP1_01	0.52	H	M	0	0	general GO
signal_transduction (1189)	MEL1_02	0.46		0	0	0	general GO
signal_transduction (1189)	AARE.01	0.39		0	0	0	general GO
signal_transduction (1189)	EVI1.02	0.33		0	0	0	general GO
signal_transduction (1189)	EVI1.01	0.31		0	0	0	general GO
signal_transduction (1189)	poly_A	0.01		0	0	0	general GO
sensory_perception_of_smell (40)	NFE2L2.01	0.47	M	H	0	0	general GO
sensory_perception_of_smell (40)	LHX3.01	0.14	H		H	H	general GO
sensory_perception (201)	NFKAPPAB.(	0.57	H	M	0	0	general GO
sensory_perception (201)	IK2.01	0.49		0	0	0	general GO
sensory_perception (201)	SRF.03	0.47		H	0	0	general GO
sensory_perception (201)	MIT.01	0.46		H	H	0	general GO
sensory_perception (201)	MEL1_03	0.46			0	0	general GO
sensory_perception (201)	DBP.01	0.45		H	H	0	general GO
sensory_perception (201)	GATA1.01	0.45		0	0	0	general GO
sensory_perception (201)	PBX1_MEIS1	0.44	0	0	M	H	general GO
sensory_perception (201)	TGIF.01	0.44		0	0	0	general GO
sensory_perception (201)	SRF.01	0.42		H	0	0	general GO
sensory_perception (201)	RORA1.01	0.41		H	0	0	general GO

sensory_perception (201)	GFI1.01	0.41		H	0	0	general GO
sensory_perception (201)	CRX.01	0.39		H	0	0	general GO
sensory_perception (201)	AARE.01	0.39	H	H	M	0	general GO
sensory_perception (201)	AIRE.01	0.39		H	H	0	general GO
sensory_perception (201)	LTATA_01	0.39		H	0	0	general GO
sensory_perception (201)	OTX2.01	0.39		H	0	0	general GO
sensory_perception (201)	GATA1.03	0.39		H	H	0	general GO
sensory_perception (201)	NFAT.01	0.38		0	0	0	general GO
sensory_perception (201)	CDX2.01	0.38		H	H	0	general GO
sensory_perception (201)	GATA2.02	0.36		H	H	0	general GO
sensory_perception (201)	XFD3.01	0.36		0	0	H	general GO
sensory_perception (201)	OCT1P.01	0.36			H	0	general GO
sensory_perception (201)	SIX3.01	0.35		H	0	0	general GO
sensory_perception (201)	PDX1_G_SA	0.35		H	H	H	general GO
sensory_perception (201)	GATA2.01	0.35		H	H	0	general GO
sensory_perception (201)	NKX25.02	0.35		H	0	0	general GO
sensory_perception (201)	FREAC2.01	0.35	0	M	H	0	general GO
sensory_perception (201)	GATA3.01	0.34		H	H	0	general GO
sensory_perception (201)	XFD2.01	0.32	H		H	H	general GO
sensory_perception (201)	HNF1.03	0.31		H	0	0	general GO
sensory_perception (201)	PBX1.01	0.31	H		H	0	general GO
sensory_perception (201)	XVENT2.01	0.3		H	0	H	general GO
sensory_perception (201)	MEF2.02	0.3		H	0	0	general GO
sensory_perception (201)	MEL1_01	0.29		H	H	0	general GO
sensory_perception (201)	GATA3.02	0.29		H	H	0	general GO
sensory_perception (201)	EVI1.05	0.29		H	H	0	general GO
sensory_perception (201)	TATA.01	0.28		H	0	0	general GO
sensory_perception (201)	TATA.02	0.28		H	0	0	general GO
sensory_perception (201)	HFH8.01	0.27	H	H		0	general GO
sensory_perception (201)	OCT1.01	0.26	H	H		0	general GO
sensory_perception (201)	HNF3B.01	0.26		H	H	0	general GO
sensory_perception (201)	HFH2.01	0.25	H	H		0	general GO
sensory_perception (201)	EVI1.03	0.24		H	H	0	general GO
response_to_virus (55)	ISRE.01	0.43			0	0	general GO
response_to_virus (55)	IRF2.01	0.43			0	0	general GO
response_to_virus (55)	IRF3.01	0.42			0	0	general GO
response_to_virus (55)	PRDM1.01	0.41	0		0	0	general GO
response_to_virus (55)	HBP1_01	0.41	M	H	0	0	general GO
response_to_virus (55)	IRF1.01	0.4			H	0	general GO
response_to_virus (55)	BRN2.01	0.39		0	H	H	general GO
response_to_virus (55)	STAT5.01	0.39	H	0	M	0	general GO
response_to_virus (55)	IRF7.01	0.36		H	0	0	general GO
response_to_virus (55)	TATA.01	0.28		H	H	0	general GO
response_to_unfolded_protein (40)	STAT1.01	0.52	0		0	0	general GO
response_to_DNA_damage_stimulus (117)	E2F.02	0.59		M	0	0	general GO
response_to_DNA_damage_stimulus (117)	E2F.03	0.54	H	M	0	M	general GO
response_to_DNA_damage_stimulus (117)	FLI.01	0.53		0	0	0	general GO
response_to_DNA_damage_stimulus (117)	E2F.01	0.5	H	M	0	0	general GO
regulation_of_transcription_from_RNA_polymerase_	WT1.01	0.77	H		0	0	general GO
regulation_of_transcription_from_RNA_polymerase_	ZF5.01	0.72	H	H	0	M	general GO
regulation_of_transcription_from_RNA_polymerase_	MAZ.01	0.65		H	M	0	general GO
regulation_of_transcription_from_RNA_polymerase_	PLAG1_01	0.63	0		0	0	general GO
regulation_of_transcription_from_RNA_polymerase_	HES1.01	0.62	H	0	0	M	general GO
regulation_of_progression_through_cell_cycle (217)	GC_rich	0.99	H	M	0	0	general GO
regulation_of_progression_through_cell_cycle (217)	ZF5.01	0.72	H	M	H	0	general GO
regulation_of_progression_through_cell_cycle (217)	CREB.03	0.54	H	M	0	0	general GO
regulation_of_cyclin_dependent_protein_kinase_acti	ZF9.01	0.72	0	0		0	general GO
regulation_of_cyclin_dependent_protein_kinase_acti	NFY.01	0.52	0	H	M	0	general GO
regulation_of_apoptosis (59)	IRF3.01	0.42	M	H	0	0	general GO
proteolysis (297)	NRL.01	0.51	0	0	H	M	general GO
proteolysis (297)	AREB6.01	0.49		0	0	H	general GO
proteolysis (297)	AREB6.02	0.48		0	0	0	general GO
proteolysis (297)	AP1.01	0.43		0	0	0	general GO
proteolysis (297)	IRF4.01	0.39		H	0	0	general GO

proteolysis (297)	GATA1.03	0.39	0	H	0	M	general GO
proteolysis (297)	HNF1.02	0.34		0	0	0	general GO
proteolysis (297)	ATBF1.01	0.34		0	0	0	general GO
proteolysis (297)	HNF1.03	0.31		0	0	0	general GO
proteolysis (297)	HNF1.01	0.3		0	0	0	general GO
proteolysis (297)	HFH1.01	0.27		0	0	0	general GO
proteolysis (297)	MYT1.01	0.27		0	0	0	general GO
protein_transport (254)	GC_rich	0.99		0	0	0	general GO
protein_transport (254)	ZF5.01	0.72		H	0	0	general GO
protein_transport (254)	EGR3.01	0.71		0	0	0	general GO
protein_transport (254)	NRF1_01	0.71		H	0	0	general GO
protein_transport (254)	EGR1.02	0.68		0	0	0	general GO
protein_transport (254)	AHRARNT.02	0.62	M	H	0	0	general GO
protein_transport (254)	MUSCLE_INI	0.62		0	0	0	general GO
protein_transport (254)	GABP.01	0.62		0	0	0	general GO
protein_transport (254)	NRF2.01	0.57		0	0	0	general GO
protein_transport (254)	CREB.02	0.56		0	0	0	general GO
protein_transport (254)	ATF.01	0.55	H	M	0	0	general GO
protein_targeting (37)	NRF2.01	0.57		0	0	0	general GO
protein_targeting (37)	FLI.01	0.53		0	0	0	general GO
protein_folding (149)	ELK1.02	0.55	H	M	0	0	general GO
protein_folding (149)	FLI.01	0.53	H		H	0	general GO
protein_complex_assembly (94)	P53.01	0.61	0	0	0		general GO
protein_biosynthesis (197)	GABP.01	0.62		0	0	0	general GO
protein_biosynthesis (197)	CDE.01	0.6		H	H	H	general GO
protein_biosynthesis (197)	WHN.01	0.59	H		H	0	general GO
protein_biosynthesis (197)	NRF2.01	0.57		M	0	0	general GO
protein_biosynthesis (197)	ATF.01	0.55	H	M	0	0	general GO
protein_biosynthesis (197)	CETS1P54.0	0.55		M	0	0	general GO
protein_biosynthesis (197)	YY1.01	0.55		H	0	0	general GO
protein_biosynthesis (197)	ELK1.02	0.55		H	0	0	general GO
protein_biosynthesis (197)	NUDR.01	0.54	H		0	0	general GO
protein_biosynthesis (197)	E2F.03	0.54	H		H	0	general GO
protein_biosynthesis (197)	FLI.01	0.53		H	0	0	general GO
protein_biosynthesis (197)	ELK1.01	0.52		0	0	0	general GO
protein_biosynthesis (197)	E2F.01	0.5		0	0	0	general GO
protein_biosynthesis (197)	ISRE.01	0.43		0	0	0	general GO
protein_amino_acid_phosphorylation (373)	SP1.01	0.78	H	H		0	general GO
protein_amino_acid_phosphorylation (373)	ZF9.01	0.72	H	H		H	general GO
protein_amino_acid_phosphorylation (373)	PAX9.01	0.57	0		0	0	general GO
protein_amino_acid_dephosphorylation (96)	MUSCLE_INI	0.61	M	0	H	0	general GO
pregnancy (41)	XVENT2.01	0.3	H	0	0	M	general GO
pregnancy (41)	CDP.02	0.29	M	0	0	H	general GO
potassium_ion_transport (108)	poly_C	0.99	H	H	H		general GO
potassium_ion_transport (108)	SP1.01	0.78	0	H	M	0	general GO
potassium_ion_transport (108)	NGFIC.01	0.69	0	M	0	H	general GO
potassium_ion_transport (108)	EGR2.01	0.68	0	0	0		general GO
potassium_ion_transport (108)	INSM1_01	0.63	0	M	0	H	general GO
positive_regulation_of_I_kappaB_kinase_NF_kappa	ETS1.01	0.52	H	0	0	M	general GO
positive_regulation_of_I_kappaB_kinase_NF_kappa	IRF3.01	0.42		0	0	0	general GO
positive_regulation_of_cell_proliferation (103)	HEN1.02	0.66	0	M	H	0	general GO
organ_morphogenesis (71)	GAGA.01	0.56	0	H	M	0	general GO
nucleosome_assembly (71)	NFY.02	0.48	H		H	0	general GO
nucleosome_assembly (71)	TATA.01	0.28		H	0	0	general GO
nuclear_mRNA_splicing_via_spliceosome (94)	NRF2.01	0.57		H	0	0	general GO
nervous_system_development (225)	ZNF202.01	0.73	H	H	H		general GO
nervous_system_development (225)	GAGA.01	0.56		H	H	H	general GO
nervous_system_development (225)	AG_rich_cod	0.5		H	0	0	general GO
muscle_development (107)	INSM1_01	0.63		0	0	0	general GO
muscle_development (107)	SRF.02	0.49	H		0	0	general GO
muscle_development (107)	MTATA.01	0.47		0	0	0	general GO
muscle_development (107)	SRF.03	0.47	H		0	0	general GO
muscle_development (107)	AMEF2.01	0.38	M	H	0	0	general GO
muscle_development (107)	MEF2.02	0.3	H		0	0	general GO

muscle_development (107)	RSRFC4.01	0.28		H	0	0	general GO
mRNA_processing (148)	NRF1_01	0.71	H	M	0	0	general GO
mRNA_processing (148)	E2F.02	0.59		H	0	0	general GO
morphogenesis (104)	WT1.01	0.77	0	H	H		general GO
morphogenesis (104)	AP2.01	0.73	0	H	0		general GO
morphogenesis (104)	EGR1.02	0.68	H	0	M	H	general GO
morphogenesis (104)	MUSCLE_INI	0.61	0	H	H	M	general GO
mitosis (86)	CDE.01	0.6	M	H	0	0	general GO
mitosis (86)	WHN.01	0.59	0		0	0	general GO
mitosis (86)	NFY.01	0.52			0	0	general GO
mitosis (86)	CAAT.01	0.49			0	0	general GO
mitosis (86)	NFY.02	0.48		0	0	0	general GO
mitosis (86)	CHR.01	0.41		0	0	0	general GO
metabolism (241)	NFY.02	0.48		0	0	0	general GO
metabolism (241)	E4BP4.01	0.34	M	H	0	0	general GO
metabolism (241)	GATA3.02	0.29	0	M	0	H	general GO
lipid_transport (41)	SRF.03	0.47		0	0	0	general GO
lipid_transport (41)	RORA1.01	0.41		0	0	0	general GO
lipid_catabolism (41)	RORA1.01	0.41	0	0	H	M	general GO
lipid_biosynthesis (54)	CAAT.01	0.49	M	H	0	0	general GO
lipid_biosynthesis (54)	NFY.02	0.48	M	H	0	0	general GO
lipid_biosynthesis (54)	NFY.03	0.47	M	H	0	0	general GO
ion_transport (329)	NRSE.01	0.57	M	0	0	H	general GO
intracellular_protein_transport (191)	GC_rich	0.99		H	0	0	general GO
intracellular_protein_transport (191)	NRF1_01	0.71		H	0	0	general GO
intracellular_protein_transport (191)	ATF6.02	0.6		0	0	0	general GO
intracellular_protein_transport (191)	CDE.01	0.6		H	0	0	general GO
intracellular_protein_transport (191)	AHR.01	0.59	M	H	0	0	general GO
intracellular_protein_transport (191)	ATF.01	0.55	H	M	0	0	general GO
intracellular_protein_transport (191)	XBP1.01	0.47		0	0	H	general GO
innate_immune_response (46)	PAX2.01	0.47		0	0	0	general GO
innate_immune_response (46)	TST1.01	0.43		0	0	0	general GO
innate_immune_response (46)	ISL1.01	0.43	H	M	0	0	general GO
innate_immune_response (46)	IRF2.01	0.43		0	0	0	general GO
innate_immune_response (46)	IRF3.01	0.42		0	0	0	general GO
innate_immune_response (46)	PSE_02	0.41		H	H	0	general GO
innate_immune_response (46)	BRN4.01	0.4		H	0	0	general GO
innate_immune_response (46)	CEBPB.01	0.4		H	H	0	general GO
innate_immune_response (46)	OCT1.04	0.38	M	0	H	0	general GO
innate_immune_response (46)	CDX2.01	0.38		0	0	H	general GO
innate_immune_response (46)	IRF7.01	0.36		0	0	0	general GO
innate_immune_response (46)	OCT.01	0.35		0	H	0	general GO
innate_immune_response (46)	OCT1.02	0.34		0	0	0	general GO
innate_immune_response (46)	XFD1.01	0.34		H	0	0	general GO
innate_immune_response (46)	ATBF1.01	0.34		0	0	0	general GO
innate_immune_response (46)	XFD2.01	0.32		H	H	0	general GO
innate_immune_response (46)	LMX1B.01	0.32		H	0	0	general GO
innate_immune_response (46)	EN1.01	0.32		H	0	0	general GO
innate_immune_response (46)	BRN3.01	0.3		0	0	0	general GO
innate_immune_response (46)	MEF2.03	0.3	M	H	0	0	general GO
innate_immune_response (46)	HNF1.01	0.3		0	0	0	general GO
innate_immune_response (46)	GATA3.02	0.29	0	M	H	0	general GO
innate_immune_response (46)	PIT1.01	0.27		0		0	general GO
innate_immune_response (46)	SATB1.01	0.27		0	0	H	general GO
innate_immune_response (46)	HFH8.01	0.27	M	H	0	0	general GO
innate_immune_response (46)	OCT1.01	0.26		0	0	0	general GO
innate_immune_response (46)	NKX31.01	0.25	M	0	0	H	general GO
innate_immune_response (46)	OCT1.06	0.22		0	0	0	general GO
inflammatory_response (163)	NFKAPPAB.	0.6			0	0	general GO
inflammatory_response (163)	NFKAPPAB.	0.57	H		0	0	general GO
inflammatory_response (163)	NFKAPPAB6	0.55		M	0	0	general GO
inflammatory_response (163)	CREL.01	0.55		M	0	0	general GO
inflammatory_response (163)	NFKAPPAB.	0.55			0	0	general GO
inflammatory_response (163)	HIVEP1_01	0.52			0	0	general GO

inflammatory_response (163)	TH1E47.01	0.51		0	0	0	general GO
inflammatory_response (163)	ETS2.01	0.48	H		0	0	general GO
inflammatory_response (163)	GRE.01	0.47		H	0	0	general GO
inflammatory_response (163)	NFE2L2.01	0.47		0	0	0	general GO
inflammatory_response (163)	RP58.01	0.46		0	0	0	general GO
inflammatory_response (163)	CEBP.02	0.46	M		0	0	general GO
inflammatory_response (163)	COMP1.01	0.45		H	0	0	general GO
inflammatory_response (163)	BARBIE.01	0.45		0	0	0	general GO
inflammatory_response (163)	GATA1.01	0.45		M	0	0	general GO
inflammatory_response (163)	MYT1L.01	0.44		0	0	0	general GO
inflammatory_response (163)	TCF11MAFG	0.43		0	H	0	general GO
inflammatory_response (163)	AP1.01	0.43		H	0	0	general GO
inflammatory_response (163)	ISL1.01	0.43	M	H	H	0	general GO
inflammatory_response (163)	ISRE.01	0.43		0	0	0	general GO
inflammatory_response (163)	IRF3.01	0.42		0	0	0	general GO
inflammatory_response (163)	SRF.01	0.42		0	0	0	general GO
inflammatory_response (163)	PRDM1.01	0.41		H	0	0	general GO
inflammatory_response (163)	HMGIY.01	0.41		H	H	0	general GO
inflammatory_response (163)	PDX1_Gabi	0.41	M	0	H	0	general GO
inflammatory_response (163)	GATA.01	0.41	M	H	0	0	general GO
inflammatory_response (163)	HBP1_01	0.41	H	H	0	M	general GO
inflammatory_response (163)	PSE_02	0.41	H	M	0	0	general GO
inflammatory_response (163)	BRN4.01	0.4		H	0	0	general GO
inflammatory_response (163)	IRF1.01	0.4		M	0	0	general GO
inflammatory_response (163)	CEBPB.01	0.4		H	0	0	general GO
inflammatory_response (163)	AARE.01	0.39		0	0	0	general GO
inflammatory_response (163)	AIRE.01	0.39		H	0	0	general GO
inflammatory_response (163)	LTATA_01	0.39		H	0	0	general GO
inflammatory_response (163)	BRN2.01	0.39		M	0	0	general GO
inflammatory_response (163)	IRF4.01	0.39			0	0	general GO
inflammatory_response (163)	STAT5.01	0.39		H	0	0	general GO
inflammatory_response (163)	GATA1.03	0.39	M		H	0	general GO
inflammatory_response (163)	BCL6.01	0.39		H	M	0	general GO
inflammatory_response (163)	AMEF2.01	0.38		H	0	0	general GO
inflammatory_response (163)	NFAT.01	0.38			0	0	general GO
inflammatory_response (163)	CDX2.01	0.38	H		H	0	general GO
inflammatory_response (163)	MMEF2.01	0.37		H	0	0	general GO
inflammatory_response (163)	SOX9.01	0.37	0	0		0	general GO
inflammatory_response (163)	XFD3.01	0.36		0	0	0	general GO
inflammatory_response (163)	IRF7.01	0.36		H	0	0	general GO
inflammatory_response (163)	OCT1P.01	0.36	H		0	0	general GO
inflammatory_response (163)	CDX1.01	0.35		H	0	0	general GO
inflammatory_response (163)	DLX3.01	0.34	0		0	0	general GO
inflammatory_response (163)	HOXC13_01	0.34	M	H	H	0	general GO
inflammatory_response (163)	HNF1.02	0.34		0	0	0	general GO
inflammatory_response (163)	XFD1.01	0.34		H	0	0	general GO
inflammatory_response (163)	ATBF1.01	0.34	M	H	H	0	general GO
inflammatory_response (163)	GATA3.01	0.34		H	H	0	general GO
inflammatory_response (163)	EVI1.04	0.33		M	0	0	general GO
inflammatory_response (163)	MEIS1_HOX/	0.33	H	M	H	0	general GO
inflammatory_response (163)	PBX_HOXA9	0.33	H	M	H	0	general GO
inflammatory_response (163)	BRN3.02	0.33		0	0	0	general GO
inflammatory_response (163)	BRN2.03	0.32	H		0	0	general GO
inflammatory_response (163)	XFD2.01	0.32			0	0	general GO
inflammatory_response (163)	OC2.01	0.32	0		0	0	general GO
inflammatory_response (163)	LMX1B.01	0.32		H	0	0	general GO
inflammatory_response (163)	HNF1.03	0.31		0	0	0	general GO
inflammatory_response (163)	EVI1.01	0.31		0	H	0	general GO
inflammatory_response (163)	HNF6.01	0.31	M	H	H	0	general GO
inflammatory_response (163)	BRN3.01	0.3		H	0	0	general GO
inflammatory_response (163)	ATATA.01	0.3		0	0	0	general GO
inflammatory_response (163)	MEF2.02	0.3		H	H	0	general GO
inflammatory_response (163)	MEF2.03	0.3		H	H	0	general GO
inflammatory_response (163)	HNF1.01	0.3		0	0	0	general GO

inflammatory_response (163)	MEF2.05	0.29		0	H	0	general GO
inflammatory_response (163)	RSRFC4.02	0.29			M	0	general GO
inflammatory_response (163)	MEL1_01	0.29	M		H	0	general GO
inflammatory_response (163)	EVI1.05	0.29	0		0	0	general GO
inflammatory_response (163)	TATA.01	0.28		0	0	0	general GO
inflammatory_response (163)	TATA.02	0.28		0	0	0	general GO
inflammatory_response (163)	MYT1.02	0.28		H	0	0	general GO
inflammatory_response (163)	PIT1.01	0.27		H	0	0	general GO
inflammatory_response (163)	FREAC7.01	0.27		H	0	0	general GO
inflammatory_response (163)	HFH1.01	0.27		H	0	0	general GO
inflammatory_response (163)	MYT1.01	0.27		H	0	0	general GO
inflammatory_response (163)	OCT1.01	0.26		H	M	0	general GO
inflammatory_response (163)	BRN5.01	0.26		0	0	0	general GO
inflammatory_response (163)	HNF3B.01	0.26		0	0	0	general GO
inflammatory_response (163)	BRN2.02	0.25	H		0	0	general GO
inflammatory_response (163)	EVI1.03	0.24	M		H	0	general GO
inflammatory_response (163)	OCT1.06	0.22	H	H		0	general GO
induction_of_apoptosis (86)	ISRE.01	0.43	H	0	0	M	general GO
immune_response (366)	NFKAPPAB.(	0.6		H	0	0	general GO
immune_response (366)	NFKAPPAB.(	0.57		H	0	0	general GO
immune_response (366)	NFKAPPAB6	0.55			H	0	general GO
immune_response (366)	CREL.01	0.55			H	0	general GO
immune_response (366)	NFKAPPAB.(	0.55		H	0	0	general GO
immune_response (366)	HIVEP1_01	0.52		H	0	0	general GO
immune_response (366)	AML1.01	0.52		H	H	0	general GO
immune_response (366)	ERR_01	0.52		H	0	0	general GO
immune_response (366)	ETS1.01	0.52		H	0	0	general GO
immune_response (366)	AML3.01	0.5		M	0	0	general GO
immune_response (366)	AP1.02	0.49	H		0	0	general GO
immune_response (366)	TR2.01	0.49			0	0	general GO
immune_response (366)	LXRE.01	0.48		H	0	0	general GO
immune_response (366)	ETS2.01	0.48		H	0	0	general GO
immune_response (366)	GRE.01	0.47		H	0	0	general GO
immune_response (366)	PAX2.01	0.47	M	H	0	0	general GO
immune_response (366)	AP1.03	0.47	H		0	0	general GO
immune_response (366)	PPARA.01	0.47	M	H	0	0	general GO
immune_response (366)	NFE2L2.01	0.47		H	0	0	general GO
immune_response (366)	MTATA.01	0.47		0	0	0	general GO
immune_response (366)	SRF.03	0.47	H	H		0	general GO
immune_response (366)	BACH1.01	0.47	H		H	0	general GO
immune_response (366)	PLZF.01	0.47		0	0	0	general GO
immune_response (366)	AREB6.04	0.47		H	0	0	general GO
immune_response (366)	GKLF_02	0.46		0	0	0	general GO
immune_response (366)	TAACC.01	0.46		H	0	0	general GO
immune_response (366)	MIT.01	0.46			H	H	general GO
immune_response (366)	RP58.01	0.46		H	M	0	general GO
immune_response (366)	PXRCAR.01	0.45	H		0	0	general GO
immune_response (366)	DBP.01	0.45	M	0	H	0	general GO
immune_response (366)	BARBIE.01	0.45		H	0	0	general GO
immune_response (366)	BRACH.01	0.45	H	0	M	0	general GO
immune_response (366)	PAX8.01	0.45		M	0	0	general GO
immune_response (366)	GATA1.01	0.45		0	H	0	general GO
immune_response (366)	RTR.01	0.45		H	H	0	general GO
immune_response (366)	STAT.01	0.44	H		0	0	general GO
immune_response (366)	PRE.01	0.44		H	0	0	general GO
immune_response (366)	TCF11.01	0.44	0		0	0	general GO
immune_response (366)	BCL6.02	0.44	H		0	0	general GO
immune_response (366)	TCF11MAFG	0.43		H	H	0	general GO
immune_response (366)	ILF1_01	0.43		H	0	0	general GO
immune_response (366)	AP1.01	0.43		H	H	0	general GO
immune_response (366)	ISL1.01	0.43		H	H	0	general GO
immune_response (366)	ISRE.01	0.43			0	0	general GO
immune_response (366)	IRF2.01	0.43			0	0	general GO
immune_response (366)	IRF3.01	0.42			0	0	general GO

immune_response (366)	SRF.01	0.42			H	0	general GO
immune_response (366)	NKX25.01	0.42		0	H	0	general GO
immune_response (366)	GATA1.02	0.41		H	0	0	general GO
immune_response (366)	PRDM1.01	0.41		H	M	0	general GO
immune_response (366)	HMGIY.01	0.41			H	0	general GO
immune_response (366)	PDX1_Gabi	0.41	M	H	H	0	general GO
immune_response (366)	FAST1.01	0.41		H	0	0	general GO
immune_response (366)	GATA.01	0.41		H	H	0	general GO
immune_response (366)	HBP1_01	0.41		0	0	0	general GO
immune_response (366)	RORA1.01	0.41		H	H	0	general GO
immune_response (366)	PHOX2_01	0.41	M	H	H	0	general GO
immune_response (366)	PSE_02	0.41		H	0	H	general GO
immune_response (366)	BRN4.01	0.4		H	0	0	general GO
immune_response (366)	LEF1.02	0.4		H	0	0	general GO
immune_response (366)	MTBF.01	0.4		M	H	0	general GO
immune_response (366)	IRF1.01	0.4			0	0	general GO
immune_response (366)	CEBPB.01	0.4		H	0	0	general GO
immune_response (366)	GATA1.04	0.4		H	0	0	general GO
immune_response (366)	CRX.01	0.39	M	H	0	0	general GO
immune_response (366)	OCT1.05	0.39		H	H	0	general GO
immune_response (366)	AARE.01	0.39			0	0	general GO
immune_response (366)	AIRE.01	0.39		H	0	H	general GO
immune_response (366)	LTATA_01	0.39		H	0	0	general GO
immune_response (366)	BRN2.01	0.39		H	H	0	general GO
immune_response (366)	IRF4.01	0.39			0	0	general GO
immune_response (366)	STAT5.01	0.39	H		0	0	general GO
immune_response (366)	GATA1.03	0.39		H	0	0	general GO
immune_response (366)	BCL6.01	0.39	H		0	0	general GO
immune_response (366)	OCT1.04	0.38		H	0	0	general GO
immune_response (366)	GFI1B.01	0.38	H		0	0	general GO
immune_response (366)	AMEF2.01	0.38			0	0	general GO
immune_response (366)	RORA2.01	0.38		H	H	0	general GO
immune_response (366)	NFAT.01	0.38			0	0	general GO
immune_response (366)	FREAC4.01	0.38	M	H	0	H	general GO
immune_response (366)	CDX2.01	0.38		H	0	0	general GO
immune_response (366)	MEF2.01	0.38		H	0	0	general GO
immune_response (366)	MMEF2.01	0.37		0	0	0	general GO
immune_response (366)	GATA2.02	0.36			M	H	general GO
immune_response (366)	GATA1.05	0.36			0	H	general GO
immune_response (366)	XFD3.01	0.36		H	H	0	general GO
immune_response (366)	IRF7.01	0.36			0	0	general GO
immune_response (366)	OCT1P.01	0.36	M	H	H	0	general GO
immune_response (366)	OCT.01	0.35		H	H	0	general GO
immune_response (366)	PDX1_G_SA	0.35		H	0	0	general GO
immune_response (366)	GATA2.01	0.35			0	0	general GO
immune_response (366)	CDX1.01	0.35		H	0	0	general GO
immune_response (366)	VBP.01	0.35	M	H	0	0	general GO
immune_response (366)	NKX25.02	0.35	M	H	0	0	general GO
immune_response (366)	OCT1.02	0.34		H	H	0	general GO
immune_response (366)	DLX3.01	0.34		H	0	0	general GO
immune_response (366)	HOXC13_01	0.34		H	0	0	general GO
immune_response (366)	XFD1.01	0.34		0	H	0	general GO
immune_response (366)	GSH2_01	0.34	M	H	0	0	general GO
immune_response (366)	ATBF1.01	0.34		H	H	0	general GO
immune_response (366)	HMEF2.01	0.34		H	0	0	general GO
immune_response (366)	GATA3.01	0.34			0	0	general GO
immune_response (366)	EVI1.04	0.33		H	H	0	general GO
immune_response (366)	EVI1.02	0.33		H	0	0	general GO
immune_response (366)	MEIS1_HOX/	0.33		H	H	H	general GO
immune_response (366)	PBX_HOXA9	0.33		H	H	H	general GO
immune_response (366)	BRN2.03	0.32	H		0	0	general GO
immune_response (366)	XFD2.01	0.32		H	0	0	general GO
immune_response (366)	OC2.01	0.32	H		0	0	general GO
immune_response (366)	LMX1B.01	0.32		H	H	0	general GO

immune_response (366)	FREAC3.01	0.32		H	0	0	general GO
immune_response (366)	CART1.01	0.32			0	0	general GO
immune_response (366)	HNF1.03	0.31		H	H	0	general GO
immune_response (366)	EVI1.01	0.31		0	H	0	general GO
immune_response (366)	SOX5.01	0.31		H	0	0	general GO
immune_response (366)	MSX.01	0.3	M	H	H	0	general GO
immune_response (366)	BRN3.01	0.3		H	0	0	general GO
immune_response (366)	ATATA.01	0.3		0	0	0	general GO
immune_response (366)	EVI1.06	0.3		H	H	0	general GO
immune_response (366)	MEF2.02	0.3		H	H	0	general GO
immune_response (366)	MEF2.03	0.3		H	H	0	general GO
immune_response (366)	HNF1.01	0.3		H	0	0	general GO
immune_response (366)	DLX1.01	0.29		H	0	0	general GO
immune_response (366)	MEF2.05	0.29		0	0	0	general GO
immune_response (366)	RSRFC4.02	0.29		H	H	0	general GO
immune_response (366)	MEL1_01	0.29			0	H	general GO
immune_response (366)	GATA3.02	0.29	H		H	H	general GO
immune_response (366)	MEF2.04	0.29		H	0	0	general GO
immune_response (366)	EVI1.05	0.29			0	0	general GO
immune_response (366)	TATA.01	0.28		H	0	0	general GO
immune_response (366)	RSRFC4.01	0.28			H	0	general GO
immune_response (366)	TATA.02	0.28		H	0	0	general GO
immune_response (366)	PIT1.01	0.27		H	H	0	general GO
immune_response (366)	FREAC7.01	0.27		H	0	0	general GO
immune_response (366)	HFH1.01	0.27		H	0	0	general GO
immune_response (366)	HFH8.01	0.27		H	0	0	general GO
immune_response (366)	MYT1.01	0.27		H	0	0	general GO
immune_response (366)	OCT1.01	0.26		H		0	general GO
immune_response (366)	BRN5.01	0.26		0	0	0	general GO
immune_response (366)	HNF3B.01	0.26		H	M	0	general GO
immune_response (366)	HFH2.01	0.25	M	H	0	0	general GO
immune_response (366)	NKX31.01	0.25		H	0	0	general GO
immune_response (366)	EVI1.03	0.24		H	0	H	general GO
immune_response (366)	S8.01	0.24	M	0	H	0	general GO
immune_response (366)	BRIGHT.01	0.22		H	H	0	general GO
immune_response (366)	OCT1.06	0.22		H	H	0	general GO
immune_response (366)	LHX3.01	0.14		H	H	0	general GO
immune_response (366)	poly_A	0.01		H	0	0	general GO
homophilic_cell_adhesion (70)	NFAT.01	0.38		0	0	0	general GO
homophilic_cell_adhesion (70)	OCT1P.01	0.36	0	M	H	0	general GO
G_protein_coupled_receptor_protein_signaling_path	TAL1BETAIT	0.47	H	M	0	0	general GO
G_protein_coupled_receptor_protein_signaling_path	MEL1_02	0.46		0	0	0	general GO
G_protein_coupled_receptor_protein_signaling_path	BRACH.01	0.45		H	0	0	general GO
G_protein_coupled_receptor_protein_signaling_path	TCF11MAFG	0.43		0	0	0	general GO
G_protein_coupled_receptor_protein_signaling_path	ISL1.01	0.43		0	0	0	general GO
G_protein_coupled_receptor_protein_signaling_path	GATA1.04	0.4		H	0	0	general GO
G_protein_coupled_receptor_protein_signaling_path	IRF4.01	0.39		0	0	0	general GO
G_protein_coupled_receptor_protein_signaling_path	RORA2.01	0.38		H	0	0	general GO
G_protein_coupled_receptor_protein_signaling_path	FREAC4.01	0.38		H	0	0	general GO
G_protein_coupled_receptor_protein_signaling_path	GATA2.02	0.36	H		0	0	general GO
G_protein_coupled_receptor_protein_signaling_path	XFD3.01	0.36		0	H	0	general GO
G_protein_coupled_receptor_protein_signaling_path	GATA2.01	0.35		H	H	0	general GO
G_protein_coupled_receptor_protein_signaling_path	GATA3.01	0.34		H	H	0	general GO
G_protein_coupled_receptor_protein_signaling_path	EVI1.02	0.33		0	0	0	general GO
G_protein_coupled_receptor_protein_signaling_path	XFD2.01	0.32		0	0	H	general GO
G_protein_coupled_receptor_protein_signaling_path	OC2.01	0.32		H	0	0	general GO
G_protein_coupled_receptor_protein_signaling_path	EVI1.01	0.31		H	H	0	general GO
G_protein_coupled_receptor_protein_signaling_path	MEL1_01	0.29		H	H	0	general GO
G_protein_coupled_receptor_protein_signaling_path	GATA3.02	0.29	H		H	0	general GO
G_protein_coupled_receptor_protein_signaling_path	EVI1.05	0.29		0	0	0	general GO
G_protein_coupled_receptor_protein_signaling_path	HFH1.01	0.27		0	H	0	general GO
G_protein_coupled_receptor_protein_signaling_path	EVI1.03	0.24		H	0	0	general GO
G_protein_coupled_receptor_protein_signaling_path	poly_A	0.01		0	0	0	general GO
G_protein_coupled_receptor_protein_signaling_path	AT_rich	0.01		H	0	0	general GO

fatty_acid_metabolism (55)	COUP.01	0.48	M	H	0	0	general GO
excretion (35)	NRL.01	0.51	H	0	M	0	general GO
ER_to_Golgi_transport (47)	ATF6.02	0.6		0	0	0	general GO
ER_to_Golgi_transport (47)	E4F.01	0.51		0	0	0	general GO
ER_to_Golgi_transport (47)	CREBP1CJU	0.44		0	0	0	general GO
epidermis_development (59)	AML3.01	0.5	M	0	H	0	general GO
epidermis_development (59)	TATA.01	0.28		0	0	0	general GO
epidermis_development (59)	TATA.02	0.28		0	0	0	general GO
electron_transport (214)	LMO2COM.0	0.48	0		0	0	general GO
electron_transport (214)	TAL1BETAIT	0.47	0	M	H	0	general GO
electron_transport (214)	PLZF.01	0.47	0		0	0	general GO
electron_transport (214)	GATA1.03	0.39	M	H	0	0	general GO
DNA_replication (91)	ELK1.02	0.55	H	M	0	0	general GO
DNA_replication (91)	E2F.03	0.54	H		0	0	general GO
DNA_repair (148)	E2F.02	0.59		M	0	0	general GO
DNA_repair (148)	E2F.03	0.54		M	0	0	general GO
DNA_repair (148)	FLI.01	0.53		H	0	0	general GO
DNA_repair (148)	NFY.01	0.52		0	0	0	general GO
DNA_repair (148)	E2F.01	0.5	H	M	0	0	general GO
DNA_metabolism (47)	E2F.02	0.59		0	0	0	general GO
DNA_metabolism (47)	E2F.03	0.54		0	0	0	general GO
DNA_metabolism (47)	E2F.01	0.5		0	0	0	general GO
digestion (51)	FREAC3.01	0.32		0	0	0	general GO
digestion (51)	MEL1_01	0.29	M	H	0	0	general GO
digestion (51)	TATA.01	0.28		0	0	0	general GO
digestion (51)	FREAC7.01	0.27		0	0	0	general GO
digestion (51)	HFH1.01	0.27		0	0	0	general GO
digestion (51)	HFH8.01	0.27		0	0	0	general GO
digestion (51)	HNF3B.01	0.26		0	0	0	general GO
digestion (51)	EVI1.03	0.24	M	H	0	0	general GO
defense_response (100)	NFKAPPAB.(	0.55	M	H	0	0	general GO
defense_response (100)	PAX8.01	0.45	0	0		0	general GO
defense_response (100)	TCF11.01	0.44	H	M	0	H	general GO
defense_response (100)	MTBF.01	0.4	M	H	0	0	general GO
defense_response (100)	NKX31.01	0.25	M	H	H	0	general GO
circulation (52)	SRF.01	0.42	0	0	H	M	general GO
chromosome_organization_and_biogenesis (83)	TATA.01	0.28		H	0	0	general GO
chromatin_modification (53)	GC_rich	0.99	0	0		0	general GO
chloride_transport (38)	ER.01	0.51	0	0		0	general GO
chemotaxis (98)	NFKAPPAB.(	0.6	H	M	0	0	general GO
chemotaxis (98)	NFKAPPAB.(	0.57	H	M	0	0	general GO
chemotaxis (98)	NFKAPPAB6	0.55	H	M	0	0	general GO
chemotaxis (98)	NFKAPPAB.(	0.55	H		0	0	general GO
chemotaxis (98)	HIVEP1_01	0.52	H	M	0	0	general GO
chemotaxis (98)	AP1.03	0.47	H	M	0	0	general GO
chemotaxis (98)	NFE2L2.01	0.47		0	0	0	general GO
chemotaxis (98)	GKLF_02	0.46		0	0	0	general GO
chemotaxis (98)	BARBIE.01	0.45		0	0	0	general GO
chemotaxis (98)	GATA1.01	0.45		0	0	0	general GO
chemotaxis (98)	TCF11MAFG	0.43		0	0	0	general GO
chemotaxis (98)	AP1.01	0.43		H	0	0	general GO
chemotaxis (98)	ISL1.01	0.43	M	0	H	0	general GO
chemotaxis (98)	SRF.01	0.42		H	0	0	general GO
chemotaxis (98)	GATA.01	0.41		0	0	0	general GO
chemotaxis (98)	PSE_02	0.41	H	M	0	0	general GO
chemotaxis (98)	GATA1.04	0.4		0	0	0	general GO
chemotaxis (98)	AARE.01	0.39	M	H	0	0	general GO
chemotaxis (98)	LTATA_01	0.39		0	0	0	general GO
chemotaxis (98)	BRN2.01	0.39		0	0	0	general GO
chemotaxis (98)	GATA1.03	0.39		0	H	0	general GO
chemotaxis (98)	BCL6.01	0.39	H	0	M	0	general GO
chemotaxis (98)	NFAT.01	0.38		0	0	0	general GO
chemotaxis (98)	CDX2.01	0.38		0	H	0	general GO
chemotaxis (98)	GATA1.05	0.36		0	0	H	general GO

chemotaxis (98)	PDX1_G_SA	0.35	M	0	H	0	general GO
chemotaxis (98)	GATA2.01	0.35		0	H	0	general GO
chemotaxis (98)	CDX1.01	0.35		0	0	0	general GO
chemotaxis (98)	GATA3.01	0.34		0	H	0	general GO
chemotaxis (98)	MEF2.05	0.29		0		0	general GO
chemotaxis (98)	TATA.01	0.28		0	0	0	general GO
chemotaxis (98)	TATA.02	0.28		0	H	0	general GO
central_nervous_system_development (68)	poly_C	0.99	0		0	0	general GO
central_nervous_system_development (68)	MUSCLE_INI	0.62	0		0	0	general GO
cellular_defense_response (68)	AML3.01	0.5	H	0	M	0	general GO
cellular_defense_response (68)	ETS2.01	0.48	H	M	0	0	general GO
cellular_defense_response (68)	AP1.01	0.43		H	0	0	general GO
cell_surface_receptor_linked_signal_transduction	ETS2.01	0.48	M	H	0	0	general GO
cell_surface_receptor_linked_signal_transduction	TAL1BETAIT	0.47	0		0	0	general GO
cell_proliferation (243)	TATA.01	0.28		0	0	0	general GO
cell_motility (106)	MEL1_01	0.29	0	0	M	H	general GO
cell_division (103)	GABP.01	0.62	0		0	0	general GO
cell_division (103)	NFY.01	0.52	H		0	0	general GO
cell_division (103)	CAAT.01	0.49	H		0	0	general GO
cell_division (103)	NFY.02	0.48	H		0	0	general GO
cell_division (103)	NFY.03	0.47	0		0	0	general GO
cell_division (103)	CHR.01	0.41		0	0	0	general GO
cell_differentiation (192)	GC_rich	0.99	0	H	0	M	general GO
cell_differentiation (192)	ZNF202.01	0.73	0		0	0	general GO
cell_differentiation (192)	HAND2_E12.	0.7	0		0	0	general GO
cell_cycle (273)	SP1.01	0.78	H		0	0	general GO
cell_cycle (273)	ZF9.01	0.72		0	0	0	general GO
cell_cycle (273)	EGR1.02	0.68	H	M	0	0	general GO
cell_cycle (273)	CDE.01	0.6			0	0	general GO
cell_cycle (273)	WHN.01	0.59	H		0	0	general GO
cell_cycle (273)	NFY.01	0.52	H		0	0	general GO
cell_cycle (273)	CAAT.01	0.49			0	0	general GO
cell_cycle (273)	NFY.02	0.48			0	0	general GO
cell_cycle (273)	NFY.03	0.47	H		0	0	general GO
cell_cycle (273)	CHR.01	0.41		0	0	0	general GO
cell_cell_signaling (268)	NFKAPPAB.(	0.57	H	M	0	0	general GO
cell_cell_signaling (268)	NFKAPPAB6	0.55	H	M	0	0	general GO
cell_cell_signaling (268)	HIVEP1_01	0.52	H	M	0	0	general GO
cell_cell_signaling (268)	MTATA.01	0.47		0	0	0	general GO
cell_cell_signaling (268)	IRF2.01	0.43	0		0	0	general GO
cell_cell_signaling (268)	SRF.01	0.42		0	0	0	general GO
cell_cell_signaling (268)	PDX1_Gabi	0.41		0	0	0	general GO
cell_cell_signaling (268)	LTATA_01	0.39		0	0	0	general GO
cell_cell_signaling (268)	MEF2.05	0.29		0	0	0	general GO
cell_cell_signaling (268)	TATA.01	0.28		0	0	0	general GO
cell_cell_signaling (268)	TATA.02	0.28		0	0	0	general GO
cation_transport (99)	AP2.01	0.73	0	H	H		general GO
cation_transport (99)	NRSF.01	0.62	M	H	0	0	general GO
cation_transport (99)	NRSE.01	0.57	M	0	0	H	general GO
calcium_ion_homeostasis (30)	SRF.03	0.47		0	0	0	general GO
calcium_ion_homeostasis (30)	BCL6.02	0.44	H		0	H	general GO
calcium_ion_homeostasis (30)	TATA.01	0.28		0	0	0	general GO
blood_coagulation (70)	ETS1.01	0.52		0	0	0	general GO
blood_coagulation (70)	HNF1.02	0.34		0	0	0	general GO
blood_coagulation (70)	HNF1.03	0.31		0	0	0	general GO
blood_coagulation (70)	HNF1.01	0.3		0	0	0	general GO
blood_coagulation (70)	SATB1.01	0.27		0	0	0	general GO
blood_coagulation (70)	HNF3B.01	0.26		H	0	0	general GO
biosynthesis (32)	NFY.01	0.52		0	0	0	general GO
biosynthesis (32)	CAAT.01	0.49		H	0	0	general GO
apoptosis (258)	CREL.01	0.55	M		0	0	general GO
antimicrobial_humoral_response (87)	AREB6.01	0.49	H	M	0	0	general GO
antimicrobial_humoral_response (87)	MIT.01	0.46		H	0	0	general GO
antimicrobial_humoral_response (87)	AP1.01	0.43	H	M	0	0	general GO

antimicrobial_humoral_response (87)	ISL1.01	0.43		H	H	0	general GO
antimicrobial_humoral_response (87)	GATA.01	0.41	M	H	0	0	general GO
antimicrobial_humoral_response (87)	MTBF.01	0.4		0	0	0	general GO
antimicrobial_humoral_response (87)	OCT1.05	0.39		H	0	0	general GO
antimicrobial_humoral_response (87)	LTATA_01	0.39		H	0	0	general GO
antimicrobial_humoral_response (87)	GATA1.03	0.39		H	0	0	general GO
antimicrobial_humoral_response (87)	CDX2.01	0.38		0	0	0	general GO
antimicrobial_humoral_response (87)	GATA1.05	0.36		H	0	0	general GO
antimicrobial_humoral_response (87)	OCT.01	0.35		H	0	H	general GO
antimicrobial_humoral_response (87)	CDX1.01	0.35		H	0	H	general GO
antimicrobial_humoral_response (87)	EVI1.04	0.33	H	M	0	0	general GO
antimicrobial_humoral_response (87)	EN1.01	0.32	H		0	0	general GO
antimicrobial_humoral_response (87)	HNF6.01	0.31	M	H	0	0	general GO
antimicrobial_humoral_response (87)	XVENT2.01	0.3		H	0	0	general GO
amino_acid_metabolism (45)	ERR_01	0.52	H	M	0	0	general GO

400bp Window

GO group	Motifs	motif GC	-199 - 200	201 - 600	601 - 1000	GO-classes
development (530)	GC_rich	1	H			transcription GO
development (530)	poly_C	1				transcription GO
development (530)	SP1.01	0.8	0			transcription GO
development (530)	WT1.01	0.8	H			transcription GO
development (530)	HES1.02	0.8	0		M	transcription GO
development (530)	MAZR.01	0.8				transcription GO
development (530)	ZNF202.01	0.7				transcription GO
development (530)	AP2.01	0.7	H			transcription GO
development (530)	ZF9.01	0.7	0			transcription GO
development (530)	EGR3.01	0.7	0	0		transcription GO
development (530)	MYCMAX.03	0.7	H		0	transcription GO
development (530)	NFKAPPAB5(	0.7	0		H	transcription GO
development (530)	HAND2_E12.	0.7	0		H	transcription GO
development (530)	ZBP89.01	0.7				transcription GO
development (530)	NGFIC.01	0.7	0	H	M	transcription GO
development (530)	CKROX_01	0.7				transcription GO
development (530)	EGR1.02	0.7	H			transcription GO
development (530)	HIC1_01	0.7	0	H		transcription GO
development (530)	EGR2.01	0.7	0	0		transcription GO
development (530)	GC.01	0.7	0		0	transcription GO
development (530)	MZF1.01	0.7	H		H	transcription GO
development (530)	MAZ.01	0.7	H			transcription GO
development (530)	ZIC2_01	0.6	0	H		transcription GO
development (530)	PLAG1_01	0.6				transcription GO
development (530)	INSM1_01	0.6	0	H		transcription GO
development (530)	MUSCLE_INI	0.6	0		H	transcription GO
development (530)	MUSCLE_INI	0.6	0		H	transcription GO
development (530)	CDE.01	0.6	0	H		transcription GO
development (530)	AHR.01	0.6	0	0		transcription GO
development (530)	E2F.02	0.6	0	0		transcription GO
development (530)	NRSE.01	0.6	0		H	transcription GO
development (530)	PAX5.01	0.6	0	H		transcription GO
development (530)	GAGA.01	0.6				transcription GO
development (530)	AG_rich_codi	0.5		H	H	transcription GO
regulation_of_transcription (266)	GC_rich	1				transcription GO
regulation_of_transcription (266)	poly_C	1				transcription GO
regulation_of_transcription (266)	SP1.01	0.8	H		M	transcription GO
regulation_of_transcription (266)	WT1.01	0.8	H		H	transcription GO
regulation_of_transcription (266)	MAZR.01	0.8				transcription GO
regulation_of_transcription (266)	ZNF202.01	0.7				transcription GO
regulation_of_transcription (266)	AP2.01	0.7	H		H	transcription GO
regulation_of_transcription (266)	ZF5.01	0.7	H		H	transcription GO
regulation_of_transcription (266)	ZF9.01	0.7	0			transcription GO
regulation_of_transcription (266)	EGR3.01	0.7	H	0		transcription GO
regulation_of_transcription (266)	ZBP89.01	0.7			H	transcription GO
regulation_of_transcription (266)	NGFIC.01	0.7	H	0	M	transcription GO
regulation_of_transcription (266)	CKROX_01	0.7			H	transcription GO
regulation_of_transcription (266)	EGR1.02	0.7				transcription GO
regulation_of_transcription (266)	EGR2.01	0.7	H	0		transcription GO
regulation_of_transcription (266)	GC.01	0.7	0		0	transcription GO
regulation_of_transcription (266)	MZF1.01	0.7			H	transcription GO

regulation_of_transcription (266)	EGR1.01	0.7	H	0	M	transcription GO
regulation_of_transcription (266)	MAZ.01	0.7				transcription GO
regulation_of_transcription (266)	ZIC2_01	0.6	0	M	H	transcription GO
regulation_of_transcription (266)	PLAG1_01	0.6			H	transcription GO
regulation_of_transcription (266)	RREB1.01	0.6	0	H		transcription GO
regulation_of_transcription (266)	CDE.01	0.6	H			transcription GO
regulation_of_transcription (266)	E2F.02	0.6	0	H		transcription GO
regulation_of_transcription (266)	PAX5.01	0.6	0		H	transcription GO
regulation_of_transcription (266)	GAGA.01	0.6			0	transcription GO
regulation_of_transcription (266)	NFY.01	0.5	H		0	transcription GO
regulation_of_transcription (266)	AG_rich_codi	0.5			H	transcription GO
regulation_of_transcription (266)	GKLF.01	0.5		H	0	transcription GO
regulation_of_transcription (266)	CAAT.01	0.5		0	0	transcription GO
regulation_of_transcription (266)	PBX1_MEIS1	0.5	H	M	0	transcription GO
regulation_of_transcription (266)	PBX1_MEIS1	0.4	H	M	0	transcription GO
regulation_of_transcription_DNA_dependent (1066)	GC_rich	1				transcription GO
regulation_of_transcription_DNA_dependent (1066)	poly_C	1				transcription GO
regulation_of_transcription_DNA_dependent (1066)	SP1.01	0.8	H			transcription GO
regulation_of_transcription_DNA_dependent (1066)	WT1.01	0.8				transcription GO
regulation_of_transcription_DNA_dependent (1066)	HES1.02	0.8	H		H	transcription GO
regulation_of_transcription_DNA_dependent (1066)	MAZR.01	0.8				transcription GO
regulation_of_transcription_DNA_dependent (1066)	ZNF202.01	0.7				transcription GO
regulation_of_transcription_DNA_dependent (1066)	AP2.01	0.7				transcription GO
regulation_of_transcription_DNA_dependent (1066)	ZF5.01	0.7				transcription GO
regulation_of_transcription_DNA_dependent (1066)	ZF9.01	0.7	H			transcription GO
regulation_of_transcription_DNA_dependent (1066)	EGR3.01	0.7	H			transcription GO
regulation_of_transcription_DNA_dependent (1066)	NRF1_01	0.7	H			transcription GO
regulation_of_transcription_DNA_dependent (1066)	MYCMAX.03	0.7	H		H	transcription GO
regulation_of_transcription_DNA_dependent (1066)	NFKAPPAB50	0.7	0	M		transcription GO
regulation_of_transcription_DNA_dependent (1066)	ZBP89.01	0.7				transcription GO
regulation_of_transcription_DNA_dependent (1066)	HELT.01	0.7	H	H		transcription GO
regulation_of_transcription_DNA_dependent (1066)	NGFIC.01	0.7	H			transcription GO
regulation_of_transcription_DNA_dependent (1066)	CKROX_01	0.7				transcription GO
regulation_of_transcription_DNA_dependent (1066)	EGR1.02	0.7				transcription GO
regulation_of_transcription_DNA_dependent (1066)	HIC1_01	0.7	0	M		transcription GO
regulation_of_transcription_DNA_dependent (1066)	BKLF.01	0.7	0			transcription GO
regulation_of_transcription_DNA_dependent (1066)	EGR2.01	0.7	H			transcription GO
regulation_of_transcription_DNA_dependent (1066)	GC.01	0.7	0			transcription GO
regulation_of_transcription_DNA_dependent (1066)	MZF1.01	0.7			H	transcription GO
regulation_of_transcription_DNA_dependent (1066)	EGR1.01	0.7	H			transcription GO
regulation_of_transcription_DNA_dependent (1066)	MTF-1.01	0.7	0		H	transcription GO
regulation_of_transcription_DNA_dependent (1066)	MAZ.01	0.7				transcription GO
regulation_of_transcription_DNA_dependent (1066)	ZIC2_01	0.6	H		H	transcription GO
regulation_of_transcription_DNA_dependent (1066)	ATF6.01	0.6	M		H	transcription GO
regulation_of_transcription_DNA_dependent (1066)	PLAG1_01	0.6				transcription GO
regulation_of_transcription_DNA_dependent (1066)	EBVR.01	0.6	H			transcription GO
regulation_of_transcription_DNA_dependent (1066)	AHRARNT.02	0.6	H	H		transcription GO
regulation_of_transcription_DNA_dependent (1066)	HES1.01	0.6				transcription GO
regulation_of_transcription_DNA_dependent (1066)	MUSCLE_INI	0.6	H			transcription GO
regulation_of_transcription_DNA_dependent (1066)	GABP.01	0.6	0		H	transcription GO
regulation_of_transcription_DNA_dependent (1066)	HIF1.01	0.6	0		H	transcription GO
regulation_of_transcription_DNA_dependent (1066)	MUSCLE_INI	0.6	H		H	transcription GO
regulation_of_transcription_DNA_dependent (1066)	EKLF.01	0.6	0			transcription GO
regulation_of_transcription_DNA_dependent (1066)	RREB1.01	0.6	0	H		transcription GO
regulation_of_transcription_DNA_dependent (1066)	CDE.01	0.6				transcription GO
regulation_of_transcription_DNA_dependent (1066)	AHR.01	0.6	0	H		transcription GO
regulation_of_transcription_DNA_dependent (1066)	E2F.02	0.6	H	H		transcription GO
regulation_of_transcription_DNA_dependent (1066)	WHN.01	0.6	H	H		transcription GO
regulation_of_transcription_DNA_dependent (1066)	TAXCREB.01	0.6	0		0	transcription GO
regulation_of_transcription_DNA_dependent (1066)	PAX9.01	0.6	0	H		transcription GO
regulation_of_transcription_DNA_dependent (1066)	NRSE.01	0.6	0	M	H	transcription GO
regulation_of_transcription_DNA_dependent (1066)	VDR_RXR.02	0.6	H	M	0	transcription GO
regulation_of_transcription_DNA_dependent (1066)	GAGA.01	0.6				transcription GO
regulation_of_transcription_DNA_dependent (1066)	E2F.03	0.5	H	H		transcription GO

regulation_of_transcription_DNA_dependent (1066)	NFY.01	0.5	H		0	transcription GO
regulation_of_transcription_DNA_dependent (1066)	AG_rich_codi	0.5			0	transcription GO
regulation_of_transcription_DNA_dependent (1066)	E2F.01	0.5	H	0	M	transcription GO
regulation_of_transcription_DNA_dependent (1066)	GKLF.01	0.5		M	0	transcription GO
regulation_of_transcription_DNA_dependent (1066)	CAAT.01	0.5		H	0	transcription GO
regulation_of_transcription_from_RNA_polymerase_II_promoter (163)	SP1.01	0.8	H		0	transcription GO
regulation_of_transcription_from_RNA_polymerase_II_promoter (163)	WT1.01	0.8		H	H	transcription GO
regulation_of_transcription_from_RNA_polymerase_II_promoter (163)	MAZR.01	0.8		H	0	transcription GO
regulation_of_transcription_from_RNA_polymerase_II_promoter (163)	ZF5.01	0.7	H	0	M	transcription GO
regulation_of_transcription_from_RNA_polymerase_II_promoter (163)	CKROX_01	0.7		H	0	transcription GO
regulation_of_transcription_from_RNA_polymerase_II_promoter (163)	MAZ.01	0.7			0	transcription GO
regulation_of_transcription_from_RNA_polymerase_II_promoter (163)	EBVR.01	0.6	H	0	M	transcription GO
regulation_of_transcription_from_RNA_polymerase_II_promoter (163)	HES1.01	0.6	H	0	M	transcription GO
regulation_of_transcription_from_RNA_polymerase_II_promoter (163)	CDE.01	0.6	H	0		transcription GO
transcription (930)	GC_rich	1				transcription GO
transcription (930)	poly_C	1				transcription GO
transcription (930)	SP1.01	0.8	H			transcription GO
transcription (930)	WT1.01	0.8				transcription GO
transcription (930)	HES1.02	0.8	H		H	transcription GO
transcription (930)	MAZR.01	0.8				transcription GO
transcription (930)	ZNF202.01	0.7				transcription GO
transcription (930)	AP2.01	0.7				transcription GO
transcription (930)	ZF5.01	0.7				transcription GO
transcription (930)	ZF9.01	0.7	H			transcription GO
transcription (930)	EGR3.01	0.7	H			transcription GO
transcription (930)	NRF1_01	0.7	H			transcription GO
transcription (930)	MYCMAX.03	0.7	H		H	transcription GO
transcription (930)	ZBP89.01	0.7				transcription GO
transcription (930)	HELT.01	0.7	H			transcription GO
transcription (930)	NGFIC.01	0.7	H		H	transcription GO
transcription (930)	CKROX_01	0.7				transcription GO
transcription (930)	EGR1.02	0.7				transcription GO
transcription (930)	HIC1_01	0.7	0	0		transcription GO
transcription (930)	GC.01	0.7	H			transcription GO
transcription (930)	MZF1.01	0.7		0	H	transcription GO
transcription (930)	EGR1.01	0.7	H		H	transcription GO
transcription (930)	MTF-1.01	0.7	0	M	H	transcription GO
transcription (930)	MAZ.01	0.7				transcription GO
transcription (930)	ZIC2_01	0.6	H		0	transcription GO
transcription (930)	PLAG1_01	0.6				transcription GO
transcription (930)	EBVR.01	0.6	H	H		transcription GO
transcription (930)	AHRARNT.02	0.6		H		transcription GO
transcription (930)	HES1.01	0.6			H	transcription GO
transcription (930)	MUSCLE_INI	0.6	H		H	transcription GO
transcription (930)	HIF1.01	0.6	H		H	transcription GO
transcription (930)	MUSCLE_INI	0.6	H		H	transcription GO
transcription (930)	EKLF.01	0.6	0	0		transcription GO
transcription (930)	CDE.01	0.6				transcription GO
transcription (930)	AHR.01	0.6	H	H	M	transcription GO
transcription (930)	E2F.02	0.6	H	H		transcription GO
transcription (930)	WHN.01	0.6	H	H		transcription GO
transcription (930)	TAXCREB.01	0.6	0		0	transcription GO
transcription (930)	PAX9.01	0.6	0	H		transcription GO
transcription (930)	NRSE.01	0.6	0	M	H	transcription GO
transcription (930)	PAX5.03	0.6	H	M	0	transcription GO
transcription (930)	GAGA.01	0.6			0	transcription GO
transcription (930)	E2F.03	0.5	H	H		transcription GO
transcription (930)	AG_rich_codi	0.5			0	transcription GO
transcription (930)	E2F.01	0.5	H	0	M	transcription GO
transcription_RNA_polyII_promoter (190)	GC_rich	1	H		0	general GO
transcription_RNA_polyII_promoter (190)	SP1.01	0.8	0		0	general GO
transcription_RNA_polyII_promoter (190)	WT1.01	0.8	H		0	general GO
transcription_RNA_polyII_promoter (190)	MAZR.01	0.8		H	0	general GO
transcription_RNA_polyII_promoter (190)	ZNF202.01	0.7	H		0	general GO

transcription_RNA_polyII_promoter (190)	AP2.01	0.7	H	M	H	general GO
transcription_RNA_polyII_promoter (190)	ZF9.01	0.7	0			general GO
transcription_RNA_polyII_promoter (190)	NRF1_01	0.7	0		0	general GO
transcription_RNA_polyII_promoter (190)	MYCMAX.03	0.7	H	M	0	general GO
transcription_RNA_polyII_promoter (190)	EGR1.02	0.7	H		0	general GO
transcription_RNA_polyII_promoter (190)	HIC1_01	0.7	0	0		general GO
transcription_RNA_polyII_promoter (190)	MAZ.01	0.7	H		0	general GO
transcription_RNA_polyII_promoter (190)	PLAG1_01	0.6	M	H	H	general GO
transcription_RNA_polyII_promoter (190)	MUSCLE_INI	0.6	0		0	general GO
transcription_RNA_polyII_promoter (190)	CDE.01	0.6	H			general GO
transcription_RNA_polyII_promoter (190)	WHN.01	0.6	H	0	M	general GO
actin_cytoskeleton_organization_and_biogenesis (45)	GC_rich	1		0	0	general GO
actin_cytoskeleton_organization_and_biogenesis (45)	NRF1_01	0.7		0	0	general GO
anti_apoptosis (85)	NFKAPPAB6	0.6		0	0	general GO
antimicrobial_humoral_response (87)	ETS1.01	0.5		0	0	general GO
antimicrobial_humoral_response (87)	AREB6.01	0.5		0	0	general GO
antimicrobial_humoral_response (87)	GATA1.01	0.4		0	0	general GO
antimicrobial_humoral_response (87)	ISRE.01	0.4		0	0	general GO
antimicrobial_humoral_response (87)	HMGIY.01	0.4	M	H	0	general GO
antimicrobial_humoral_response (87)	GATA.01	0.4		0	0	general GO
antimicrobial_humoral_response (87)	MTBF.01	0.4		0	0	general GO
antimicrobial_humoral_response (87)	CEBPB.01	0.4		0	0	general GO
antimicrobial_humoral_response (87)	MEIS1B_HO	0.4		0	0	general GO
antimicrobial_humoral_response (87)	OCT1.05	0.4		H	0	general GO
antimicrobial_humoral_response (87)	LTATA_01	0.4		0	0	general GO
antimicrobial_humoral_response (87)	IRF4.01	0.4		0	0	general GO
antimicrobial_humoral_response (87)	GATA1.03	0.4		0	M	general GO
antimicrobial_humoral_response (87)	GFI1B.01	0.4		0	0	general GO
antimicrobial_humoral_response (87)	CDX2.01	0.4		0	H	general GO
antimicrobial_humoral_response (87)	GATA1.05	0.4		0	0	general GO
antimicrobial_humoral_response (87)	OCT.01	0.4		H	0	general GO
antimicrobial_humoral_response (87)	CDX1.01	0.3		0	H	general GO
antimicrobial_humoral_response (87)	FKHRL1.01	0.3		0	0	general GO
antimicrobial_humoral_response (87)	HNF6.01	0.3		H	0	general GO
antimicrobial_humoral_response (87)	XVENT2.01	0.3		0	0	general GO
antimicrobial_humoral_response (87)	ATATA.01	0.3		H	0	general GO
antimicrobial_humoral_response (87)	CDP.02	0.3		0	0	general GO
antimicrobial_humoral_response (87)	MEF2.04	0.3		0	0	general GO
antimicrobial_humoral_response (87)	TATA.01	0.3		0	H	general GO
antimicrobial_humoral_response (87)	EVI1.03	0.2		0	0	general GO
apoptosis (258)	NFKAPPAB.0	0.6		0	0	general GO
apoptosis (258)	CREL.01	0.5	M	H	0	general GO
biosynthesis (32)	CAAT.01	0.5		0	0	general GO
biosynthesis (32)	NFY.02	0.5		0	0	general GO
blood_coagulation (70)	ARE.01	0.5	M	H	0	general GO
blood_coagulation (70)	BCL6.02	0.4	M	H	0	general GO
blood_coagulation (70)	AIRE.01	0.4	0	H	M	general GO
blood_coagulation (70)	FREAC4.01	0.4		0	0	general GO
blood_coagulation (70)	HNF1.02	0.3		0	0	general GO
blood_coagulation (70)	FREAC3.01	0.3	H	0	M	general GO
blood_coagulation (70)	HNF1.03	0.3		0	0	general GO
blood_coagulation (70)	HNF1.01	0.3		0	0	general GO
blood_coagulation (70)	DLX1.01	0.3	M	H	0	general GO
calcium_ion_homeostasis (30)	TATA.01	0.3		0	0	general GO
cation_transport (99)	SP1.01	0.8	0	H	M	general GO
cation_transport (99)	ZNF202.01	0.7		H	H	general GO
cation_transport (99)	NFKAPPAB5	0.7	H	0	M	general GO
cation_transport (99)	NRSE.01	0.6	M	0	H	general GO
cell_adhesion (378)	PDX1_Gabi	0.4	M	H	0	general GO
cell_cell_signaling (268)	NFKAPPAB6	0.6		M	0	general GO
cell_cell_signaling (268)	CREL.01	0.5	H	M	0	general GO
cell_cell_signaling (268)	NFKAPPAB.0	0.5		0	0	general GO
cell_cell_signaling (268)	HIVEP1_01	0.5	H	M	0	general GO
cell_cell_signaling (268)	MTATA.01	0.5		0	0	general GO

cell_cell_signaling (268)	AARE.01	0.4		0	0	general GO
cell_cell_signaling (268)	LTATA_01	0.4		0	0	general GO
cell_cell_signaling (268)	NFAT.01	0.4		0	0	general GO
cell_cell_signaling (268)	CDX2.01	0.4		0	0	general GO
cell_cell_signaling (268)	MEF2.05	0.3		0	0	general GO
cell_cell_signaling (268)	TATA.01	0.3		0	0	general GO
cell_cell_signaling (268)	TATA.02	0.3		0	0	general GO
cell_cell_signaling (268)	BRN2.02	0.2		0	0	general GO
cell_cycle (273)	NRF1_01	0.7		0	0	general GO
cell_cycle (273)	CDE.01	0.6		H	0	general GO
cell_cycle (273)	E2F.03	0.5	H		0	general GO
cell_cycle (273)	NFY.01	0.5			0	general GO
cell_cycle (273)	CAAT.01	0.5		H	0	general GO
cell_cycle (273)	NFY.02	0.5		H	0	general GO
cell_cycle (273)	CREB.01	0.5	H	M	0	general GO
cell_cycle (273)	NFY.03	0.5		H	0	general GO
cell_cycle (273)	CHR.01	0.4		0	0	general GO
cell_differentiation (192)	WT1.01	0.8	0		0	general GO
cell_division (103)	SP2_01	0.6	H	M	0	general GO
cell_division (103)	NFY.01	0.5		H	0	general GO
cell_division (103)	CAAT.01	0.5		H	0	general GO
cell_division (103)	NFY.02	0.5		0	0	general GO
cell_division (103)	NFY.03	0.5		0	0	general GO
cell_division (103)	CHR.01	0.4		0	0	general GO
cell_matrix_adhesion (56)	HBP1_01	0.4	0		0	general GO
cell_motility (106)	TATA.02	0.3		0	0	general GO
cell_surface_receptor_linked_signal_transduction (169)	ETS2.01	0.5		0	0	general GO
cell_surface_receptor_linked_signal_transduction (169)	AP1.01	0.4		H	0	general GO
cell_surface_receptor_linked_signal_transduction (169)	OCT1.05	0.4		0	0	general GO
cell_surface_receptor_linked_signal_transduction (169)	GATA1.05	0.4	H	M	0	general GO
cell_surface_receptor_linked_signal_transduction (169)	EN1.01	0.3	M	H	0	general GO
cellular_defense_response (68)	NFKAPPAB.0	0.5		0	0	general GO
cellular_defense_response (68)	GATA1.03	0.4		H	0	general GO
central_nervous_system_development (68)	MAZR.01	0.8	0		0	general GO
central_nervous_system_development (68)	MUSCLE_INI	0.6	0		0	general GO
chemotaxis (98)	NFKAPPAB.0	0.6		0	0	general GO
chemotaxis (98)	NFKAPPAB.0	0.6		0	0	general GO
chemotaxis (98)	NFKAPPAB6	0.6		0	0	general GO
chemotaxis (98)	CREL.01	0.5		0	0	general GO
chemotaxis (98)	NFKAPPAB.0	0.5		0	0	general GO
chemotaxis (98)	HIVEP1_01	0.5		0	0	general GO
chemotaxis (98)	NFE2.01	0.5		0	0	general GO
chemotaxis (98)	IK3.01	0.5		0	0	general GO
chemotaxis (98)	NFE2L2.01	0.5		0	H	general GO
chemotaxis (98)	PXRCAR.01	0.5		0	0	general GO
chemotaxis (98)	BARBIE.01	0.4		0	H	general GO
chemotaxis (98)	GATA1.01	0.4		0	0	general GO
chemotaxis (98)	MYT1L.01	0.4	H	0	M	general GO
chemotaxis (98)	TCF11MAFG	0.4		0	0	general GO
chemotaxis (98)	AP1.01	0.4		0	0	general GO
chemotaxis (98)	SRF.01	0.4		0	0	general GO
chemotaxis (98)	HMGIY.01	0.4		H	0	general GO
chemotaxis (98)	GATA1.04	0.4		0	0	general GO
chemotaxis (98)	AIRE.01	0.4		H	0	general GO
chemotaxis (98)	LTATA_01	0.4		0	0	general GO
chemotaxis (98)	GATA1.03	0.4		H	0	general GO
chemotaxis (98)	NFAT.01	0.4		0	0	general GO
chemotaxis (98)	CDX2.01	0.4		H	0	general GO
chemotaxis (98)	IRF7.01	0.4		H	0	general GO
chemotaxis (98)	GATA2.01	0.4		0	H	general GO
chemotaxis (98)	CDX1.01	0.3		0	0	general GO
chemotaxis (98)	GATA3.01	0.3		0	0	general GO
chemotaxis (98)	TATA.01	0.3		0	0	general GO
chemotaxis (98)	TATA.02	0.3		H	H	general GO

chromatin_modification (53)	WT1.01	0.8	0		0	general GO
chromatin_modification (53)	ZNF202.01	0.7	M	0	H	general GO
chromatin_modification (53)	CKROX_01	0.7		0	0	general GO
chromosome_organization_and_biogenesis (83)	ACAAT.01	0.5		0	0	general GO
chromosome_organization_and_biogenesis (83)	CAAT.01	0.5		0	0	general GO
chromosome_organization_and_biogenesis (83)	NFY.02	0.5		H	0	general GO
chromosome_organization_and_biogenesis (83)	NFY.03	0.5		0	0	general GO
chromosome_organization_and_biogenesis (83)	BRN2.03	0.3		H	H	general GO
chromosome_organization_and_biogenesis (83)	TATA.01	0.3		H	0	general GO
circulation (52)	SRF.01	0.4	0	H	M	general GO
defense_response (100)	NFKAPPAB.0	0.5		0	0	general GO
defense_response (100)	HIVEP1_01	0.5		0	0	general GO
defense_response (100)	HOX1-3.01	0.4		0	0	general GO
defense_response (100)	PRDM1.01	0.4		0	0	general GO
defense_response (100)	CEBPB.01	0.4		0	0	general GO
defense_response (100)	HOXA9.01	0.3	0	M	H	general GO
defense_response (100)	MEF2.04	0.3	0		0	general GO
development (530)	GKLF.01	0.5	H		0	general GO
digestion (51)	NFAT.01	0.4		0	0	general GO
digestion (51)	MEL1_01	0.3		H	0	general GO
digestion (51)	TATA.01	0.3		0	0	general GO
digestion (51)	FREAC7.01	0.3		0	0	general GO
digestion (51)	HNF3B.01	0.3		0	0	general GO
digestion (51)	EVI1.03	0.2		0	0	general GO
DNA_metabolism (47)	E2F.02	0.6		0	0	general GO
DNA_metabolism (47)	E2F.03	0.5		0	0	general GO
DNA_metabolism (47)	E2F.01	0.5		0	0	general GO
DNA_recombination (38)	CAAT.01	0.5		0	0	general GO
DNA_recombination (38)	NFY.03	0.5		0	H	general GO
DNA_repair (148)	E2F.02	0.6		0	0	general GO
DNA_repair (148)	ELK1.02	0.5		0	0	general GO
DNA_repair (148)	E2F.03	0.5		0	0	general GO
DNA_repair (148)	FLI.01	0.5		0	0	general GO
DNA_repair (148)	E2F.01	0.5		0	0	general GO
DNA_replication (91)	NFY.01	0.5		0	0	general GO
electron_transport (214)	HNF4.01	0.5		0	0	general GO
electron_transport (214)	ERR_01	0.5		0	0	general GO
electron_transport (214)	SF1.01	0.5		0	0	general GO
electron_transport (214)	PPARA.01	0.5	H	M	0	general GO
electron_transport (214)	PLZF.01	0.5	0		0	general GO
electron_transport (214)	RORA1.01	0.4		0	0	general GO
elevation_of_cytosolic_calcium_ion_concentration (37)	AMEF2.01	0.4		0	0	general GO
endocytosis (65)	DEC1.01	0.6		0	0	general GO
epidermis_development (59)	HEN1.01	0.6	0	M	H	general GO
epidermis_development (59)	BACH2.01	0.5	H	0	M	general GO
epidermis_development (59)	AML3.01	0.5	M	0	H	general GO
epidermis_development (59)	TAL1ALPHAE	0.5		0	0	general GO
epidermis_development (59)	TBX5.01	0.5		0	0	general GO
epidermis_development (59)	BACH1.01	0.5	H	0	M	general GO
epidermis_development (59)	AP1.01	0.4	H	0	M	general GO
epidermis_development (59)	TATA.01	0.3		0	0	general GO
epidermis_development (59)	TATA.02	0.3		0	0	general GO
ER_to_Golgi_transport (47)	ATF6.02	0.6		0	0	general GO
ER_to_Golgi_transport (47)	CREB.02	0.6		0	0	general GO
ER_to_Golgi_transport (47)	E4F.01	0.5		0	0	general GO
ER_to_Golgi_transport (47)	ATF.02	0.5		0	0	general GO
ER_to_Golgi_transport (47)	CREBP1CJU	0.4		0	0	general GO
fatty_acid_metabolism (55)	BARX2_01	0.3	0	H	M	general GO
G_protein_coupled_receptor_protein_signaling_pathway (398)	NRSF.01	0.6	0		0	general GO
G_protein_coupled_receptor_protein_signaling_pathway (398)	TAL1BETAIT	0.5	H		0	general GO
G_protein_coupled_receptor_protein_signaling_pathway (398)	MEL1_02	0.5		0	0	general GO
G_protein_coupled_receptor_protein_signaling_pathway (398)	COMP1.01	0.5		0	0	general GO
G_protein_coupled_receptor_protein_signaling_pathway (398)	BRACH.01	0.4		0	0	general GO
G_protein_coupled_receptor_protein_signaling_pathway (398)	TCF11MAFG	0.4		0	0	general GO

G_protein_coupled_receptor_protein_signaling_pathway (398)	ISL1.01	0.4		H	0	general GO
G_protein_coupled_receptor_protein_signaling_pathway (398)	BRN4.01	0.4		0	0	general GO
G_protein_coupled_receptor_protein_signaling_pathway (398)	GATA1.04	0.4		H	0	general GO
G_protein_coupled_receptor_protein_signaling_pathway (398)	FREAC4.01	0.4		H	0	general GO
G_protein_coupled_receptor_protein_signaling_pathway (398)	GATA2.02	0.4	H		0	general GO
G_protein_coupled_receptor_protein_signaling_pathway (398)	XFD3.01	0.4		0	0	general GO
G_protein_coupled_receptor_protein_signaling_pathway (398)	GATA2.01	0.4		H	0	general GO
G_protein_coupled_receptor_protein_signaling_pathway (398)	GATA3.01	0.3			0	general GO
G_protein_coupled_receptor_protein_signaling_pathway (398)	EVI1.02	0.3		0	0	general GO
G_protein_coupled_receptor_protein_signaling_pathway (398)	FREAC3.01	0.3		0	0	general GO
G_protein_coupled_receptor_protein_signaling_pathway (398)	CART1.01	0.3		H	0	general GO
G_protein_coupled_receptor_protein_signaling_pathway (398)	EVI1.01	0.3		H	0	general GO
G_protein_coupled_receptor_protein_signaling_pathway (398)	MEL1_01	0.3		H	0	general GO
G_protein_coupled_receptor_protein_signaling_pathway (398)	EVI1.05	0.3		0	0	general GO
G_protein_coupled_receptor_protein_signaling_pathway (398)	PIT1.01	0.3		0	0	general GO
G_protein_coupled_receptor_protein_signaling_pathway (398)	HNF3B.01	0.3		H	0	general GO
G_protein_coupled_receptor_protein_signaling_pathway (398)	NKX31.01	0.2		H	H	general GO
G_protein_coupled_receptor_protein_signaling_pathway (398)	BRN2.02	0.2		H	0	general GO
G_protein_coupled_receptor_protein_signaling_pathway (398)	EVI1.03	0.2		H	0	general GO
G_protein_coupled_receptor_protein_signaling_pathway (398)	AT_rich	0		0	0	general GO
generation_of_precursor_metabolites_and_energy (85)	ER.02	0.5	M	0	H	general GO
homophilic_cell_adhesion (70)	NFAT.01	0.4		0	0	general GO
homophilic_cell_adhesion (70)	OCT1P.01	0.4		0	H	general GO
immune_response (366)	NFKAPPAB.0	0.6		H	0	general GO
immune_response (366)	NFKAPPAB.0	0.6		H	0	general GO
immune_response (366)	NFKAPPAB6	0.6		H	0	general GO
immune_response (366)	CREL.01	0.5		H	0	general GO
immune_response (366)	NFKAPPAB.0	0.5		H	0	general GO
immune_response (366)	HIVEP1_01	0.5		H	0	general GO
immune_response (366)	AML1.01	0.5		0	0	general GO
immune_response (366)	PARAXIS_01	0.5	H	0	M	general GO
immune_response (366)	ETS1.01	0.5		H	0	general GO
immune_response (366)	AML3.01	0.5		H	0	general GO
immune_response (366)	RBPJK.02	0.5		H	0	general GO
immune_response (366)	TAL1ALPHAE	0.5		H	0	general GO
immune_response (366)	NFE2.01	0.5		0	0	general GO
immune_response (366)	TR2.01	0.5		0	0	general GO
immune_response (366)	TAL1BETAE4	0.5			0	general GO
immune_response (366)	AREB6.01	0.5	M	0	H	general GO
immune_response (366)	TR4.01	0.5		0	0	general GO
immune_response (366)	LXRE.01	0.5		0	0	general GO
immune_response (366)	NKX32.01	0.5		0	0	general GO
immune_response (366)	ETS2.01	0.5		H	0	general GO
immune_response (366)	GRE.01	0.5		0	0	general GO
immune_response (366)	PAX2.01	0.5		H	0	general GO
immune_response (366)	PPARA.01	0.5		H	0	general GO
immune_response (366)	NFE2L2.01	0.5		H	0	general GO
immune_response (366)	MTATA.01	0.5		0	0	general GO
immune_response (366)	TAL1BETAIT	0.5	0		0	general GO
immune_response (366)	SRF.03	0.5	H		0	general GO
immune_response (366)	BACH1.01	0.5		H	0	general GO
immune_response (366)	PLZF.01	0.5		0	0	general GO
immune_response (366)	AREB6.04	0.5		0	0	general GO
immune_response (366)	GKLF_02	0.5		0	0	general GO
immune_response (366)	TAACC.01	0.5		0	0	general GO
immune_response (366)	STAT6.01	0.5		H	0	general GO
immune_response (366)	MIT.01	0.5			H	general GO
immune_response (366)	MEL1_03	0.5		H	H	general GO
immune_response (366)	RP58.01	0.5		H	0	general GO
immune_response (366)	COMP1.01	0.5		0	0	general GO
immune_response (366)	PXRCAR.01	0.5		H	0	general GO
immune_response (366)	BARBIE.01	0.4	H	M	H	general GO
immune_response (366)	BRACH.01	0.4		0	0	general GO
immune_response (366)	PAX8.01	0.4		H	0	general GO

immune_response (366)	GATA1.01	0.4		0	H	general GO
immune_response (366)	RTR.01	0.4		H	H	general GO
immune_response (366)	MYT1L.01	0.4		H	0	general GO
immune_response (366)	STAT.01	0.4		H	0	general GO
immune_response (366)	PRE.01	0.4		0	0	general GO
immune_response (366)	TCF11.01	0.4	H		0	general GO
immune_response (366)	BCL6.02	0.4		0	0	general GO
immune_response (366)	TCF11MAFG	0.4		H	0	general GO
immune_response (366)	ILF1_01	0.4		H	0	general GO
immune_response (366)	AP1.01	0.4		H	0	general GO
immune_response (366)	ISRE.01	0.4		M	0	general GO
immune_response (366)	IRF2.01	0.4		0	0	general GO
immune_response (366)	IRF3.01	0.4		0	0	general GO
immune_response (366)	SRF.01	0.4			0	general GO
immune_response (366)	NKX25.01	0.4		0	0	general GO
immune_response (366)	PRDM1.01	0.4			0	general GO
immune_response (366)	HMGIY.01	0.4		H	0	general GO
immune_response (366)	PDX1_Gabi	0.4	M	H	0	general GO
immune_response (366)	FAST1.01	0.4		H	0	general GO
immune_response (366)	GATA.01	0.4		H	0	general GO
immune_response (366)	HBP1_01	0.4		0	0	general GO
immune_response (366)	RORA1.01	0.4		H	0	general GO
immune_response (366)	GFI1.01	0.4		0	M	general GO
immune_response (366)	PHOX2_01	0.4		H	0	general GO
immune_response (366)	PSE_02	0.4		0	H	general GO
immune_response (366)	BRN4.01	0.4		H	0	general GO
immune_response (366)	LEF1.02	0.4		H	0	general GO
immune_response (366)	MTBF.01	0.4		H	0	general GO
immune_response (366)	IRF1.01	0.4		0	0	general GO
immune_response (366)	CEBPB.01	0.4		H	0	general GO
immune_response (366)	GATA1.04	0.4		H	0	general GO
immune_response (366)	OCT1.05	0.4		H	0	general GO
immune_response (366)	AARE.01	0.4		0	0	general GO
immune_response (366)	AIRE.01	0.4		H	H	general GO
immune_response (366)	LTATA_01	0.4		0	0	general GO
immune_response (366)	BRN2.01	0.4		H	0	general GO
immune_response (366)	IRF4.01	0.4		H	0	general GO
immune_response (366)	STAT5.01	0.4			0	general GO
immune_response (366)	GATA1.03	0.4		H	0	general GO
immune_response (366)	BCL6.01	0.4		M	0	general GO
immune_response (366)	OCT1.04	0.4			0	general GO
immune_response (366)	GFI1B.01	0.4		0	0	general GO
immune_response (366)	PDX1.01	0.4		0	H	general GO
immune_response (366)	AMEF2.01	0.4		0	0	general GO
immune_response (366)	RORA2.01	0.4		H	0	general GO
immune_response (366)	NFAT.01	0.4			0	general GO
immune_response (366)	FREAC4.01	0.4		H	0	general GO
immune_response (366)	CDX2.01	0.4		H	0	general GO
immune_response (366)	MEF2.01	0.4		H	0	general GO
immune_response (366)	MMEF2.01	0.4		0	0	general GO
immune_response (366)	GATA2.02	0.4			H	general GO
immune_response (366)	GATA1.05	0.4		H	H	general GO
immune_response (366)	XFD3.01	0.4		H	0	general GO
immune_response (366)	IRF7.01	0.4		0	0	general GO
immune_response (366)	OCT1P.01	0.4		H	0	general GO
immune_response (366)	OCT.01	0.4			0	general GO
immune_response (366)	PDX1_G_SA/	0.4		H	0	general GO
immune_response (366)	GATA2.01	0.4			H	general GO
immune_response (366)	CDX1.01	0.3		H	0	general GO
immune_response (366)	VBP.01	0.3		H	0	general GO
immune_response (366)	NKX25.02	0.3		H	0	general GO
immune_response (366)	FREAC2.01	0.3		H	0	general GO
immune_response (366)	NMP4.01	0.3		0	0	general GO
immune_response (366)	OCT1.02	0.3		H	0	general GO

immune_response (366)	DLX3.01	0.3		H	0	general GO
immune_response (366)	HOXC13_01	0.3		H	0	general GO
immune_response (366)	HNF1.02	0.3		H	0	general GO
immune_response (366)	XFD1.01	0.3		H	0	general GO
immune_response (366)	GSH2_01	0.3	M	H	0	general GO
immune_response (366)	BARX2_01	0.3		H	0	general GO
immune_response (366)	ATBF1.01	0.3		H	0	general GO
immune_response (366)	HMEF2.01	0.3		H	0	general GO
immune_response (366)	GATA3.01	0.3		H	H	general GO
immune_response (366)	EVI1.04	0.3		H	0	general GO
immune_response (366)	EVI1.02	0.3		H	0	general GO
immune_response (366)	MEIS1_HOXA	0.3		H	H	general GO
immune_response (366)	PBX_HOXA9_	0.3		H	H	general GO
immune_response (366)	BRN3.02	0.3		H	H	general GO
immune_response (366)	FKHRL1.01	0.3		H	0	general GO
immune_response (366)	BRN2.03	0.3		H	0	general GO
immune_response (366)	XFD2.01	0.3		H	0	general GO
immune_response (366)	OC2.01	0.3		H	0	general GO
immune_response (366)	LMX1B.01	0.3		H	0	general GO
immune_response (366)	FREAC3.01	0.3		0	0	general GO
immune_response (366)	CART1.01	0.3		H	0	general GO
immune_response (366)	EN1.01	0.3		H	0	general GO
immune_response (366)	CABL.01	0.3		H	0	general GO
immune_response (366)	HNF1.03	0.3		H	0	general GO
immune_response (366)	EVI1.01	0.3		H	0	general GO
immune_response (366)	HNF6.01	0.3		H	0	general GO
immune_response (366)	SOX5.01	0.3		0	0	general GO
immune_response (366)	MSX.01	0.3	M	H	0	general GO
immune_response (366)	BRN3.01	0.3		H	0	general GO
immune_response (366)	ATATA.01	0.3		0	0	general GO
immune_response (366)	EVI1.06	0.3		H	0	general GO
immune_response (366)	MEF2.02	0.3		H	0	general GO
immune_response (366)	MEF2.03	0.3		H	0	general GO
immune_response (366)	HNF1.01	0.3		H	0	general GO
immune_response (366)	DLX1.01	0.3		H	0	general GO
immune_response (366)	MEF2.05	0.3		0	0	general GO
immune_response (366)	RSRFC4.02	0.3		H	0	general GO
immune_response (366)	MEL1_01	0.3			0	general GO
immune_response (366)	GATA3.02	0.3		H	H	general GO
immune_response (366)	MEF2.04	0.3		0	0	general GO
immune_response (366)	EVI1.05	0.3		H	0	general GO
immune_response (366)	TATA.01	0.3		0	H	general GO
immune_response (366)	RSRFC4.01	0.3			0	general GO
immune_response (366)	TATA.02	0.3		0	H	general GO
immune_response (366)	MYT1.02	0.3		H	0	general GO
immune_response (366)	PIT1.01	0.3		H	0	general GO
immune_response (366)	FREAC7.01	0.3		H	H	general GO
immune_response (366)	HFH1.01	0.3		H	0	general GO
immune_response (366)	HFH8.01	0.3		H	0	general GO
immune_response (366)	MYT1.01	0.3		H	0	general GO
immune_response (366)	OCT1.01	0.3		H	0	general GO
immune_response (366)	BRN5.01	0.3		H	0	general GO
immune_response (366)	HNF3B.01	0.3		H	0	general GO
immune_response (366)	HFH2.01	0.3		H	0	general GO
immune_response (366)	NKX31.01	0.2		H	0	general GO
immune_response (366)	BRN2.02	0.2		H	0	general GO
immune_response (366)	EVI1.03	0.2		H	H	general GO
immune_response (366)	S8.01	0.2	M	H	0	general GO
immune_response (366)	BRIGHT.01	0.2		H	0	general GO
immune_response (366)	OCT1.06	0.2		H	H	general GO
immune_response (366)	LHX3.01	0.1		H	0	general GO
immune_response (366)	poly_A	0		H	0	general GO
induction_of_apoptosis (86)	GFI1.01	0.4	0	H	M	general GO
induction_of_apoptosis (86)	IRF1.01	0.4		0	0	general GO

inflammatory_response (163)	NFKAPPAB.0	0.6		0	0	general GO
inflammatory_response (163)	NFKAPPAB.0	0.6		0	0	general GO
inflammatory_response (163)	NFKAPPAB6	0.6		0	0	general GO
inflammatory_response (163)	CREL.01	0.5		M	0	general GO
inflammatory_response (163)	NFKAPPAB.0	0.5		0	0	general GO
inflammatory_response (163)	HIVEP1_01	0.5		0	0	general GO
inflammatory_response (163)	AML1.01	0.5		H	0	general GO
inflammatory_response (163)	AP1FJ.01	0.5		0	0	general GO
inflammatory_response (163)	AP1.02	0.5		0	0	general GO
inflammatory_response (163)	LXRE.01	0.5		0	0	general GO
inflammatory_response (163)	ETS2.01	0.5		0	0	general GO
inflammatory_response (163)	GRE.01	0.5		0	0	general GO
inflammatory_response (163)	PPARA.01	0.5	M	H	0	general GO
inflammatory_response (163)	SRF.03	0.5	H	M	0	general GO
inflammatory_response (163)	ARE.01	0.5		0	0	general GO
inflammatory_response (163)	GKLF_02	0.5	M	H	0	general GO
inflammatory_response (163)	TAACC.01	0.5		H	0	general GO
inflammatory_response (163)	STAT6.01	0.5		0	0	general GO
inflammatory_response (163)	CEBP.02	0.5		0	0	general GO
inflammatory_response (163)	CDPCR3.01	0.5	0		0	general GO
inflammatory_response (163)	COMP1.01	0.5		0	H	general GO
inflammatory_response (163)	DBP.01	0.5		0	0	general GO
inflammatory_response (163)	BARBIE.01	0.4		0	H	general GO
inflammatory_response (163)	GATA1.01	0.4		M	0	general GO
inflammatory_response (163)	MYT1L.01	0.4		H	0	general GO
inflammatory_response (163)	BCL6.02	0.4		H	0	general GO
inflammatory_response (163)	TCF11MAFG	0.4		0	0	general GO
inflammatory_response (163)	AP1.01	0.4		0	0	general GO
inflammatory_response (163)	ISL1.01	0.4	M	H	0	general GO
inflammatory_response (163)	ISRE.01	0.4		0	0	general GO
inflammatory_response (163)	IRF2.01	0.4		0	0	general GO
inflammatory_response (163)	IRF3.01	0.4		0	0	general GO
inflammatory_response (163)	SRF.01	0.4		M	0	general GO
inflammatory_response (163)	PRDM1.01	0.4		H	0	general GO
inflammatory_response (163)	HMGIY.01	0.4		H	0	general GO
inflammatory_response (163)	HBP1_01	0.4	H	0		general GO
inflammatory_response (163)	BRN4.01	0.4		0	0	general GO
inflammatory_response (163)	MTBF.01	0.4		H	0	general GO
inflammatory_response (163)	IRF1.01	0.4	M	H	0	general GO
inflammatory_response (163)	CEBPB.01	0.4		H	0	general GO
inflammatory_response (163)	AARE.01	0.4		0	0	general GO
inflammatory_response (163)	AIRE.01	0.4		H	0	general GO
inflammatory_response (163)	LTATA_01	0.4		0	0	general GO
inflammatory_response (163)	BRN2.01	0.4		0	0	general GO
inflammatory_response (163)	IRF4.01	0.4		H	0	general GO
inflammatory_response (163)	STAT5.01	0.4		0	H	general GO
inflammatory_response (163)	GATA1.03	0.4		H	0	general GO
inflammatory_response (163)	BCL6.01	0.4		H	0	general GO
inflammatory_response (163)	AMEF2.01	0.4		0	0	general GO
inflammatory_response (163)	RORA2.01	0.4		H	0	general GO
inflammatory_response (163)	NFAT.01	0.4		H	0	general GO
inflammatory_response (163)	FREAC4.01	0.4	M	H	0	general GO
inflammatory_response (163)	CDX2.01	0.4		H	0	general GO
inflammatory_response (163)	MEF2.01	0.4	H		0	general GO
inflammatory_response (163)	MMEF2.01	0.4		0	0	general GO
inflammatory_response (163)	SOX9.01	0.4	0		0	general GO
inflammatory_response (163)	GATA2.02	0.4	H	H	M	general GO
inflammatory_response (163)	XFD3.01	0.4		0	0	general GO
inflammatory_response (163)	IRF7.01	0.4		H	0	general GO
inflammatory_response (163)	OCT1P.01	0.4			0	general GO
inflammatory_response (163)	OCT.01	0.4	H		0	general GO
inflammatory_response (163)	PDX1_G_SA	0.4		0	0	general GO
inflammatory_response (163)	CDX1.01	0.3		M	0	general GO
inflammatory_response (163)	FREAC2.01	0.3		0	0	general GO

inflammatory_response (163)	OCT1.02	0.3		0	0	general GO
inflammatory_response (163)	DLX3.01	0.3			0	general GO
inflammatory_response (163)	HOXC13_01	0.3		H	0	general GO
inflammatory_response (163)	HNF1.02	0.3		0	0	general GO
inflammatory_response (163)	XFD1.01	0.3		H	0	general GO
inflammatory_response (163)	ATBF1.01	0.3		H	0	general GO
inflammatory_response (163)	GATA3.01	0.3		H	0	general GO
inflammatory_response (163)	PAX4.01	0.3		0	0	general GO
inflammatory_response (163)	EVI1.04	0.3		0	0	general GO
inflammatory_response (163)	BRN3.02	0.3		0	0	general GO
inflammatory_response (163)	BRN2.03	0.3		0	0	general GO
inflammatory_response (163)	XFD2.01	0.3		0	0	general GO
inflammatory_response (163)	OC2.01	0.3		0	M	general GO
inflammatory_response (163)	LMX1B.01	0.3		H	0	general GO
inflammatory_response (163)	CART1.01	0.3		M	0	general GO
inflammatory_response (163)	EN1.01	0.3		H	0	general GO
inflammatory_response (163)	HNF1.03	0.3		0	0	general GO
inflammatory_response (163)	EVI1.01	0.3		H	0	general GO
inflammatory_response (163)	HNF6.01	0.3		H	0	general GO
inflammatory_response (163)	BRN3.01	0.3		H	H	general GO
inflammatory_response (163)	ATATA.01	0.3		0	0	general GO
inflammatory_response (163)	MEF2.02	0.3		H	0	general GO
inflammatory_response (163)	MEF2.03	0.3		H	0	general GO
inflammatory_response (163)	HNF1.01	0.3		0	0	general GO
inflammatory_response (163)	MEF2.05	0.3		0	0	general GO
inflammatory_response (163)	RSRFC4.02	0.3		M	0	general GO
inflammatory_response (163)	MEL1_01	0.3		H	M	general GO
inflammatory_response (163)	GATA3.02	0.3		0	0	general GO
inflammatory_response (163)	MEF2.04	0.3		H	0	general GO
inflammatory_response (163)	TATA.01	0.3		0	0	general GO
inflammatory_response (163)	RSRFC4.01	0.3		M	0	general GO
inflammatory_response (163)	TATA.02	0.3		0	0	general GO
inflammatory_response (163)	MYT1.02	0.3		H	0	general GO
inflammatory_response (163)	PIT1.01	0.3		0	0	general GO
inflammatory_response (163)	FREAC7.01	0.3		H	0	general GO
inflammatory_response (163)	SATB1.01	0.3		M	0	general GO
inflammatory_response (163)	HFH1.01	0.3		H	0	general GO
inflammatory_response (163)	HFH8.01	0.3		H	0	general GO
inflammatory_response (163)	MYT1.01	0.3		H	0	general GO
inflammatory_response (163)	OCT1.01	0.3		H	0	general GO
inflammatory_response (163)	BRN5.01	0.3		0	0	general GO
inflammatory_response (163)	HNF3B.01	0.3		H	0	general GO
inflammatory_response (163)	HFH2.01	0.3	M	H	0	general GO
inflammatory_response (163)	BRN2.02	0.2		0	0	general GO
inflammatory_response (163)	EVI1.03	0.2		H	0	general GO
inflammatory_response (163)	BRIGHT.01	0.2		0	0	general GO
inflammatory_response (163)	OCT1.06	0.2			0	general GO
inflammatory_response (163)	LHX3.01	0.1		0	0	general GO
inflammatory_response (163)	poly_A	0		H	0	general GO
innate_immune_response (46)	GRE.01	0.5		0	0	general GO
innate_immune_response (46)	STAT.01	0.4		0	H	general GO
innate_immune_response (46)	ISRE.01	0.4		0	0	general GO
innate_immune_response (46)	IRF3.01	0.4		0	0	general GO
innate_immune_response (46)	PRDM1.01	0.4	M	H	0	general GO
innate_immune_response (46)	PSE_02	0.4		H	0	general GO
innate_immune_response (46)	BRN4.01	0.4		0	0	general GO
innate_immune_response (46)	CDX2.01	0.4		0	H	general GO
innate_immune_response (46)	MMEF2.01	0.4		0	0	general GO
innate_immune_response (46)	GATA1.05	0.4	H	M	0	general GO
innate_immune_response (46)	GATA2.01	0.4		M	0	general GO
innate_immune_response (46)	HNF1.02	0.3		0	H	general GO
innate_immune_response (46)	ATBF1.01	0.3		0	0	general GO
innate_immune_response (46)	HMEF2.01	0.3	H	M	0	general GO
innate_immune_response (46)	PAX4.01	0.3		0	0	general GO

innate_immune_response (46)	XFD2.01	0.3		0	H	general GO
innate_immune_response (46)	LMX1B.01	0.3		0	0	general GO
innate_immune_response (46)	EN1.01	0.3		0	0	general GO
innate_immune_response (46)	HNF1.03	0.3		0	0	general GO
innate_immune_response (46)	SRY.01	0.3	H	M	0	general GO
innate_immune_response (46)	BRN3.01	0.3		0	0	general GO
innate_immune_response (46)	MEF2.03	0.3		0	0	general GO
innate_immune_response (46)	HNF1.01	0.3		0	0	general GO
innate_immune_response (46)	RSRFC4.01	0.3		0	0	general GO
innate_immune_response (46)	PIT1.01	0.3		0	H	general GO
innate_immune_response (46)	HFH8.01	0.3		0	0	general GO
innate_immune_response (46)	OCT1.01	0.3		0	0	general GO
innate_immune_response (46)	OCT1.06	0.2	H	M	0	general GO
intracellular_protein_transport (191)	GC_rich	1		0	0	general GO
intracellular_protein_transport (191)	SP1.01	0.8		0	0	general GO
intracellular_protein_transport (191)	ZF9.01	0.7		0	0	general GO
intracellular_protein_transport (191)	NRF1_01	0.7		H	0	general GO
intracellular_protein_transport (191)	EGR2.01	0.7	H	M	0	general GO
intracellular_protein_transport (191)	GC.01	0.7		0	0	general GO
intracellular_protein_transport (191)	ATF6.02	0.6		0	0	general GO
intracellular_protein_transport (191)	NRSE.01	0.6		0	0	general GO
intracellular_protein_transport (191)	ATF.01	0.6	H	M	0	general GO
intracellular_protein_transport (191)	XBP1.01	0.5		M	H	general GO
lipid_biosynthesis (54)	CAAT.01	0.5		0	0	general GO
lipid_biosynthesis (54)	NFY.02	0.5		0	0	general GO
lipid_catabolism (41)	HNF6.01	0.3	M	H	0	general GO
lipid_transport (41)	RORA1.01	0.4		0	0	general GO
mitosis (86)	NFY.01	0.5		H	0	general GO
mitosis (86)	CAAT.01	0.5		0	0	general GO
mitosis (86)	NFY.02	0.5		0	0	general GO
mitosis (86)	NFY.03	0.5		0	0	general GO
mitosis (86)	CHR.01	0.4		0	0	general GO
morphogenesis (104)	GC_rich	1	0	0		general GO
morphogenesis (104)	AP2.01	0.7	0	H		general GO
morphogenesis (104)	ZF5.01	0.7	0	0		general GO
morphogenesis (104)	HAND2_E12.	0.7	M	H	0	general GO
morphogenesis (104)	EGR1.02	0.7	H		H	general GO
mRNA_processing (148)	E2F.02	0.6		H	0	general GO
mRNA_processing (148)	NFY.01	0.5	0		0	general GO
muscle_contraction (78)	MYOD.02	0.6	M	0	H	general GO
muscle_development (107)	INSM1_01	0.6		0	0	general GO
muscle_development (107)	HNF4.01	0.5	0		0	general GO
muscle_development (107)	SRF.02	0.5		0	0	general GO
muscle_development (107)	MTATA.01	0.5		0	0	general GO
muscle_development (107)	SRF.03	0.5		0	0	general GO
muscle_development (107)	SRF.01	0.4		0	0	general GO
muscle_development (107)	AMEF2.01	0.4		0	0	general GO
muscle_development (107)	MEF2.02	0.3		0	0	general GO
muscle_development (107)	MEF2.03	0.3		0	0	general GO
muscle_development (107)	RSRFC4.02	0.3		0	0	general GO
muscle_development (107)	RSRFC4.01	0.3		0	0	general GO
negative_regulation_of_cell_proliferation (130)	PPARA.01	0.5	0		0	general GO
nervous_system_development (225)	GC_rich	1	H	H		general GO
nervous_system_development (225)	CKROX_01	0.7		H		general GO
nervous_system_development (225)	PLAG1_01	0.6	H		H	general GO
nervous_system_development (225)	GAGA.01	0.6		H	0	general GO
nuclear_mRNA_splicing_via_spliceosome (94)	NRF2.01	0.6		0	0	general GO
nuclear_mRNA_splicing_via_spliceosome (94)	ATF.02	0.5		H	H	general GO
nucleosome_assembly (71)	NFY.01	0.5		H	0	general GO
nucleosome_assembly (71)	NFY.02	0.5		H	0	general GO
nucleosome_assembly (71)	NFY.03	0.5		0	0	general GO
nucleosome_assembly (71)	TATA.01	0.3		H	0	general GO
organ_morphogenesis (71)	poly_C	1	H	M	0	general GO
organ_morphogenesis (71)	WT1.01	0.8	0		H	general GO

organ_morphogenesis (71)	INSM1_01	0.6	H	H	M	general GO
organ_morphogenesis (71)	GAGA.01	0.6	H	M	0	general GO
positive_regulation_of_I_kappaB_kinase_NF_kappaB_cascade (56)	ETS1.01	0.5	H	0	M	general GO
positive_regulation_of_I_kappaB_kinase_NF_kappaB_cascade (56)	IRF3.01	0.4		0	0	general GO
potassium_ion_transport (108)	WT1.01	0.8	0		0	general GO
potassium_ion_transport (108)	EGR1.01	0.7	H	M	0	general GO
potassium_ion_transport (108)	PAX9.01	0.6	0	0		general GO
pregnancy (41)	PDX1_Gabi	0.4		0	0	general GO
pregnancy (41)	XFD3.01	0.4		0	H	general GO
pregnancy (41)	BRN3.01	0.3		0	0	general GO
protein_amino_acid_dephosphorylation (96)	MUSCLE_INI	0.6		0	0	general GO
protein_amino_acid_phosphorylation (373)	SP1.01	0.8	H		0	general GO
protein_amino_acid_phosphorylation (373)	MTF-1.01	0.7	H	0	M	general GO
protein_amino_acid_phosphorylation (373)	ZNF35_01	0.5	0	0		general GO
protein_amino_acid_phosphorylation (373)	ATF.02	0.5	0		0	general GO
protein_biosynthesis (197)	GABP.01	0.6		0	0	general GO
protein_biosynthesis (197)	CDE.01	0.6		H	H	general GO
protein_biosynthesis (197)	WHN.01	0.6			0	general GO
protein_biosynthesis (197)	NRF2.01	0.6		H	0	general GO
protein_biosynthesis (197)	ATF.01	0.6	H	M	0	general GO
protein_biosynthesis (197)	CETS1P54.0	0.6		0	0	general GO
protein_biosynthesis (197)	YY1.01	0.6		0	0	general GO
protein_biosynthesis (197)	ELK1.02	0.5			0	general GO
protein_biosynthesis (197)	E2F.03	0.5	H		0	general GO
protein_biosynthesis (197)	ELF2.01	0.5		0	0	general GO
protein_biosynthesis (197)	FLI.01	0.5		0	0	general GO
protein_biosynthesis (197)	ELK1.01	0.5		0	0	general GO
protein_biosynthesis (197)	E2F.01	0.5		0	0	general GO
protein_biosynthesis (197)	ISRE.01	0.4		0	0	general GO
protein_biosynthesis (197)	HMGIY.01	0.4	H	M	0	general GO
protein_biosynthesis (197)	HOXB9_01	0.4	H	M	0	general GO
protein_folding (149)	FLI.01	0.5		H	0	general GO
protein_folding (149)	VMYB.03	0.4		0	0	general GO
protein_kinase_cascade (43)	E47.01	0.6		0	0	general GO
protein_targeting (37)	NRF2.01	0.6		0	0	general GO
protein_transport (254)	GC_rich	1		0	0	general GO
protein_transport (254)	ZF5.01	0.7		0	0	general GO
protein_transport (254)	NRF1_01	0.7		0	0	general GO
protein_transport (254)	EGR1.02	0.7		0	0	general GO
protein_transport (254)	MUSCLE_INI	0.6		0	0	general GO
protein_transport (254)	GABP.01	0.6		0	0	general GO
protein_transport (254)	WHN.01	0.6		H	0	general GO
protein_transport (254)	NRF2.01	0.6		H	0	general GO
protein_transport (254)	CREB.02	0.6		H	0	general GO
protein_transport (254)	XBP1.01	0.5		0	H	general GO
protein_ubiquitination (118)	MIF1.01	0.5	H	0	M	general GO
proteolysis (297)	NRL.01	0.5	H	0	M	general GO
proteolysis (297)	TH1E47.01	0.5	H	M	0	general GO
proteolysis (297)	AREB6.01	0.5		0	H	general GO
proteolysis (297)	COUP.01	0.5		0	0	general GO
proteolysis (297)	AREB6.02	0.5		0	0	general GO
proteolysis (297)	AP1.01	0.4		0	0	general GO
proteolysis (297)	CEBPB.01	0.4		0	0	general GO
proteolysis (297)	AIRE.01	0.4	0	M	H	general GO
proteolysis (297)	GATA1.03	0.4		0	0	general GO
proteolysis (297)	XFD3.01	0.4		0	0	general GO
proteolysis (297)	ATBF1.01	0.3		0	0	general GO
proteolysis (297)	HMEF2.01	0.3	M	0	H	general GO
proteolysis (297)	XFD2.01	0.3	M	H	0	general GO
proteolysis (297)	HNF1.03	0.3		0	0	general GO
proteolysis (297)	HNF1.01	0.3		0	0	general GO
proteolysis (297)	HFH1.01	0.3		0	0	general GO
proteolysis (297)	HFH8.01	0.3		H	0	general GO
proteolysis (297)	HNF3B.01	0.3		H	0	general GO

proteolysis (297)	HFH2.01	0.3		0	0	general GO
regulation_of_apoptosis (59)	IRF3.01	0.4		0	0	general GO
regulation_of_cyclin_dependent_protein_kinase_activity (34)	NFY.01	0.5	0		0	general GO
regulation_of_progression_through_cell_cycle (217)	AHRARNT.02	0.6	0	M	H	general GO
regulation_of_progression_through_cell_cycle (217)	E2F.01	0.5	H		0	general GO
regulation_of_progression_through_cell_cycle (217)	HMEF2.01	0.3	M	0	H	general GO
response_to_DNA_damage_stimulus (117)	EGR1.01	0.7	H	H	M	general GO
response_to_DNA_damage_stimulus (117)	E2F.02	0.6		M	0	general GO
response_to_DNA_damage_stimulus (117)	E2F.03	0.5		0	M	general GO
response_to_DNA_damage_stimulus (117)	FLI.01	0.5		0	0	general GO
response_to_DNA_damage_stimulus (117)	E2F.01	0.5		M	0	general GO
response_to_virus (55)	NFKAPPAB.0	0.5		0	0	general GO
response_to_virus (55)	HIVEP1_01	0.5		0	0	general GO
response_to_virus (55)	ISRE.01	0.4		M	0	general GO
response_to_virus (55)	IRF2.01	0.4		H	0	general GO
response_to_virus (55)	IRF3.01	0.4		0	0	general GO
response_to_virus (55)	PRDM1.01	0.4	M	H	0	general GO
response_to_virus (55)	HBP1_01	0.4		0	0	general GO
response_to_virus (55)	IRF1.01	0.4			H	general GO
response_to_virus (55)	IRF4.01	0.4		H	0	general GO
response_to_virus (55)	GATA1.05	0.4	H		0	general GO
response_to_virus (55)	IRF7.01	0.4		0	0	general GO
response_to_virus (55)	TATA.01	0.3		H	0	general GO
RNA_splicing (61)	NRF2.01	0.6		0	0	general GO
sensory_perception (201)	NFKAPPAB.0	0.6		0	0	general GO
sensory_perception (201)	HIVEP1_01	0.5		0	0	general GO
sensory_perception (201)	NBRE.01	0.5		0	0	general GO
sensory_perception (201)	NFE2.01	0.5		0	0	general GO
sensory_perception (201)	IK2.01	0.5		0	0	general GO
sensory_perception (201)	LMO2COM.0	0.5		H	0	general GO
sensory_perception (201)	SRF.03	0.5		0	0	general GO
sensory_perception (201)	MIT.01	0.5		0	0	general GO
sensory_perception (201)	MEL1_03	0.5		H	0	general GO
sensory_perception (201)	DBP.01	0.5		H	0	general GO
sensory_perception (201)	PAX8.01	0.4		H	0	general GO
sensory_perception (201)	GATA1.01	0.4		H	0	general GO
sensory_perception (201)	AP1.01	0.4		0	0	general GO
sensory_perception (201)	SRF.01	0.4		0	0	general GO
sensory_perception (201)	RORA1.01	0.4		H	0	general GO
sensory_perception (201)	GFI1.01	0.4		H	0	general GO
sensory_perception (201)	MTBF.01	0.4		0	0	general GO
sensory_perception (201)	GATA1.04	0.4		H	0	general GO
sensory_perception (201)	CRX.01	0.4		H	0	general GO
sensory_perception (201)	AARE.01	0.4	H		0	general GO
sensory_perception (201)	LTATA_01	0.4		0	0	general GO
sensory_perception (201)	OTX2.01	0.4		H	0	general GO
sensory_perception (201)	GATA1.03	0.4		H	0	general GO
sensory_perception (201)	BCL6.01	0.4	H	M	0	general GO
sensory_perception (201)	AMEF2.01	0.4	H		M	general GO
sensory_perception (201)	CDX2.01	0.4		H	0	general GO
sensory_perception (201)	GATA2.02	0.4		H	0	general GO
sensory_perception (201)	XFD3.01	0.4		0	0	general GO
sensory_perception (201)	OCT1P.01	0.4		H	0	general GO
sensory_perception (201)	SIX3.01	0.4		H	0	general GO
sensory_perception (201)	PDX1_G_SA	0.4		H	H	general GO
sensory_perception (201)	GATA2.01	0.4		H	H	general GO
sensory_perception (201)	CDX1.01	0.3		H	0	general GO
sensory_perception (201)	NKX25.02	0.3		H	0	general GO
sensory_perception (201)	FREAC2.01	0.3	0		H	general GO
sensory_perception (201)	HMEF2.01	0.3		H	0	general GO
sensory_perception (201)	GATA3.01	0.3		H	H	general GO
sensory_perception (201)	BRN3.02	0.3		H	H	general GO
sensory_perception (201)	FKHRL1.01	0.3	H		H	general GO
sensory_perception (201)	XFD2.01	0.3			H	general GO

sensory_perception (201)	OC2.01	0.3		H	0	general GO
sensory_perception (201)	EN1.01	0.3		H	H	general GO
sensory_perception (201)	MEF2.02	0.3		H	0	general GO
sensory_perception (201)	MEF2.05	0.3		H	0	general GO
sensory_perception (201)	CDP.02	0.3		H	0	general GO
sensory_perception (201)	MEL1_01	0.3		H	0	general GO
sensory_perception (201)	GATA3.02	0.3		H	0	general GO
sensory_perception (201)	EVI1.05	0.3		H	0	general GO
sensory_perception (201)	TATA.01	0.3		0	0	general GO
sensory_perception (201)	EVI1.03	0.2		H	0	general GO
sensory_perception (201)	BRIGHT.01	0.2		H	0	general GO
sensory_perception_of_smell (40)	NFE2L2.01	0.5		0	0	general GO
sensory_perception_of_smell (40)	GFI1.01	0.4	M	H	0	general GO
signal_transduction (1189)	E47.01	0.6	0	H	M	general GO
signal_transduction (1189)	NFKAPPAB.0	0.5	H	M	0	general GO
signal_transduction (1189)	HNF4.01	0.5	0	0		general GO
signal_transduction (1189)	HIVEP1_01	0.5		M	0	general GO
signal_transduction (1189)	AML1.01	0.5		0	0	general GO
signal_transduction (1189)	MEL1_02	0.5		0	0	general GO
signal_transduction (1189)	AP1.01	0.4		0	0	general GO
signal_transduction (1189)	AARE.01	0.4		0	0	general GO
signal_transduction (1189)	EVI1.02	0.3		0	0	general GO
signal_transduction (1189)	EVI1.01	0.3		0	0	general GO
signal_transduction (1189)	poly_A	0		0	0	general GO
skeletal_development (83)	AG_rich_codi	0.5		0	0	general GO
small_GTPase_mediated_signal_transduction (124)	GC_rich	1		H	0	general GO
small_GTPase_mediated_signal_transduction (124)	SP1.01	0.8		H	0	general GO
small_GTPase_mediated_signal_transduction (124)	ZF5.01	0.7		0	0	general GO
small_GTPase_mediated_signal_transduction (124)	ZF9.01	0.7		H	0	general GO
small_GTPase_mediated_signal_transduction (124)	EGR3.01	0.7	H	0	M	general GO
small_GTPase_mediated_signal_transduction (124)	NRF1_01	0.7		H	0	general GO
small_GTPase_mediated_signal_transduction (124)	CKROX_01	0.7		H	0	general GO
small_GTPase_mediated_signal_transduction (124)	EGR1.02	0.7		H	0	general GO
small_GTPase_mediated_signal_transduction (124)	GC.01	0.7		H	0	general GO
small_GTPase_mediated_signal_transduction (124)	ATF6.01	0.6	H	0	M	general GO
small_GTPase_mediated_signal_transduction (124)	PLAG1_01	0.6	H	H	M	general GO
small_GTPase_mediated_signal_transduction (124)	EBVR.01	0.6		0	0	general GO
sodium_ion_transport (75)	FXRE.01	0.5	0		0	general GO
sodium_ion_transport (75)	HNF1.02	0.3		0	0	general GO
sodium_ion_transport (75)	HNF1.03	0.3		0	0	general GO
sodium_ion_transport (75)	HNF1.01	0.3		0	0	general GO
steroid_biosynthesis (42)	NFY.01	0.5		0	0	general GO
steroid_biosynthesis (42)	CAAT.01	0.5		0	0	general GO
steroid_biosynthesis (42)	NFY.02	0.5		0	0	general GO
steroid_biosynthesis (42)	NFY.03	0.5		0	0	general GO
steroid_biosynthesis (42)	GATA1.03	0.4	0	H	M	general GO
steroid_metabolism (54)	HNF3B.01	0.3		0	0	general GO
synaptic_transmission (153)	NRSF.01	0.6	M	0	H	general GO
synaptic_transmission (153)	NRSE.01	0.6		0	H	general GO
synaptic_transmission (153)	GAGA.01	0.6	H	M	H	general GO
transmembrane_receptor_tyrosine_kinase_signaling (65)	E47.01	0.6	0	0		general GO
transmembrane_receptor_tyrosine_kinase_signaling (65)	GAGA.01	0.6		0	M	general GO
transport (940)	ERR_01	0.5		0	H	general GO
transport (940)	OTX2.01	0.4	M	0	H	general GO
transport (940)	HNF1.02	0.3		0	0	general GO
transport (940)	HNF1.03	0.3		0	0	general GO
transport (940)	HNF1.01	0.3		0	0	general GO
ubiquitin_cycle (154)	GC_rich	1		0	0	general GO
ubiquitin_cycle (154)	HES1.02	0.8		0	0	general GO
ubiquitin_cycle (154)	ZF5.01	0.7		0	0	general GO
ubiquitin_cycle (154)	HES1.01	0.6		0	0	general GO
ubiquitin_cycle (154)	FLI.01	0.5		0	0	general GO
ubiquitin_cycle (154)	VMYB.05	0.4	0		H	general GO
ubiquitin_dependent_protein_catabolism (72)	IRF2.01	0.4		0	0	general GO

vesicle_mediated_transport (51)	SP1.01	0.8		0	0	general GO
visual_perception (142)	VMAF.01	0.5		0	0	general GO
visual_perception (142)	TBX5.01	0.5		0	0	general GO
visual_perception (142)	HOX_PBX_01	0.5		0	0	general GO
visual_perception (142)	GFI1.01	0.4		0	0	general GO
visual_perception (142)	CRX.01	0.4		0	0	general GO
visual_perception (142)	OTX2.01	0.4		H	0	general GO
visual_perception (142)	PDX1.01	0.4		0	0	general GO
visual_perception (142)	SIX3.01	0.4		H	0	general GO
visual_perception (142)	OC2.01	0.3	M	H	H	general GO
visual_perception (142)	GATA3.02	0.3		0	0	general GO
visual_perception (142)	HFH1.01	0.3	M	H	0	general GO
Wnt_receptor_signaling_pathway (66)	GC_rich	1	H	H		general GO
Wnt_receptor_signaling_pathway (66)	poly_C	1		H	H	general GO
Wnt_receptor_signaling_pathway (66)	SP1.01	0.8	H	0		general GO
Wnt_receptor_signaling_pathway (66)	WT1.01	0.8		0	H	general GO
Wnt_receptor_signaling_pathway (66)	MAZR.01	0.8		0	0	general GO
Wnt_receptor_signaling_pathway (66)	ZNF202.01	0.7		0	0	general GO
Wnt_receptor_signaling_pathway (66)	AP2.01	0.7	H	H		general GO
Wnt_receptor_signaling_pathway (66)	ZF5.01	0.7		H		general GO
Wnt_receptor_signaling_pathway (66)	ZF9.01	0.7	0	0		general GO
Wnt_receptor_signaling_pathway (66)	EGR1.02	0.7	H	H		general GO
Wnt_receptor_signaling_pathway (66)	EGR1.01	0.7	H		H	general GO
Wnt_receptor_signaling_pathway (66)	PLAG1_01	0.6		H	0	general GO
Wnt_receptor_signaling_pathway (66)	CDE.01	0.6	H	M	0	general GO
Wnt_receptor_signaling_pathway (66)	PAX9.01	0.6	M	0	H	general GO
Wnt_receptor_signaling_pathway (66)	NRSE.01	0.6	0	M	H	general GO
Wnt_receptor_signaling_pathway (66)	PAX5.01	0.6	0		M	general GO

700bp Window (with overlap)

GO group	Motifs	motif GC	-199 - 500	-99 - 600	1 - 700	101 - 800	201 - 900	301 - 1000	GO-classes
transcription (930)	GC_rich	0.9915							transcription GO
regulation_of_transcription_DNA_dependent	GC_rich	0.9915							transcription GO
regulation_of_transcription (266)	GC_rich	0.9915							transcription GO
development (530)	GC_rich	0.9915	0						transcription GO
transcription_RNA_polyII_promoter (190)	poly_C	0.989	0	H	0	H		H	transcription GO
transcription (930)	poly_C	0.989							transcription GO
regulation_of_transcription_DNA_dependent	poly_C	0.989							transcription GO
regulation_of_transcription (266)	poly_C	0.989							transcription GO
development (530)	poly_C	0.989							transcription GO
transcription (930)	SP1.01	0.778846154		H	H				transcription GO
regulation_of_transcription_DNA_dependent	SP1.01	0.778846154	H	H	H				transcription GO
regulation_of_transcription (266)	SP1.01	0.778846154	0	0	H			M	transcription GO
development (530)	SP1.01	0.778846154	H	H	H				transcription GO
transcription (930)	WT1.01	0.770561018							transcription GO
regulation_of_transcription_DNA_dependent	WT1.01	0.770561018							transcription GO
regulation_of_transcription (266)	WT1.01	0.770561018							transcription GO
development (530)	WT1.01	0.770561018	H						transcription GO
transcription (930)	HES1.02	0.763263941	M	M					transcription GO
regulation_of_transcription_DNA_dependent	HES1.02	0.763263941	M	M	M				transcription GO
transcription (930)	MAZR.01	0.762075134							transcription GO
regulation_of_transcription_DNA_dependent	MAZR.01	0.762075134							transcription GO
regulation_of_transcription (266)	MAZR.01	0.762075134							transcription GO
development (530)	MAZR.01	0.762075134							transcription GO
transcription (930)	ZNF202.01	0.732919255							transcription GO
regulation_of_transcription_DNA_dependent	ZNF202.01	0.732919255							transcription GO
regulation_of_transcription (266)	ZNF202.01	0.732919255							transcription GO
development (530)	ZNF202.01	0.732919255							transcription GO
transcription (930)	AP2.01	0.725490196							transcription GO
regulation_of_transcription_DNA_dependent	AP2.01	0.725490196							transcription GO
regulation_of_transcription (266)	AP2.01	0.725490196							transcription GO
development (530)	AP2.01	0.725490196	H						transcription GO
transcription (930)	ZF5.01	0.719485294							transcription GO
regulation_of_transcription_DNA_dependent	ZF5.01	0.719485294							transcription GO
regulation_of_transcription (266)	ZF5.01	0.719485294			H			H	transcription GO
transcription (930)	ZF9.01	0.717844061							transcription GO
regulation_of_transcription_DNA_dependent	ZF9.01	0.717844061	H	H	0				transcription GO
regulation_of_transcription (266)	ZF9.01	0.717844061	0	0	0	M		M	transcription GO
development (530)	ZF9.01	0.717844061	0	0	0	M			transcription GO
transcription (930)	EGR3.01	0.709376693	H		H				transcription GO
regulation_of_transcription_DNA_dependent	EGR3.01	0.709376693			H				transcription GO
transcription (930)	NRF1_01	0.708333333	H	H					transcription GO
regulation_of_transcription_DNA_dependent	NRF1_01	0.708333333		H					transcription GO
transcription (930)	MYCMAX.03	0.701731602	0	0	M				transcription GO
regulation_of_transcription_DNA_dependent	MYCMAX.03	0.701731602	0	M	M			H	transcription GO
transcription (930)	NFKAPPAB5	0.7	0	H	H	H	H		transcription GO
development (530)	NFKAPPAB5	0.7	0	H					transcription GO
development (530)	HAND2_E12.	0.6957522						H	transcription GO
transcription (930)	ZBP89.01	0.695601852							transcription GO
regulation_of_transcription_DNA_dependent	ZBP89.01	0.695601852							transcription GO
regulation_of_transcription (266)	ZBP89.01	0.695601852							transcription GO
development (530)	ZBP89.01	0.695601852							transcription GO

transcription (930)	HELT.01	0.691910867	H	H	H				transcription GO
regulation_of_transcription_DNA_dependent	HELT.01	0.691910867	H	H	H				transcription GO
transcription (930)	NGFIC.01	0.685824218			H				transcription GO
regulation_of_transcription_DNA_dependent	NGFIC.01	0.685824218							transcription GO
development (530)	NGFIC.01	0.685824218	M	M		M	M	M	transcription GO
transcription (930)	CKROX_01	0.683333333							transcription GO
regulation_of_transcription_DNA_dependent	CKROX_01	0.683333333							transcription GO
regulation_of_transcription (266)	CKROX_01	0.683333333							transcription GO
development (530)	CKROX_01	0.683333333							transcription GO
transcription (930)	EGR1.02	0.683035714							transcription GO
regulation_of_transcription_DNA_dependent	EGR1.02	0.683035714							transcription GO
regulation_of_transcription (266)	EGR1.02	0.683035714			M				transcription GO
development (530)	EGR1.02	0.683035714	0	0	0				transcription GO
transcription (930)	HIC1_01	0.681615155	0	0	0	0		M	transcription GO
regulation_of_transcription_DNA_dependent	HIC1_01	0.681615155	0	0	0	M			transcription GO
regulation_of_transcription (266)	HIC1_01	0.681615155	0	0	0	0	M		transcription GO
development (530)	HIC1_01	0.681615155	M	M	M				transcription GO
regulation_of_transcription_DNA_dependent	BKLF.01	0.681578947	0	0	0	H			transcription GO
development (530)	BKLF.01	0.681578947	0	H	H	H		H	transcription GO
transcription (930)	EGR2.01	0.680134551			M	M			transcription GO
regulation_of_transcription_DNA_dependent	EGR2.01	0.680134551							transcription GO
transcription (930)	GC.01	0.679856115	H	0	0			M	transcription GO
regulation_of_transcription_DNA_dependent	GC.01	0.679856115	H	0	0			M	transcription GO
regulation_of_transcription (266)	GC.01	0.679856115	0	0	0	M	H	0	transcription GO
development (530)	GC.01	0.679856115	H	H	H	M		M	transcription GO
transcription (930)	MZF1.01	0.678571429	M		M			M	transcription GO
regulation_of_transcription_DNA_dependent	MZF1.01	0.678571429			M				transcription GO
regulation_of_transcription (266)	MZF1.01	0.678571429							transcription GO
development (530)	MZF1.01	0.678571429							transcription GO
transcription (930)	EGR1.01	0.67232125	H						transcription GO
regulation_of_transcription_DNA_dependent	EGR1.01	0.67232125							transcription GO
transcription (930)	MTF-1.01	0.653230769	0	0	0		H	0	transcription GO
regulation_of_transcription_DNA_dependent	MTF-1.01	0.653230769	M	0				M	transcription GO
development (530)	MTF-1.01	0.653230769	0	0	0	H	0	M	transcription GO
transcription (930)	MAZ.01	0.651515152							transcription GO
regulation_of_transcription_DNA_dependent	MAZ.01	0.651515152							transcription GO
regulation_of_transcription (266)	MAZ.01	0.651515152							transcription GO
development (530)	MAZ.01	0.651515152	H	H	H				transcription GO
transcription (930)	ZIC2_01	0.648148148	0	M	0	M		M	transcription GO
regulation_of_transcription_DNA_dependent	ZIC2_01	0.648148148	M	M	M	M	M		transcription GO
regulation_of_transcription (266)	ZIC2_01	0.648148148	M	M	0	H			transcription GO
development (530)	ZIC2_01	0.648148148	0	0	H				transcription GO
transcription (930)	ATF6.01	0.639053254	M	M			H	H	transcription GO
regulation_of_transcription_DNA_dependent	ATF6.01	0.639053254	M	M					transcription GO
transcription (930)	PLAG1_01	0.63261693							transcription GO
regulation_of_transcription_DNA_dependent	PLAG1_01	0.63261693							transcription GO
regulation_of_transcription (266)	PLAG1_01	0.63261693							transcription GO
development (530)	PLAG1_01	0.63261693							transcription GO
regulation_of_transcription_DNA_dependent	INSM1_01	0.629180602	0	0	M	M	M	H	transcription GO
development (530)	INSM1_01	0.629180602	H	H	H				transcription GO
regulation_of_transcription_DNA_dependent	EBVR.01	0.627594628	H	H	H	0		M	transcription GO
transcription (930)	AHRARNT.02	0.624493927					H		transcription GO
regulation_of_transcription_DNA_dependent	AHRARNT.02	0.624493927	0	0	H				transcription GO
transcription (930)	HES1.01	0.622222222							transcription GO
regulation_of_transcription_DNA_dependent	HES1.01	0.622222222							transcription GO
regulation_of_transcription (266)	HES1.01	0.622222222	0	H	0	0	M	0	transcription GO
transcription (930)	MUSCLE_INI	0.620813397						H	transcription GO
regulation_of_transcription_DNA_dependent	MUSCLE_INI	0.620813397	M						transcription GO
development (530)	MUSCLE_INI	0.620813397	H	H				H	transcription GO
transcription (930)	NMYC.01	0.615909091	0	0	0		0	0	transcription GO
regulation_of_transcription_DNA_dependent	NMYC.01	0.615909091	0	0	0		0	0	transcription GO
transcription (930)	HIF1.01	0.607692308	0	0	0		H	H	transcription GO
regulation_of_transcription_DNA_dependent	HIF1.01	0.607692308	0	0	0		H	H	transcription GO
transcription (930)	MUSCLE_INI	0.607655502	H		H	H		H	transcription GO

regulation_of_transcription_DNA_dependent	MUSCLE_INI	0.607655502	M						transcription GO
regulation_of_transcription_DNA_dependent	USF.03	0.6	0	0	0		0	0	transcription GO
regulation_of_transcription_DNA_dependent	ZNF76_143_(	0.6	0	H	H	H		H	transcription GO
development (530)	ZNF76_143_(	0.6	0	0	0	H		H	transcription GO
regulation_of_transcription_DNA_dependent	EKLF.01	0.595779221	0	0	0			M	transcription GO
regulation_of_transcription_DNA_dependent	RREB1.01	0.595238095	H	H	H	H		0	transcription GO
transcription (930)	CDE.01	0.595238095							transcription GO
regulation_of_transcription_DNA_dependent	CDE.01	0.595238095							transcription GO
regulation_of_transcription (266)	CDE.01	0.595238095	0	H		H		0	transcription GO
development (530)	CDE.01	0.595238095	0	0	0	0	M		transcription GO
regulation_of_transcription (266)	ZBRK1_01	0.594024416	0	M	0	0	H	H	transcription GO
regulation_of_transcription_DNA_dependent	AHR.01	0.594017094	H	H	H	H			transcription GO
regulation_of_transcription (266)	AHR.01	0.594017094	0	0	0	0	0		transcription GO
transcription (930)	E2F.02	0.59375	H	H	H	0	0		transcription GO
regulation_of_transcription_DNA_dependent	E2F.02	0.59375		H	H				transcription GO
regulation_of_transcription (266)	E2F.02	0.59375	0	0	0	0	0		transcription GO
transcription (930)	WHN.01	0.592307692	0	0	0	M	M		transcription GO
transcription (930)	PAX9.01	0.574305556	0	0			H	H	transcription GO
regulation_of_transcription_DNA_dependent	PAX9.01	0.574305556	0	0	M		H		transcription GO
development (530)	PAX9.01	0.574305556	0	0	0	0	H	M	transcription GO
transcription (930)	PAX5.01	0.568181818	H	H	H	H	H	M	transcription GO
regulation_of_transcription_DNA_dependent	PAX5.01	0.568181818	0	0	0			M	transcription GO
regulation_of_transcription (266)	PAX5.01	0.568181818	0	0	0		0	M	transcription GO
development (530)	PAX5.01	0.568181818	0	0	H	H	H		transcription GO
transcription (930)	PAX5.03	0.564251208	H	0	M	M	0	0	transcription GO
regulation_of_transcription (266)	VDR_RXR.02	0.562962963	H	H	H	H	H		transcription GO
development (530)	VDR_RXR.02	0.562962963	H	H	H		H	H	transcription GO
transcription (930)	GAGA.01	0.562878788				M			transcription GO
regulation_of_transcription_DNA_dependent	GAGA.01	0.562878788							transcription GO
regulation_of_transcription (266)	GAGA.01	0.562878788				H			transcription GO
development (530)	GAGA.01	0.562878788							transcription GO
development (530)	VDR_RXR.01	0.561111111	H	H	H	H			transcription GO
regulation_of_transcription_DNA_dependent	TAXCREB.02	0.555555556	0	0	M	0	H	H	transcription GO
regulation_of_transcription_DNA_dependent	E2.02	0.546195652	0	0	0	0	H		transcription GO
development (530)	ZNF35_01	0.545808967	0	0	0	0	H		transcription GO
development (530)	OLF1.01	0.545454545	0	0	0	H		H	transcription GO
regulation_of_transcription_DNA_dependent	CREB.03	0.543859649	0	0	0	0		M	transcription GO
transcription (930)	E2F.03	0.538461538	0	0	0	H			transcription GO
regulation_of_transcription_DNA_dependent	E2F.03	0.538461538	0	0	M	H			transcription GO
transcription (930)	NFY.01	0.518088822	H	H	H		H	0	transcription GO
regulation_of_transcription_DNA_dependent	NFY.01	0.518088822						M	transcription GO
regulation_of_transcription (266)	NFY.01	0.518088822						0	transcription GO
transcription (930)	AG_rich_codi	0.5						H	transcription GO
regulation_of_transcription_DNA_dependent	AG_rich_codi	0.5							transcription GO
regulation_of_transcription (266)	AG_rich_codi	0.5							transcription GO
development (530)	AG_rich_codi	0.5				H	H	H	transcription GO
regulation_of_transcription_DNA_dependent	SRF.02	0.494285714	H	H		H	H	0	transcription GO
transcription (930)	GKLF.01	0.491428571			H	H	H	0	transcription GO
regulation_of_transcription_DNA_dependent	GKLF.01	0.491428571			H	H	H	H	transcription GO
regulation_of_transcription (266)	GKLF.01	0.491428571			H				transcription GO
development (530)	GKLF.01	0.491428571	H		H		H		transcription GO
transcription (930)	CAAT.01	0.489080752				0	0	0	transcription GO
regulation_of_transcription_DNA_dependent	CAAT.01	0.489080752					M	0	transcription GO
regulation_of_transcription (266)	CAAT.01	0.489080752			M	M	0	0	transcription GO
regulation_of_transcription_DNA_dependent	NFY.02	0.477272727				M	0	0	transcription GO
regulation_of_transcription (266)	NFY.02	0.477272727		H	H		0	0	transcription GO
development (530)	MTATA.01	0.470588235			H	0	0	0	transcription GO
regulation_of_transcription_DNA_dependent	PBX1_MEIS1	0.462145969	H	0	0	M	M	0	transcription GO
regulation_of_transcription (266)	PBX1_MEIS1	0.462145969			M	M	M	M	transcription GO
regulation_of_transcription_DNA_dependent	PBX1_MEIS1	0.440247253	H		0	M	M	0	transcription GO
regulation_of_transcription (266)	PBX1_MEIS1	0.440247253				M	M	0	transcription GO
Wnt_receptor_signaling_pathway (66)	GC_rich	0.9915		H					general GO
ubiquitin_cycle (154)	GC_rich	0.9915	H		H	0	0	0	general GO
transcription_RNA_polyII_promoter (190)	GC_rich	0.9915		0					general GO

small_GTPase_mediated_signal_transduction	GC_rich	0.9915				H	H	0	general GO
regulation_of_transcription_from_RNA_polymera	GC_rich	0.9915		H	H	0	0	0	general GO
regulation_of_progression_through_cell_cycle	GC_rich	0.9915	0	M	0	M	H	H	general GO
protein_transport (254)	GC_rich	0.9915		H	H	0	0	0	general GO
nervous_system_development (225)	GC_rich	0.9915	0	H		H	0	H	general GO
intracellular_protein_transport (191)	GC_rich	0.9915		H	H	0	0	0	general GO
Wnt_receptor_signaling_pathway (66)	poly_C	0.989			H	H	H	H	general GO
regulation_of_transcription_from_RNA_polymera	poly_C	0.989	H	H	H	M	0	0	general GO
protein_amino_acid_phosphorylation (373)	poly_C	0.989	0	0	0	0		H	general GO
organ_morphogenesis (71)	poly_C	0.989	H	H	H	0	M	0	general GO
central_nervous_system_development (68)	poly_C	0.989	0	0	0			0	general GO
cation_transport (99)	poly_C	0.989	0	H		0	0	0	general GO
Wnt_receptor_signaling_pathway (66)	SP1.01	0.778846154	H	H	0				general GO
transcription_RNA_polyII_promoter (190)	SP1.01	0.778846154	H	H	H				general GO
small_GTPase_mediated_signal_transduction	SP1.01	0.778846154				H	H	H	general GO
regulation_of_transcription_from_RNA_polymera	SP1.01	0.778846154	H	H	0	M	0	0	general GO
protein_amino_acid_phosphorylation (373)	SP1.01	0.778846154	0	0	0	0			general GO
Wnt_receptor_signaling_pathway (66)	WT1.01	0.770561018	H	H		H			general GO
transcription_RNA_polyII_promoter (190)	WT1.01	0.770561018		H	H	H	M	M	general GO
small_GTPase_mediated_signal_transduction	WT1.01	0.770561018			H	0	0	0	general GO
regulation_of_transcription_from_RNA_polymera	WT1.01	0.770561018			0	M	H	H	general GO
Wnt_receptor_signaling_pathway (66)	HES1.02	0.763263941	H	0	H	M	M	H	general GO
ubiquitin_cycle (154)	HES1.02	0.763263941	H	M	0	0	0	0	general GO
Wnt_receptor_signaling_pathway (66)	MAZR.01	0.762075134	M	0	0	H	0	H	general GO
transcription_RNA_polyII_promoter (190)	MAZR.01	0.762075134	M	0	0	H	H	0	general GO
small_GTPase_mediated_signal_transduction	MAZR.01	0.762075134			H	H	H	H	general GO
regulation_of_transcription_from_RNA_polymera	MAZR.01	0.762075134	H	H	H	0		H	general GO
Wnt_receptor_signaling_pathway (66)	ZNF202.01	0.732919255		H	H		0	H	general GO
transcription_RNA_polyII_promoter (190)	ZNF202.01	0.732919255		H	0	H			general GO
regulation_of_transcription_from_RNA_polymera	ZNF202.01	0.732919255	H	M		M	0	0	general GO
potassium_ion_transport (108)	ZNF202.01	0.732919255	H	H	H		H	0	general GO
cation_transport (99)	ZNF202.01	0.732919255	0		M	0	0	0	general GO
Wnt_receptor_signaling_pathway (66)	AP2.01	0.725490196	H	H	H				general GO
transcription_RNA_polyII_promoter (190)	AP2.01	0.725490196	0	0	H		M		general GO
small_GTPase_mediated_signal_transduction	AP2.01	0.725490196				H	0	0	general GO
morphogenesis (104)	AP2.01	0.725490196	0	0	0	H	H		general GO
Wnt_receptor_signaling_pathway (66)	ZF5.01	0.719485294	M	0	H	H	H	H	general GO
transcription_RNA_polyII_promoter (190)	ZF5.01	0.719485294	0	H		0	H	H	general GO
small_GTPase_mediated_signal_transduction	ZF5.01	0.719485294			H	0	0	H	general GO
regulation_of_progression_through_cell_cycle	ZF5.01	0.719485294	0	0	M	M	H	0	general GO
protein_transport (254)	ZF5.01	0.719485294		H	H	0	0	0	general GO
protein_biosynthesis (197)	ZF5.01	0.719485294	0	0	0		M	0	general GO
ubiquitin_cycle (154)	ZF9.01	0.717844061	H		H	0	0	0	general GO
small_GTPase_mediated_signal_transduction	ZF9.01	0.717844061				H	H	H	general GO
protein_amino_acid_phosphorylation (373)	ZF9.01	0.717844061	H	H	H				general GO
organ_morphogenesis (71)	ZF9.01	0.717844061	0	H	0	M	0	0	general GO
Wnt_receptor_signaling_pathway (66)	EGR3.01	0.709376693	0	0	M	M	H	H	general GO
small_GTPase_mediated_signal_transduction	EGR3.01	0.709376693	H	H	0	0	M	M	general GO
Wnt_receptor_signaling_pathway (66)	NRF1_01	0.708333333	H	H	0		H	H	general GO
ubiquitin_cycle (154)	NRF1_01	0.708333333		H	H	H	H	0	general GO
transcription_RNA_polyII_promoter (190)	NRF1_01	0.708333333	0	0	M	M	M		general GO
small_GTPase_mediated_signal_transduction	NRF1_01	0.708333333			H	H	H	H	general GO
regulation_of_progression_through_cell_cycle	NRF1_01	0.708333333	0	H	0	M	0	0	general GO
regulation_of_cyclin_dependent_protein_kinase_	NRF1_01	0.708333333	H	H	0	M	M	0	general GO
protein_transport (254)	NRF1_01	0.708333333		H	H	H	0	0	general GO
cell_cycle (273)	NRF1_01	0.708333333			H	0	0	0	general GO
DNA_repair (148)	MYCMAX.03	0.701731602		H	0	0	0	0	general GO
cation_transport (99)	NFKAPPAB5	0.7	0	0	0	H	0	M	general GO
apoptosis (258)	NFKAPPAB5	0.7	H		H	0	H	0	general GO
morphogenesis (104)	HAND2_E12.	0.6957522	M	0	0	0	H	H	general GO
lipid_biosynthesis (54)	HAND2_E12.	0.6957522	0	0	0	0	H	M	general GO
transcription_RNA_polyII_promoter (190)	ZBP89.01	0.695601852	0	0	0	H	M	0	general GO
transcription_RNA_polyII_promoter (190)	HELT.01	0.691910867	0	0	0	0	H	M	general GO
DNA_repair (148)	HELT.01	0.691910867	H	H	H	0		0	general GO

Wnt_receptor_signaling_pathway (66)	NGFIC.01	0.685824218	H	H				H	general GO
small_GTPase_mediated_signal_transduction	NGFIC.01	0.685824218		H	H	0	0	M	general GO
organ_morphogenesis (71)	NGFIC.01	0.685824218	0	0	0	M	M	H	general GO
morphogenesis (104)	NGFIC.01	0.685824218	0	0	H	M	0	0	general GO
calcium_ion_transport (62)	NGFIC.01	0.685824218	H	H	0	M	M	0	general GO
Wnt_receptor_signaling_pathway (66)	CKROX_01	0.683333333	H	H	H		H	0	general GO
small_GTPase_mediated_signal_transduction	CKROX_01	0.683333333			H	0	0	H	general GO
regulation_of_transcription_from_RNA_polymera	CKROX_01	0.683333333	H		H		H	0	general GO
nervous_system_development (225)	CKROX_01	0.683333333	H		H	H	H	H	general GO
chromatin_modification (53)	CKROX_01	0.683333333			H	H	0	0	general GO
Wnt_receptor_signaling_pathway (66)	EGR1.02	0.683035714	H	H		H			general GO
transcription_RNA_polyII_promoter (190)	EGR1.02	0.683035714		H	H	M	M	M	general GO
small_GTPase_mediated_signal_transduction	EGR1.02	0.683035714				H	H	H	general GO
regulation_of_transcription_from_RNA_polymera	EGR1.02	0.683035714	H		M	M	0	0	general GO
morphogenesis (104)	EGR1.02	0.683035714	H	0	H	M	M		general GO
Wnt_receptor_signaling_pathway (66)	HIC1_01	0.681615155		H	H	H	H	0	general GO
small_GTPase_mediated_signal_transduction	HIC1_01	0.681615155	H	H	H	H	0		general GO
potassium_ion_transport (108)	HIC1_01	0.681615155	H	H	H		0		general GO
organ_morphogenesis (71)	HIC1_01	0.681615155	H		0	0	H	H	general GO
Wnt_receptor_signaling_pathway (66)	EGR2.01	0.680134551	0	0	0	M	M	H	general GO
transcription_RNA_polyII_promoter (190)	GC.01	0.679856115	H	0	M	H		M	general GO
small_GTPase_mediated_signal_transduction	GC.01	0.679856115		H	H	H	0	0	general GO
ion_transport (329)	MZF1.01	0.678571429	0	0	0	M	H	0	general GO
Wnt_receptor_signaling_pathway (66)	EGR1.01	0.67232125	H			M	M		general GO
potassium_ion_transport (108)	EGR1.01	0.67232125	H		M	M	M	0	general GO
transcription_RNA_polyII_promoter (190)	MAZ.01	0.651515152	H	H	H			M	general GO
skeletal_development (83)	MAZ.01	0.651515152	H	0	M	M	0	0	general GO
regulation_of_transcription_from_RNA_polymera	MAZ.01	0.651515152					0	0	general GO
potassium_ion_transport (108)	ZIC2_01	0.648148148	0	H	0	M	0	0	general GO
epidermis_development (59)	HEN1.01	0.644124164	0	0	M	M	M		general GO
cation_transport (99)	HEN1.01	0.644124164	H	M	0	0	0	0	general GO
transcription_RNA_polyII_promoter (190)	PLAG1_01	0.63261693	M	0	H	H	H	H	general GO
regulation_of_transcription_from_RNA_polymera	PLAG1_01	0.63261693	0	M	H	M	H	H	general GO
organ_morphogenesis (71)	PLAG1_01	0.63261693	H	H		0	0	0	general GO
nervous_system_development (225)	PLAG1_01	0.63261693	H						general GO
potassium_ion_transport (108)	INSM1_01	0.629180602	0	M	M	M	M	H	general GO
organ_morphogenesis (71)	INSM1_01	0.629180602	0	H	0	H	H	M	general GO
ion_transport (329)	INSM1_01	0.629180602	0	M			H	H	general GO
Wnt_receptor_signaling_pathway (66)	AHRARNT.02	0.624493927	H	0	0	M	0	0	general GO
regulation_of_progression_through_cell_cycle	AHRARNT.02	0.624493927	0	0	H	H		H	general GO
protein_transport (254)	AHRARNT.02	0.624493927	M	0	H	H	0	0	general GO
cell_cycle (273)	AHRARNT.01	0.623253109			M	M	M	0	general GO
ubiquitin_cycle (154)	HES1.01	0.622222222		0	0	0	0	0	general GO
protein_biosynthesis (197)	HES1.01	0.622222222	0	0		M	0	0	general GO
Wnt_receptor_signaling_pathway (66)	MUSCLE_INI	0.620813397	H	0	0		H	0	general GO
small_GTPase_mediated_signal_transduction	MUSCLE_INI	0.620813397		H	H	0	0	0	general GO
central_nervous_system_development (68)	MUSCLE_INI	0.620813397	0	M	M			M	general GO
protein_transport (254)	GABP.01	0.619791667		H	0	0	0	0	general GO
protein_biosynthesis (197)	GABP.01	0.619791667				0	0	0	general GO
ER_to_Golgi_transport (47)	GABP.01	0.619791667		M	H	0	0	0	general GO
cell_division (103)	GABP.01	0.619791667	H	M	M	0	0	0	general GO
synaptic_transmission (153)	NRSF.01	0.619047619					H	0	general GO
ion_transport (329)	NRSF.01	0.619047619		M	M	0	0	0	general GO
G_protein_coupled_receptor_protein_signaling_	NRSF.01	0.619047619	H		H	H		M	general GO
transcription_RNA_polyII_promoter (190)	MUSCLE_INI	0.607655502	0	H		H	H	H	general GO
microtubule_based_movement (38)	LMO2COM.01	0.606926407	0	0	0	M	0	H	general GO
signal_transduction (1189)	NFKAPPAB.0	0.604545455				H	0	0	general GO
inflammatory_response (163)	NFKAPPAB.0	0.604545455					0	0	general GO
immune_response (366)	NFKAPPAB.0	0.604545455					0	0	general GO
chemotaxis (98)	NFKAPPAB.0	0.604545455				0	0	0	general GO
cell_cell_signaling (268)	NFKAPPAB.0	0.604545455	M	M		M	0	0	general GO
apoptosis (258)	NFKAPPAB.0	0.604545455			H	0	0	0	general GO
protein_folding (149)	ZNF76_143_(	0.6	M	0	0	0	0	H	general GO
morphogenesis (104)	ZNF76_143_(	0.6	0	0	0	0		H	general GO

endocytosis (65)	USF.03	0.6	M	0	0	0	H	0	general GO
intracellular_protein_transport (191)	ATF6.02	0.596464646	M		H	0	0	0	general GO
potassium_ion_transport (108)	RREB1.01	0.595238095	0	0	H	M	0	H	general GO
ion_transport (329)	RREB1.01	0.595238095		H	H	H	M	H	general GO
Wnt_receptor_signaling_pathway (66)	CDE.01	0.595238095	H	0	0			H	general GO
ubiquitin_cycle (154)	CDE.01	0.595238095	H		H	H	0	0	general GO
transcription_RNA_polyII_promoter (190)	CDE.01	0.595238095	0	0	H		M	M	general GO
small_GTPase_mediated_signal_transduction	CDE.01	0.595238095		H	H	H		M	general GO
protein_transport (254)	CDE.01	0.595238095				H	H	0	general GO
protein_biosynthesis (197)	CDE.01	0.595238095		H	H	H	0	H	general GO
nucleosome_assembly (71)	CDE.01	0.595238095	H	M	0	0	0	0	general GO
nuclear_mRNA_splicing_via_spliceosome (94)	CDE.01	0.595238095	H		H	H	0	H	general GO
mRNA_processing (148)	CDE.01	0.595238095			H	H	H	H	general GO
cell_cycle (273)	CDE.01	0.595238095			H	H	0	0	general GO
cell_differentiation (192)	NEUROD1.01	0.594871795	0	0	0	0	0		general GO
response_to_DNA_damage_stimulus (117)	E2F.02	0.59375			M	M	0	0	general GO
regulation_of_progression_through_cell_cycle	E2F.02	0.59375	0	H	0	0	0	M	general GO
DNA_replication (91)	E2F.02	0.59375	H	H		0	0	0	general GO
DNA_repair (148)	E2F.02	0.59375			M	M	0	0	general GO
cell_cycle (273)	E2F.02	0.59375			H	H	0	0	general GO
ubiquitin_cycle (154)	WHN.01	0.592307692	H		H	H	0	0	general GO
protein_transport (254)	WHN.01	0.592307692			H	0	0	0	general GO
protein_biosynthesis (197)	WHN.01	0.592307692	H				H	H	general GO
cell_division (103)	WHN.01	0.592307692		M			H	0	general GO
cell_cycle (273)	WHN.01	0.592307692		0	H	H	H	0	general GO
cholesterol_metabolism (38)	AREB6.03	0.588541667	0	0	H	H	0	M	general GO
intracellular_protein_transport (191)	CHREBP_ML	0.588235294	M	0	0	0	0	H	general GO
DNA_repair (148)	USF.02	0.586666667	H		H	0	0	0	general GO
protein_transport (254)	TAXCREB.01	0.584126984	H	0		M	0	0	general GO
DNA_repair (148)	MYCMAX.01	0.582417582		H	H	H	0	0	general GO
muscle_development (107)	AP4.02	0.577777778	0	M	M		0	H	general GO
Wnt_receptor_signaling_pathway (66)	PAX9.01	0.574305556			H	H	H	0	general GO
transcription_RNA_polyII_promoter (190)	PAX9.01	0.574305556	M	M	H	H	0	H	general GO
RNA_splicing (61)	NRF2.01	0.572727273	H	H		0	0	0	general GO
protein_transport (254)	NRF2.01	0.572727273		H	0	0	0	0	general GO
protein_biosynthesis (197)	NRF2.01	0.572727273				M	0	0	general GO
nuclear_mRNA_splicing_via_spliceosome (94)	NRF2.01	0.572727273		H	H	0	0	0	general GO
synaptic_transmission (153)	NRSE.01	0.571957672				M	H	0	general GO
ion_transport (329)	NRSE.01	0.571957672		M	M	0	H	0	general GO
G_protein_coupled_receptor_protein_signaling_	NRSE.01	0.571957672	H	0	H	M		M	general GO
immune_response (366)	NF1.02	0.570065789	H	H	0	0	M	0	general GO
electron_transport (214)	NF1.02	0.570065789	M	M	0	H	0	0	general GO
signal_transduction (1189)	NFKAPPAB.0	0.56884058				0	H	H	general GO
sensory_perception (201)	NFKAPPAB.0	0.56884058	H		M	0	0	0	general GO
inflammatory_response (163)	NFKAPPAB.0	0.56884058				M	0	0	general GO
immune_response (366)	NFKAPPAB.0	0.56884058					H	0	general GO
chemotaxis (98)	NFKAPPAB.0	0.56884058				M	0	0	general GO
Wnt_receptor_signaling_pathway (66)	PAX5.01	0.568181818	0	H			M	0	general GO
small_GTPase_mediated_signal_transduction	PAX5.01	0.568181818	M	0		0	0	0	general GO
potassium_ion_transport (108)	PAX5.01	0.568181818	0	0	H		H		general GO
transmembrane_receptor_tyrosine_kinase_signa	GAGA.01	0.562878788	M	0	0	H	0	0	general GO
synaptic_transmission (153)	GAGA.01	0.562878788	H	H	0	0		0	general GO
signal_transduction (1189)	GAGA.01	0.562878788	H		0	H	H	H	general GO
potassium_ion_transport (108)	GAGA.01	0.562878788	H	H	H		0	H	general GO
nervous_system_development (225)	GAGA.01	0.562878788		H	0	0	0	0	general GO
ion_transport (329)	GAGA.01	0.562878788	H	H	H				general GO
calcium_ion_transport (62)	GAGA.01	0.562878788	H	H	H			H	general GO
ion_transport (329)	VDR_RXR.01	0.561111111	H	H	H		H	H	general GO
protein_transport (254)	CREB.02	0.56	M		H	H	H	H	general GO
protein_biosynthesis (197)	CREB.02	0.56	0		H	0	0	0	general GO
lipid_transport (41)	FTF.01	0.557291667	M	0	0	H	H	0	general GO
inflammatory_response (163)	NFKAPPAB65	0.554545455					0	0	general GO
immune_response (366)	NFKAPPAB65	0.554545455					H	0	general GO
chemotaxis (98)	NFKAPPAB65	0.554545455				0	0	0	general GO

cell_cell_signaling (268)	NFKAPPAB6	0.554545455				M	0	0	general GO
apoptosis (258)	NFKAPPAB6	0.554545455				H	H	0	general GO
anti_apoptosis (85)	NFKAPPAB6	0.554545455		M		H	0	0	general GO
protein_biosynthesis (197)	ATF.01	0.554187192		M	M	M	0	0	general GO
ER_to_Golgi_transport (47)	ATF.01	0.554187192			0	0	0	0	general GO
protein_biosynthesis (197)	CETS1P54.0	0.552631579				M	0	0	general GO
regulation_of_progression_through_cell_cycle	CREB.04	0.552083333	H	H	H	M	0	0	general GO
protein_biosynthesis (197)	CREB.04	0.552083333	H			H	H	H	general GO
protein_biosynthesis (197)	YY1.01	0.550877193			0	0	0	0	general GO
nuclear_mRNA_splicing_via_spliceosome (94)	YY1.01	0.550877193	M	H	0	0	H	H	general GO
mRNA_processing (148)	YY1.01	0.550877193	M		0	0	H	0	general GO
inflammatory_response (163)	CREL.01	0.547619048				M	0	M	general GO
immune_response (366)	CREL.01	0.547619048					0	0	general GO
chemotaxis (98)	CREL.01	0.547619048			M	M	0	0	general GO
apoptosis (258)	CREL.01	0.547619048				M	0	0	general GO
protein_biosynthesis (197)	ELK1.02	0.547619048				M	M	0	general GO
DNA_replication (91)	ELK1.02	0.547619048	H	0		M	0	0	general GO
DNA_repair (148)	ELK1.02	0.547619048			M	0	0	0	general GO
cell_cycle (273)	ELK1.02	0.547619048	0	0	0	0		H	general GO
signal_transduction (1189)	NFKAPPAB.0	0.546218487		M	M	0	M	M	general GO
inflammatory_response (163)	NFKAPPAB.0	0.546218487					0	0	general GO
immune_response (366)	NFKAPPAB.0	0.546218487				H	H	0	general GO
chemotaxis (98)	NFKAPPAB.0	0.546218487				H	0	0	general GO
cell_cell_signaling (268)	NFKAPPAB.0	0.546218487				M	0	0	general GO
protein_biosynthesis (197)	ZNF35_01	0.545808967	H	M	0	0	0	0	general GO
regulation_of_progression_through_cell_cycle	CREB.03	0.543859649	H	0	0	M	0	0	general GO
protein_folding (149)	CREB.03	0.543859649	H		H	0	0	0	general GO
protein_biosynthesis (197)	CREB.03	0.543859649	H			0	0	0	general GO
protein_folding (149)	MYCMAX.02	0.542424242	H	H	H			0	general GO
regulation_of_cyclin_dependent_protein_kinase_	ACAAT.01	0.541666667	0	0	H		0	0	general GO
small_GTPase_mediated_signal_transduction	NUDR.01	0.539697282	0		0	0	0	0	general GO
protein_biosynthesis (197)	NUDR.01	0.539697282	0		M	M	0	0	general GO
response_to_DNA_damage_stimulus (117)	E2F.03	0.538461538			M	M	0	0	general GO
regulation_of_progression_through_cell_cycle	E2F.03	0.538461538	0	0	0		M	M	general GO
protein_biosynthesis (197)	E2F.03	0.538461538					H	H	general GO
DNA_repair (148)	E2F.03	0.538461538			0	M	0	0	general GO
cell_cycle (273)	E2F.03	0.538461538	H			H	H	0	general GO
protein_biosynthesis (197)	ELF2.01	0.536217949			M	0	0	0	general GO
ubiquitin_cycle (154)	FLI.01	0.534588274				0	0	0	general GO
response_to_DNA_damage_stimulus (117)	FLI.01	0.534588274				0	0	0	general GO
protein_biosynthesis (197)	FLI.01	0.534588274				M	0	0	general GO
DNA_repair (148)	FLI.01	0.534588274		H	H	H	0	0	general GO
Wnt_receptor_signaling_pathway (66)	HNF4.02	0.533333333	0	0	M		0	0	general GO
positive_regulation_of_cell_proliferation (103)	NF1.01	0.532058119		H	0	0	0	0	general GO
electron_transport (214)	NF1.01	0.532058119		H	H	H	0	0	general GO
chemotaxis (98)	NF1.01	0.532058119		H	0	0	0	0	general GO
electron_transport (214)	HNF4.01	0.531055901				0	0	0	general GO
chemotaxis (98)	BACH2.01	0.525874126	0	M	H	0	0	0	general GO
response_to_unfolded_protein (40)	STAT1.01	0.524475687	0	0	0	M	H	0	general GO
protein_transport (254)	STAT1.01	0.524475687	H	H	H	M	0	0	general GO
signal_transduction (1189)	HIVEP1_01	0.524475524		M	M	M	0	0	general GO
inflammatory_response (163)	HIVEP1_01	0.524475524					0	0	general GO
immune_response (366)	HIVEP1_01	0.524475524				H	H	0	general GO
chemotaxis (98)	HIVEP1_01	0.524475524		H	H	0	0	0	general GO
cell_cell_signaling (268)	HIVEP1_01	0.524475524				M	M	0	general GO
signal_transduction (1189)	AML1.01	0.520833333	0		0	M	0	0	general GO
pregnancy (41)	AML1.01	0.520833333	M	M	0	0	H	H	general GO
immune_response (366)	AML1.01	0.520833333			H	H	0	0	general GO
chemotaxis (98)	AML1.01	0.520833333	H		0	0	0	0	general GO
proton_transport (41)	T3R.01	0.520634921	0	0	H	0	H		general GO
microtubule_based_movement (38)	T3R.01	0.520634921	M	0	M	M	H	0	general GO
blood_coagulation (70)	T3R.01	0.520634921	0	H	H	H	0	M	general GO
transport (940)	ERR_01	0.519924812	M	H	H	H	H	H	general GO
generation_of_precursor_metabolites_and_ener	ERR_01	0.519924812	M		M	0	0	0	general GO

amino_acid_metabolism (45)	ERR_01	0.519924812	H	H	M	0	0	0	general GO
immune_response (366)	PARAXIS_01	0.51879085	H	0	M	M	M	M	general GO
defense_response (100)	PARAXIS_01	0.51879085	0	H	H	H			general GO
cell_cell_signaling (268)	PARAXIS_01	0.51879085	0		M	M	0	0	general GO
visual_perception (142)	VMAF.01	0.518467852		H	0	0	0	0	general GO
steroid_biosynthesis (42)	NFY.01	0.518088822		H	H	H	0	0	general GO
regulation_of_cyclin_dependent_protein_kinase_	NFY.01	0.518088822	H	H	H		M	0	general GO
mRNA_processing (148)	NFY.01	0.518088822	0	0	0	H	M	M	general GO
mitosis (86)	NFY.01	0.518088822				H	0	0	general GO
DNA_replication (91)	NFY.01	0.518088822		H	H	0	0	0	general GO
cell_division (103)	NFY.01	0.518088822					0	0	general GO
cell_cycle (273)	NFY.01	0.518088822					H	0	general GO
epidermis_development (59)	E47.02	0.517307692		0	0	H	H	0	general GO
cell_proliferation (243)	E47.02	0.517307692	0	0	M	M		M	general GO
immune_response (366)	ETS1.01	0.516363636		H	H	H	0	0	general GO
antimicrobial_humoral_response (87)	ETS1.01	0.516363636	M		H	0	0	0	general GO
protein_biosynthesis (197)	ELK1.01	0.515625				0	0	0	general GO
sodium_ion_transport (75)	FXRE.01	0.512820513	0	M		M	M	H	general GO
apoptosis (258)	AP1FJ.01	0.507936508	0	0		H	H	0	general GO
protein_transport (254)	E4F.01	0.505787037	M	M	0	0	0	H	general GO
visual_perception (142)	NRL.01	0.505639098	H	H	M	0	0	0	general GO
cholesterol_metabolism (38)	TEF1.01	0.505208333	H	H	0	0	0	M	general GO
proteolysis (297)	TH1E47.01	0.505050505		M	M	M	0	0	general GO
immune_response (366)	AML3.01	0.503401361				H	0	0	general GO
cellular_defense_response (68)	AML3.01	0.503401361	H	H	H		H	M	general GO
inflammatory_response (163)	RBPJK.02	0.501754386		H	H	0	0	0	general GO
skeletal_development (83)	AG_rich_codi	0.5	H		H	0	0	0	general GO
lipid_metabolism (196)	NBRE.01	0.5		H	H	H	H	0	general GO
immune_response (366)	NBRE.01	0.5		M	M	0	0	0	general GO
signal_transduction (1189)	TAL1ALPHAE	0.497212739	M		M	0	0	0	general GO
muscle_development (107)	TAL1ALPHAE	0.497212739	0	0	H	M	0	0	general GO
inflammatory_response (163)	TAL1ALPHAE	0.497212739	M	H	M	0	0	0	general GO
epidermis_development (59)	TAL1ALPHAE	0.497212739		0	0	0	0	0	general GO
cell_cell_signaling (268)	TAL1ALPHAE	0.497212739	0	H	M	0	0	0	general GO
immune_response (366)	NFE2.01	0.496503497		H	H	H	H	H	general GO
chemotaxis (98)	NFE2.01	0.496503497			H	0	0	0	general GO
response_to_DNA_damage_stimulus (117)	E2F.01	0.496296296					M	M	general GO
DNA_repair (148)	E2F.01	0.496296296					M	0	general GO
cell_cycle (273)	E2F.01	0.496296296	H	H	H	0	0	M	general GO
protein_folding (149)	SRF.02	0.494285714	0	0	H	H	0	M	general GO
muscle_development (107)	SRF.02	0.494285714				H	0	0	general GO
immune_response (366)	AP1.02	0.49382716		H		H	H	H	general GO
chemotaxis (98)	AP1.02	0.49382716			M	0	0	0	general GO
protein_transport (254)	ATF.02	0.493506494	M	M	H	0	0	0	general GO
intracellular_protein_transport (191)	ATF.02	0.493506494	M	M	0	H	H	0	general GO
cell_cycle (273)	ATF.02	0.493506494	0	0		M	0	0	general GO
epidermis_development (59)	TBX5.01	0.492603182		H	H	M	0	0	general GO
sensory_perception (201)	TR2.01	0.490909091			H	H	H	H	general GO
excretion (35)	TR2.01	0.490909091	H		M		M	0	general GO
steroid_biosynthesis (42)	CAAT.01	0.489080752				H	0	0	general GO
regulation_of_cyclin_dependent_protein_kinase_	CAAT.01	0.489080752	H	H			0	0	general GO
mitosis (86)	CAAT.01	0.489080752				0	0	0	general GO
chromosome_organization_and_biogenesis (83)	CAAT.01	0.489080752		H	H	H	0	0	general GO
cell_division (103)	CAAT.01	0.489080752					0	0	general GO
cell_cycle (273)	CAAT.01	0.489080752				M	0	0	general GO
biosynthesis (32)	CAAT.01	0.489080752		H	H	H	0	0	general GO
signal_transduction (1189)	TAL1BETAE4	0.488324176	0	H	H	M	M	0	general GO
sensory_perception (201)	TAL1BETAE4	0.488324176	H		H	H	0	H	general GO
inflammatory_response (163)	TAL1BETAE4	0.488324176	M	M	H	0	0	0	general GO
immune_response (366)	TAL1BETAE4	0.488324176					0	0	general GO
G_protein_coupled_receptor_protein_signaling_	TAL1BETAE4	0.488324176	M		M	0	0	0	general GO
cell_cell_signaling (268)	TAL1BETAE4	0.488324176			M	0	0	0	general GO
ubiquitin_cycle (154)	CMYB.02	0.488290398	0	0	M	M	H	H	general GO
cell_cycle (273)	CMYB.02	0.488290398	H			0	0	H	general GO

metabolism (241)	IK3.01	0.48806366	0	0	M	0		H	general GO
elevation_of_cytosolic_calcium_ion_concentratio	AREB6.01	0.485576923	0	M	H	0	0	0	general GO
transport (940)	TR4.01	0.484188034	H	H	M		H	H	general GO
lipid_metabolism (196)	TR4.01	0.484188034	H	H	0		0	0	general GO
transport (940)	COUP.01	0.483193277	0	M	0	H	0	H	general GO
metabolism (241)	COUP.01	0.483193277	M	H	0	0	H	0	general GO
fatty_acid_metabolism (55)	COUP.01	0.483193277	H		M	0	0	0	general GO
sensory_perception (201)	LEF1.01	0.482102273	H	H	M	0	0	0	general GO
immune_response (366)	LEF1.01	0.482102273				H	H	0	general GO
protein_kinase_cascade (43)	PU1.01	0.480025183	0	M	M	M	M		general GO
inflammatory_response (163)	PU1.01	0.480025183	0	0	M		0	0	general GO
immune_response (366)	PU1.01	0.480025183	H	H		0	0	0	general GO
inflammatory_response (163)	ETS2.01	0.478021978				0	0	0	general GO
immune_response (366)	ETS2.01	0.478021978				0	0	0	general GO
chemotaxis (98)	ETS2.01	0.478021978	M		H	0	0	0	general GO
cellular_defense_response (68)	ETS2.01	0.478021978		M	M	0	0	0	general GO
cell_surface_receptor_linked_signal_transductio	ETS2.01	0.478021978				0	0	0	general GO
steroid_biosynthesis (42)	NFY.02	0.477272727				H	0	0	general GO
mitosis (86)	NFY.02	0.477272727				M	0	0	general GO
chromosome_organization_and_biogenesis (83)	NFY.02	0.477272727		H	H	H	0	0	general GO
cell_division (103)	NFY.02	0.477272727					0	0	general GO
cell_cycle (273)	NFY.02	0.477272727				H	0	0	general GO
biosynthesis (32)	NFY.02	0.477272727				H	0	0	general GO
intracellular_protein_transport (191)	CREB.01	0.477272727	M	M	0	0	0	H	general GO
protein_transport (254)	XBP1.01	0.473873874	M	M	0	0	0	H	general GO
intracellular_protein_transport (191)	XBP1.01	0.473873874	M	M	0	0	H	H	general GO
innate_immune_response (46)	GRE.01	0.473684211			0	0	0	0	general GO
immune_response (366)	GRE.01	0.473684211		H	H	0	0	0	general GO
cell_surface_receptor_linked_signal_transductio	GRE.01	0.473684211	0	M	0	H	H	0	general GO
immune_response (366)	PAX2.01	0.473429952		H	H	H	0	0	general GO
sensory_perception (201)	AP1.03	0.473251029		H	0	0	0	0	general GO
immune_response (366)	AP1.03	0.473251029		H	H	H	H	H	general GO
chemotaxis (98)	AP1.03	0.473251029	M		M	0	0	0	general GO
cellular_defense_response (68)	AP1.03	0.473251029	0	0	H	0	H	M	general GO
electron_transport (214)	PPARA.01	0.472727273		M	M	0	0	0	general GO
chemotaxis (98)	PPARA.01	0.472727273	0	0	0	0	M	H	general GO
immune_response (366)	NFE2L2.01	0.470833333		H	H	H	H	0	general GO
electron_transport (214)	NFE2L2.01	0.470833333			0	0	0	0	general GO
chemotaxis (98)	NFE2L2.01	0.470833333			H	0	0	H	general GO
signal_transduction (1189)	TAL1BETAIT	0.46978022	M	H		M	M	0	general GO
immune_response (366)	TAL1BETAIT	0.46978022					0	0	general GO
G_protein_coupled_receptor_protein_signaling_	TAL1BETAIT	0.46978022				M	0	0	general GO
cell_cell_signaling (268)	TAL1BETAIT	0.46978022		H	M	0	0	0	general GO
cell_adhesion (378)	TAL1BETAIT	0.46978022	H	0	0		H	H	general GO
mRNA_processing (148)	NFY.03	0.46875	H	H	H	0	M	M	general GO
mitosis (86)	NFY.03	0.46875				M	0	0	general GO
chromosome_organization_and_biogenesis (83)	NFY.03	0.46875		H	H	H	0	0	general GO
cell_division (103)	NFY.03	0.46875				M	0	0	general GO
cell_cycle (273)	NFY.03	0.46875				M	0	0	general GO
sensory_perception (201)	SRF.03	0.468253968			H	H	0	0	general GO
muscle_development (107)	SRF.03	0.468253968				H	H	0	general GO
inflammatory_response (163)	SRF.03	0.468253968			H	0	0	0	general GO
immune_response (366)	SRF.03	0.468253968	H					0	general GO
immune_response (366)	BACH1.01	0.467741935				H	H	H	general GO
epidermis_development (59)	BACH1.01	0.467741935	H	0	M	M	M	M	general GO
electron_transport (214)	PLZF.01	0.467532468			H	0	0	0	general GO
immune_response (366)	AREB6.04	0.465277778		H	0	0	0	0	general GO
G_protein_coupled_receptor_protein_signaling_	MEL1_02	0.464795009		H	H	0	0	0	general GO
defense_response (100)	TAACC.01	0.462375675	0	H	M	0	0	0	general GO
immune_response (366)	STAT6.01	0.461111111	H	H		H	0	0	general GO
signal_transduction (1189)	MIT.01	0.46076555		M	0	0	0	0	general GO
inflammatory_response (163)	MIT.01	0.46076555			H	H	H	H	general GO
immune_response (366)	MIT.01	0.46076555					M	H	general GO
sensory_perception (201)	MEL1_03	0.46	H	H	H	0	0	M	general GO

immune_response (366)	MEL1_03	0.46		H	H	H	H	H	general GO
cell_cell_signaling (268)	MEL1_03	0.46		0	H	0	0	0	general GO
sensory_perception (201)	RP58.01	0.457532051	H	H	M	0	0	0	general GO
immune_response (366)	RP58.01	0.457532051	H	H			M	M	general GO
inflammatory_response (163)	CEBP.02	0.457142857		H	H	H	0	0	general GO
sensory_perception (201)	COMP1.01	0.454545455		H		H	M		general GO
inflammatory_response (163)	COMP1.01	0.454545455				H	0	0	general GO
immune_response (366)	COMP1.01	0.454545455	H	H	0	0		H	general GO
chemotaxis (98)	COMP1.01	0.454545455	H	0	0	0	0	M	general GO
immune_response (366)	PXRCAR.01	0.452173913				M	H	0	general GO
inflammatory_response (163)	DBP.01	0.450909091		H	H	H	0	0	general GO
cell_proliferation (243)	DBP.01	0.450909091	0		0	0	0	0	general GO
inflammatory_response (163)	BARBIE.01	0.449122807	H	H		0	0	0	general GO
immune_response (366)	BARBIE.01	0.449122807				H	0	0	general GO
immune_response (366)	BRACH.01	0.448863636	M	H	0	0	0	0	general GO
G_protein_coupled_receptor_protein_signaling_	BRACH.01	0.448863636		H	H	H	0	0	general GO
spermatogenesis (97)	VMYB.03	0.446686196	M	H	0	0	0	0	general GO
inflammatory_response (163)	PAX8.01	0.446153846		H	H	0	0	0	general GO
immune_response (366)	PAX8.01	0.446153846		M	M	0	0	0	general GO
transport (940)	GATA1.01	0.446146245	0	M	M	M		H	general GO
proteolysis (297)	GATA1.01	0.446146245	H	H	H	0	0	M	general GO
inflammatory_response (163)	GATA1.01	0.446146245	M	M		0	M	0	general GO
cell_surface_receptor_linked_signal_transductio	GATA1.01	0.446146245		0	0	0	0	0	general GO
inflammatory_response (163)	MYT1L.01	0.444444444			0	H	0	H	general GO
immune_response (366)	MYT1L.01	0.444444444		H	H	H	0	0	general GO
immune_response (366)	STAT.01	0.444444444				H	0	0	general GO
ubiquitin_cycle (154)	VMYB.05	0.444012751	H	H		H	H	H	general GO
innate_immune_response (46)	PRE.01	0.442831216			H	H	0	0	general GO
inflammatory_response (163)	PRE.01	0.442831216			H	H	H	H	general GO
immune_response (366)	PRE.01	0.442831216			H	0	0	0	general GO
immune_response (366)	TCF11.01	0.440816327					M	0	general GO
G_protein_coupled_receptor_protein_signaling_	TCF11.01	0.440816327	H			0	0	0	general GO
sensory_perception (201)	BCL6.02	0.436298077	H	0	M	M	M	0	general GO
inflammatory_response (163)	BCL6.02	0.436298077					M	0	general GO
immune_response (366)	BCL6.02	0.436298077					M	0	general GO
chemotaxis (98)	BCL6.02	0.436298077		M	M	M	M	0	general GO
cell_cell_signaling (268)	BCL6.02	0.436298077	0	0	0		0	0	general GO
calcium_ion_homeostasis (30)	BCL6.02	0.436298077	H	H			H	H	general GO
proteolysis (297)	TCF11MAFG	0.434095861			M	0	0	0	general GO
inflammatory_response (163)	TCF11MAFG	0.434095861			H	H	0	0	general GO
immune_response (366)	TCF11MAFG	0.434095861				H	H	0	general GO
chemotaxis (98)	TCF11MAFG	0.434095861		H	H	H	0	0	general GO
immune_response (366)	HOX1-3.01	0.433673469		H	M	0	M	H	general GO
innate_immune_response (46)	TST1.01	0.433333333			M	0	0	0	general GO
antimicrobial_humoral_response (87)	TST1.01	0.433333333		H	0	0	0	0	general GO
immune_response (366)	ILF1_01	0.433333333				H	0	0	general GO
signal_transduction (1189)	AP1.01	0.432748538		M	0	0	0	0	general GO
innate_immune_response (46)	AP1.01	0.432748538	0	0		H	0	0	general GO
inflammatory_response (163)	AP1.01	0.432748538			H	0	0	0	general GO
immune_response (366)	AP1.01	0.432748538				H	H	H	general GO
epidermis_development (59)	AP1.01	0.432748538	H	0	M	M	M	M	general GO
elevation_of_cytosolic_calcium_ion_concentratio	AP1.01	0.432748538	M	H	H	0	0	0	general GO
chemotaxis (98)	AP1.01	0.432748538			H	H	0	0	general GO
cell_surface_receptor_linked_signal_transductio	AP1.01	0.432748538	H		0	0	0	0	general GO
innate_immune_response (46)	ISL1.01	0.430586081	H	M	0	0	M	0	general GO
inflammatory_response (163)	ISL1.01	0.430586081			H	H	H	H	general GO
response_to_virus (55)	ISRE.01	0.42745098						H	general GO
immune_response (366)	ISRE.01	0.42745098					0	0	general GO
cell_cell_signaling (268)	ISRE.01	0.42745098			H	0	0	0	general GO
antimicrobial_humoral_response (87)	ISRE.01	0.42745098		H	H	H	H	0	general GO
ubiquitin_dependent_protein_catabolism (72)	IRF2.01	0.427185044			H	H	0	0	general GO
response_to_virus (55)	IRF2.01	0.427185044					H	H	general GO
inflammatory_response (163)	IRF2.01	0.427185044		M	M	0	0	0	general GO
immune_response (366)	IRF2.01	0.427185044				M	0	0	general GO

cell_surface_receptor_linked_signal_transductio	IRF2.01	0.427185044				H	H	0	general GO
homophilic_cell_adhesion (70)	PDX1_G_SA	0.426470588	0	0	0	M	H	H	general GO
ubiquitin_dependent_protein_catabolism (72)	IRF3.01	0.416666667			M	0	0	0	general GO
response_to_virus (55)	IRF3.01	0.416666667				0	0	0	general GO
positive_regulation_of_I_kappaB_kinase_NF_ka	IRF3.01	0.416666667		0	0	0	0	0	general GO
innate_immune_response (46)	IRF3.01	0.416666667	M	0	0	H	0	0	general GO
inflammatory_response (163)	IRF3.01	0.416666667	H	H		H	H	0	general GO
immune_response (366)	IRF3.01	0.416666667					0	0	general GO
sensory_perception (201)	SRF.01	0.416534181				H	H	0	general GO
muscle_development (107)	SRF.01	0.416534181				M	0	0	general GO
inflammatory_response (163)	SRF.01	0.416534181				0	0	0	general GO
immune_response (366)	SRF.01	0.416534181					M	H	general GO
circulation (52)	SRF.01	0.416534181	0	0	H	0	0	M	general GO
chemotaxis (98)	SRF.01	0.416534181	H			0	0	0	general GO
response_to_virus (55)	PRDM1.01	0.412696678						H	general GO
innate_immune_response (46)	PRDM1.01	0.412696678	M	0	H	H	H	H	general GO
inflammatory_response (163)	PRDM1.01	0.412696678				H	0	0	general GO
immune_response (366)	PRDM1.01	0.412696678				H	0	0	general GO
defense_response (100)	PRDM1.01	0.412696678		0	H	0	H	0	general GO
innate_immune_response (46)	HMGIY.01	0.412049489			0	0	0	0	general GO
inflammatory_response (163)	HMGIY.01	0.412049489						M	general GO
immune_response (366)	HMGIY.01	0.412049489				H	H	H	general GO
immune_response (366)	FAST1.01	0.411564626			0	0	0	0	general GO
inflammatory_response (163)	GATA.01	0.411111111			H	H	0	M	general GO
immune_response (366)	GATA.01	0.411111111		H	H	H	H	H	general GO
circulation (52)	GATA.01	0.411111111		H		0	0	0	general GO
signal_transduction (1189)	PBX1_MEIS1	0.410675303	0	0	M	M	H	0	general GO
sensory_perception (201)	HBP1_01	0.410364146				H	H	H	general GO
response_to_virus (55)	HBP1_01	0.410364146		H	H	0	0	0	general GO
inflammatory_response (163)	HBP1_01	0.410364146		H	H	H	0	M	general GO
immune_response (366)	HBP1_01	0.410364146			H	H	0	0	general GO
cell_surface_receptor_linked_signal_transductio	HBP1_01	0.410364146			M	M	0	0	general GO
sensory_perception (201)	GFI1.01	0.408015514		H	H	H	H	0	general GO
immune_response (366)	GFI1.01	0.408015514	H	0	H	H	M	M	general GO
immune_response (366)	PHOX2_01	0.407575758		H	H	0	H	H	general GO
immune_response (366)	PSE_02	0.407407407			H	H	H	H	general GO
chemotaxis (98)	PSE_02	0.407407407	H	H			0	0	general GO
mitosis (86)	CHR.01	0.405555556			M	0	0	0	general GO
cell_division (103)	CHR.01	0.405555556			M	0	0	0	general GO
inflammatory_response (163)	BRN4.01	0.404761905		0	H	H	0	0	general GO
immune_response (366)	BRN4.01	0.404761905		H	H	H	H	H	general GO
sensory_perception (201)	MTBF.01	0.404040404		H	H	0	0	0	general GO
inflammatory_response (163)	LEF1.02	0.404040404		H	M	H	H	H	general GO
inflammatory_response (163)	MTBF.01	0.404040404		H	H	H	0	H	general GO
immune_response (366)	MTBF.01	0.404040404		H	H	H	0	0	general GO
chemotaxis (98)	MTBF.01	0.404040404		H	H	H	H	H	general GO
cell_cell_signaling (268)	MTBF.01	0.404040404			H	0	0	0	general GO
response_to_virus (55)	IRF1.01	0.403617536						H	general GO
regulation_of_apoptosis (59)	IRF1.01	0.403617536	M	0	M	0	H	H	general GO
inflammatory_response (163)	IRF1.01	0.403617536				0	0	0	general GO
immune_response (366)	IRF1.01	0.403617536				M	0	0	general GO
cell_surface_receptor_linked_signal_transductio	IRF1.01	0.403617536	M	H	H	H	H	H	general GO
response_to_virus (55)	CEBPB.01	0.4		0	0	0	0	0	general GO
proteolysis (297)	CEBPB.01	0.4					0	0	general GO
innate_immune_response (46)	CEBPB.01	0.4		H	H	H	H	H	general GO
inflammatory_response (163)	CEBPB.01	0.4		H	H	H	H	H	general GO
inflammatory_response (163)	GATA1.04	0.4		H	0	0	0	0	general GO
immune_response (366)	CEBPB.01	0.4		H	H	H	H	H	general GO
immune_response (366)	GATA1.04	0.4		H	H	H	H	0	general GO
defense_response (100)	GATA1.04	0.4	H	H		H	H	0	general GO
cell_surface_receptor_linked_signal_transductio	CEBPB.01	0.4		H	H	H	0	0	general GO
blood_coagulation (70)	CEBPB.01	0.4	M			0	0	0	general GO
antimicrobial_humoral_response (87)	CEBPB.01	0.4	H	H	H	M	0	0	general GO
inflammatory_response (163)	MEIS1B_HO	0.394957983	M			H	0	H	general GO

immune_response (366)	OCT1.05	0.392857143					H	0	general GO
signal_transduction (1189)	AARE.01	0.391812865		0	0	0	0	0	general GO
sensory_perception (201)	AARE.01	0.391812865	H	H			0	0	general GO
inflammatory_response (163)	AARE.01	0.391812865		H	H	0	0	0	general GO
immune_response (366)	AARE.01	0.391812865		H	H	H	0	0	general GO
inflammatory_response (163)	AIRE.01	0.391419855		H	H	H	H	0	general GO
immune_response (366)	AIRE.01	0.391419855				H	H	H	general GO
sensory_perception (201)	LTATA_01	0.39		0	H	H	0	0	general GO
RNA_processing (58)	LTATA_01	0.39	M	M	M	0	0	H	general GO
proteolysis (297)	LTATA_01	0.39		H	0	0	0	0	general GO
nucleosome_assembly (71)	LTATA_01	0.39	H			H	H	0	general GO
inflammatory_response (163)	LTATA_01	0.39				0	0	0	general GO
cell_cell_signaling (268)	LTATA_01	0.39		0	0	0	0	0	general GO
inflammatory_response (163)	BRN2.01	0.389384921				H	0	H	general GO
antimicrobial_humoral_response (87)	BRN2.01	0.389384921	H	H		H	0	0	general GO
proteolysis (297)	IRF4.01	0.388961039		0	0	0	0	0	general GO
innate_immune_response (46)	IRF4.01	0.388961039		H	H	H	0	0	general GO
inflammatory_response (163)	IRF4.01	0.388961039		H	H	H	H	0	general GO
immune_response (366)	IRF4.01	0.388961039				M	0	0	general GO
inflammatory_response (163)	STAT5.01	0.388571429		H	0	H	H	H	general GO
immune_response (366)	STAT5.01	0.388571429					M	0	general GO
cell_surface_receptor_linked_signal_transductio	STAT5.01	0.388571429	H	H	H	M	0	0	general GO
inflammatory_response (163)	GATA1.03	0.387685485			H	H	H	H	general GO
immune_response (366)	GATA1.03	0.387685485		H	H	H	H	H	general GO
chemotaxis (98)	GATA1.03	0.387685485		H	H	H	H	H	general GO
inflammatory_response (163)	BCL6.01	0.385599694					H	M	general GO
immune_response (366)	BCL6.01	0.385599694					0	0	general GO
chemotaxis (98)	BCL6.01	0.385599694	0	0		H	0	0	general GO
inflammatory_response (163)	OCT1.04	0.383116883			H	H	0	0	general GO
immune_response (366)	OCT1.04	0.383116883				H	H	0	general GO
sensory_perception (201)	GFI1B.01	0.382387888			H	0	H	H	general GO
immune_response (366)	GFI1B.01	0.382387888		H	H	0	0	0	general GO
inflammatory_response (163)	PDX1_G_SA/	0.380252101	0	M	M	H	0	0	general GO
sensory_perception (201)	AMEF2.01	0.379545455	H		H	H		M	general GO
inflammatory_response (163)	AMEF2.01	0.379545455	H			H	0	0	general GO
immune_response (366)	AMEF2.01	0.379545455		M		M	0	0	general GO
inflammatory_response (163)	NFAT.01	0.378787879				H	H	0	general GO
immune_response (366)	NFAT.01	0.378787879				H	0	0	general GO
homophilic_cell_adhesion (70)	NFAT.01	0.378787879		H	0	H	H	0	general GO
cell_adhesion (378)	NFAT.01	0.378787879	M			H	0	0	general GO
inflammatory_response (163)	FREAC4.01	0.377604167				0	0	0	general GO
immune_response (366)	FREAC4.01	0.377604167		M	0	0	0	0	general GO
sensory_perception (201)	CDX2.01	0.375438596	H	H	H		H	H	general GO
innate_immune_response (46)	CDX2.01	0.375438596	M		H			H	general GO
inflammatory_response (163)	CDX2.01	0.375438596			H		H	H	general GO
immune_response (366)	CDX2.01	0.375438596		H	H	H	H	0	general GO
antimicrobial_humoral_response (87)	CDX2.01	0.375438596	H	H	M	0	0	H	general GO
innate_immune_response (46)	MEF2.01	0.375		H	H	H	H	0	general GO
inflammatory_response (163)	MEF2.01	0.375	H		H	H	0	0	general GO
immune_response (366)	MEF2.01	0.375		H	H	0	0	0	general GO
sensory_perception (201)	MMEF2.01	0.373626374	H	H	H				general GO
proteolysis (297)	MMEF2.01	0.373626374		H	H	0	0	0	general GO
inflammatory_response (163)	MMEF2.01	0.373626374		H	H	H	0	0	general GO
immune_response (366)	MMEF2.01	0.373626374			M	0	0	0	general GO
inflammatory_response (163)	SOX9.01	0.370192308	0	H		H	H	H	general GO
signal_transduction (1189)	GATA2.02	0.363636364		0	H	H	0	0	general GO
sensory_perception (201)	GATA2.02	0.363636364				H	H	H	general GO
inflammatory_response (163)	GATA2.02	0.363636364		H	H	0	0	M	general GO
immune_response (366)	GATA2.02	0.363636364					H		general GO
G_protein_coupled_receptor_protein_signaling_	GATA2.02	0.363636364			H	H	H	H	general GO
chemotaxis (98)	GATA2.02	0.363636364		H	H	H	H	H	general GO
response_to_virus (55)	GATA1.05	0.363157895	H	H	0	0		0	general GO
innate_immune_response (46)	GATA1.05	0.363157895	H			H	H	0	general GO
inflammatory_response (163)	GATA1.05	0.363157895	H	M	H	0	0	0	general GO

immune_response (366)	GATA1.05	0.363157895			H	H	H	H	general GO
response_to_virus (55)	IRF7.01	0.356770833			H	0	0	0	general GO
inflammatory_response (163)	IRF7.01	0.356770833		H	H	H	0	0	general GO
immune_response (366)	IRF7.01	0.356770833					0	0	general GO
inflammatory_response (163)	OCT1P.01	0.356759907			H	H	H	H	general GO
immune_response (366)	OCT1P.01	0.356759907		H			0	0	general GO
antimicrobial_humoral_response (87)	OCT1P.01	0.356759907	H	H	H	H	0	M	general GO
sensory_perception (201)	OCT.01	0.354700855	H	H		H	0	0	general GO
inflammatory_response (163)	OCT.01	0.354700855			H	H	0	0	general GO
immune_response (366)	OCT.01	0.354700855			H	H	M	0	general GO
visual_perception (142)	SIX3.01	0.352962963			H	H	H	0	general GO
sensory_perception (201)	SIX3.01	0.352962963		H	H	H	H	0	general GO
immune_response (366)	PDX1_G_SA/	0.352941176		H	0	0	0	0	general GO
signal_transduction (1189)	GATA2.01	0.351428571		M	0	0	0	0	general GO
sensory_perception (201)	GATA2.01	0.351428571				H	H	H	general GO
inflammatory_response (163)	GATA2.01	0.351428571		0	0	0	0	0	general GO
immune_response (366)	GATA2.01	0.351428571				H		H	general GO
G_protein_coupled_receptor_protein_signaling_	GATA2.01	0.351428571			H	H	H	H	general GO
chemotaxis (98)	GATA2.01	0.351428571		H		0	0	0	general GO
cell_surface_receptor_linked_signal_transductio	GATA2.01	0.351428571	H	M	0	0	0	0	general GO
inflammatory_response (163)	CDX1.01	0.349621212				0	0	H	general GO
immune_response (366)	CDX1.01	0.349621212		H	H	H	0	0	general GO
antimicrobial_humoral_response (87)	CDX1.01	0.349621212		M		H	H	H	general GO
sensory_perception (201)	NKX25.02	0.347222222		H	H	H	H	H	general GO
antimicrobial_humoral_response (87)	NKX25.02	0.347222222	H	H	H	0	M	0	general GO
sensory_perception (201)	FREAC2.01	0.346774194	H				H	H	general GO
inflammatory_response (163)	DLX3.01	0.34375				0	0	0	general GO
immune_response (366)	DLX3.01	0.34375			0	0	0	0	general GO
antimicrobial_humoral_response (87)	DLX3.01	0.34375		H	H	0	H	H	general GO
proteolysis (297)	HOXC13_01	0.343589744	M	0	H	H	H	H	general GO
immune_response (366)	HOXC13_01	0.343589744		H	H	H	H	H	general GO
inflammatory_response (163)	HNF1.02	0.343382353				H	H	0	general GO
sensory_perception (201)	XFD1.01	0.342857143	H		H	H	H	0	general GO
inflammatory_response (163)	XFD1.01	0.342857143			M	M	0	0	general GO
inflammatory_response (163)	ATBF1.01	0.338562092			H	H	0	0	general GO
immune_response (366)	ATBF1.01	0.338562092		H	H	H	H	H	general GO
innate_immune_response (46)	HMEF2.01	0.3375			H	H	H	0	general GO
inflammatory_response (163)	HMEF2.01	0.3375	H		H	H	0	0	general GO
signal_transduction (1189)	GATA3.01	0.335555556		0	H	0	0	0	general GO
sensory_perception (201)	GATA3.01	0.335555556				H		H	general GO
inflammatory_response (163)	GATA3.01	0.335555556		H	H	H	H	M	general GO
immune_response (366)	GATA3.01	0.335555556				H	H		general GO
G_protein_coupled_receptor_protein_signaling_	GATA3.01	0.335555556			H	H	H	0	general GO
chemotaxis (98)	GATA3.01	0.335555556		H			H	0	general GO
inflammatory_response (163)	EVI1.04	0.333333333				M	0	0	general GO
sensory_perception (201)	EVI1.02	0.333333333	H	H			H	H	general GO
sensory_perception (201)	MEIS1_HOX/	0.333333333	H		H	H	H	H	general GO
sensory_perception (201)	PBX_HOXA9_	0.333333333	H		H	H	H	H	general GO
inflammatory_response (163)	MEIS1_HOX/	0.333333333		H		H	H	H	general GO
inflammatory_response (163)	PBX_HOXA9_	0.333333333		H		H	H	H	general GO
immune_response (366)	EVI1.02	0.333333333		H	H	0	0	0	general GO
immune_response (366)	MEIS1_HOX/	0.333333333				H	H	H	general GO
immune_response (366)	PBX_HOXA9_	0.333333333				H	H	H	general GO
inflammatory_response (163)	BRN3.02	0.331934732					H	H	general GO
immune_response (366)	BRN3.02	0.331934732			H	H	H	H	general GO
sensory_perception (201)	FKHRL1.01	0.327412587				0	H	0	general GO
inflammatory_response (163)	BRN2.03	0.324242424		H		0	0	0	general GO
sensory_perception (201)	XFD2.01	0.323076923				H	H	H	general GO
inflammatory_response (163)	XFD2.01	0.323076923				M	0	0	general GO
immune_response (366)	XFD2.01	0.323076923		H	H	H	0	0	general GO
inflammatory_response (163)	OC2.01	0.320855615		H			M	M	general GO
immune_response (366)	OC2.01	0.320855615		H	H	H	H	H	general GO
inflammatory_response (163)	LMX1B.01	0.32					H	0	general GO
response_to_virus (55)	FREAC3.01	0.319230769	H	H		H	H	0	general GO

proteolysis (297)	FREAC3.01	0.319230769		0	0	0	0	0	general GO
inflammatory_response (163)	FREAC3.01	0.319230769	M	M			H	0	general GO
immune_response (366)	FREAC3.01	0.319230769		M	H	0	H	0	general GO
inflammatory_response (163)	HOXA9.01	0.318627451				H	H	H	general GO
defense_response (100)	HOXA9.01	0.318627451	0	M	0	H	H	H	general GO
innate_immune_response (46)	CART1.01	0.316599389	M		M		H	0	general GO
inflammatory_response (163)	CART1.01	0.316599389		M	M	0	0	0	general GO
immune_response (366)	CART1.01	0.316599389				0	H	0	general GO
innate_immune_response (46)	EN1.01	0.315384615	H	H		H	H	0	general GO
antimicrobial_humoral_response (87)	EN1.01	0.315384615		H	H	0	0	0	general GO
transport (940)	HNF1.03	0.313664596			H	0	0	0	general GO
sodium_ion_transport (75)	HNF1.03	0.313664596			H	0	0	0	general GO
inflammatory_response (163)	HNF1.03	0.313664596		H	H	0	0	0	general GO
blood_coagulation (70)	HNF1.03	0.313664596				0	0	H	general GO
steroid_metabolism (54)	PBX1.01	0.312770468				M	0	0	general GO
nucleosome_assembly (71)	PBX1.01	0.312770468	H				M	M	general GO
inflammatory_response (163)	PBX1.01	0.312770468		H	0	H	H	0	general GO
signal_transduction (1189)	EVI1.01	0.3125		0	H	0	0	0	general GO
sensory_perception (201)	EVI1.01	0.3125	H	H		H	H	H	general GO
inflammatory_response (163)	EVI1.01	0.3125			H	0	0	0	general GO
inflammatory_response (163)	HNF6.01	0.311564626		H	H	H	H	0	general GO
immune_response (366)	HNF6.01	0.311564626	H	H			H	H	general GO
innate_immune_response (46)	SRY.01	0.308080808	H		H	H	0	0	general GO
immune_response (366)	SRY.01	0.308080808				H	H	0	general GO
inflammatory_response (163)	BRN3.01	0.301216641			H		H	H	general GO
immune_response (366)	BRN3.01	0.301216641		H	H	H	0	0	general GO
antimicrobial_humoral_response (87)	XVENT2.01	0.300287356		H	H	H	H	0	general GO
inflammatory_response (163)	ATATA.01	0.3		H	H	H	0	0	general GO
immune_response (366)	ATATA.01	0.3		M	M	0	0	0	general GO
sensory_perception (201)	MEF2.02	0.298701452		H	H	H	H	H	general GO
muscle_development (107)	MEF2.02	0.298701452				H	0	0	general GO
inflammatory_response (163)	MEF2.02	0.298701452		H	H	H	H	0	general GO
immune_response (366)	MEF2.02	0.298701452	H		H	H	0	0	general GO
sensory_perception (201)	MEF2.03	0.298542774		H	H	H	0	H	general GO
muscle_development (107)	MEF2.03	0.298542774		H	H	0	0	0	general GO
inflammatory_response (163)	MEF2.03	0.298542774				H	H	H	general GO
immune_response (366)	MEF2.03	0.298542774			H	H	H	0	general GO
transport (940)	HNF1.01	0.296715928			H	0	0	0	general GO
steroid_metabolism (54)	HNF1.01	0.296715928	0	0		0		H	general GO
inflammatory_response (163)	DLX1.01	0.292929293			H	0	0	0	general GO
sensory_perception (201)	MEF2.05	0.292673993	H	H	H	H	H		general GO
immune_response (366)	MEF2.05	0.292673993		M	M	M	M	M	general GO
inflammatory_response (163)	RSRFC4.02	0.292371595					H	0	general GO
immune_response (366)	RSRFC4.02	0.292371595	H			H	H	H	general GO
sensory_perception (201)	CDP.02	0.290344162			H	H	H	H	general GO
pregnancy (41)	CDP.02	0.290344162	0	M	0	0	0	H	general GO
inflammatory_response (163)	CDP.02	0.290344162		M	H	H	H	H	general GO
immune_response (366)	CDP.02	0.290344162	H	H		H	H	H	general GO
immune_response (366)	MEL1_01	0.288515406					H		general GO
inflammatory_response (163)	CLOX.01	0.287721893		0	0	H	H	0	general GO
immune_response (366)	CLOX.01	0.287721893	H	H		H	H	H	general GO
sensory_perception (201)	GATA3.02	0.286666667		H	H				general GO
metabolism (241)	GATA3.02	0.286666667	0	M	0	H	0	H	general GO
inflammatory_response (163)	GATA3.02	0.286666667		H	0	H	0	0	general GO
immune_response (366)	GATA3.02	0.286666667			H	H	H	H	general GO
chemotaxis (98)	GATA3.02	0.286666667	H	0	0		0	0	general GO
inflammatory_response (163)	MEF2.04	0.286298736	H	H	H		0	0	general GO
immune_response (366)	MEF2.04	0.286298736		H	H	0	H	0	general GO
sensory_perception (201)	EVI1.05	0.28620194		H		H	H	H	general GO
inflammatory_response (163)	EVI1.05	0.28620194		0	0	0	0	0	general GO
immune_response (366)	EVI1.05	0.28620194			H	H	0	0	general GO
sensory_perception (201)	TATA.01	0.284987277			H	H	0	0	general GO
nucleosome_assembly (71)	TATA.01	0.284987277			H	H	H	0	general GO
inflammatory_response (163)	TATA.01	0.284987277				0	0	0	general GO

immune_response (366)	TATA.01	0.284987277				0	0	0	general GO
epidermis_development (59)	TATA.01	0.284987277				0	0	0	general GO
chromosome_organization_and_biogenesis (83)	TATA.01	0.284987277	H		H	H	H	0	general GO
chemotaxis (98)	TATA.01	0.284987277				0	0	0	general GO
cell_cell_signaling (268)	TATA.01	0.284987277				0	0	0	general GO
calcium_ion_homeostasis (30)	TATA.01	0.284987277			M	0	0	0	general GO
antimicrobial_humoral_response (87)	TATA.01	0.284987277		H	H	0	0	0	general GO
sensory_perception (201)	RSRFC4.01	0.28125		H	H	H	H	H	general GO
muscle_development (107)	RSRFC4.01	0.28125			H	H	0	0	general GO
inflammatory_response (163)	RSRFC4.01	0.28125				H	H	H	general GO
immune_response (366)	RSRFC4.01	0.28125				H	H	0	general GO
chemotaxis (98)	RSRFC4.01	0.28125		0	0	0	0	0	general GO
inflammatory_response (163)	TATA.02	0.276190476				0	0	H	general GO
immune_response (366)	TATA.02	0.276190476				H	0	0	general GO
chemotaxis (98)	TATA.02	0.276190476		H	H	H	H	H	general GO
cell_cell_signaling (268)	TATA.02	0.276190476				0	0	0	general GO
inflammatory_response (163)	MYT1.02	0.275624837		M	0	0	0	0	general GO
immune_response (366)	MYT1.02	0.275624837		H	H	0	0	0	general GO
homophilic_cell_adhesion (70)	MYT1.02	0.275624837		H	H	H	H	H	general GO
sensory_perception (201)	PIT1.01	0.273333333			H	0	H	H	general GO
innate_immune_response (46)	PIT1.01	0.273333333	H	0		H	H	0	general GO
inflammatory_response (163)	PIT1.01	0.273333333		H		H	H	0	general GO
immune_response (366)	PIT1.01	0.273333333				H	H	0	general GO
sensory_perception (201)	FREAC7.01	0.272727273						H	general GO
inflammatory_response (163)	FREAC7.01	0.272727273			0	M	0	0	general GO
immune_response (366)	FREAC7.01	0.272727273	H	H		0	0	0	general GO
inflammatory_response (163)	SATB1.01	0.272321429				H	H	0	general GO
inflammatory_response (163)	HFH1.01	0.268518519			H	H	0	0	general GO
sensory_perception (201)	HFH8.01	0.266025641	H	H			H	0	general GO
proteolysis (297)	HFH8.01	0.266025641			H	H	0	0	general GO
inflammatory_response (163)	HFH8.01	0.266025641		H	0	0	0	0	general GO
sensory_perception_of_smell (40)	MYT1.01	0.265925926		0	0	0	0	0	general GO
inflammatory_response (163)	MYT1.01	0.265925926		H	H	H	0	0	general GO
immune_response (366)	MYT1.01	0.265925926			H	H	0	0	general GO
sensory_perception (201)	OCT1.01	0.264074431	H	H			H	0	general GO
metabolism (241)	OCT1.01	0.264074431	M	0	0	H	0	0	general GO
inflammatory_response (163)	OCT1.01	0.264074431					M	0	general GO
sensory_perception (201)	BRN5.01	0.262032086	H		H		H	H	general GO
proteolysis (297)	BRN5.01	0.262032086		0	0	0	0	0	general GO
immune_response (366)	BRN5.01	0.262032086				0	0	0	general GO
inflammatory_response (163)	HNF3B.01	0.255731922	M	0	H	0	0	0	general GO
immune_response (366)	HNF3B.01	0.255731922			M	M	0	0	general GO
digestion (51)	HNF3B.01	0.255731922		M	0	0	0	0	general GO
innate_immune_response (46)	HFH2.01	0.25	H	M	0	0	0	0	general GO
inflammatory_response (163)	HFH2.01	0.25		H	M	0	0	0	general GO
immune_response (366)	HFH2.01	0.25				H	0	0	general GO
immune_response (366)	NKX31.01	0.247412008		H	H	0	0	0	general GO
inflammatory_response (163)	BRN2.02	0.246031746					0	0	general GO
immune_response (366)	EVI1.03	0.24340176			H	H	H	H	general GO
innate_immune_response (46)	BRIGHT.01	0.217287014	0	M	H	0	0	0	general GO
inflammatory_response (163)	BRIGHT.01	0.217287014		H			H	0	general GO
sensory_perception (201)	OCT1.06	0.216820628	H	H		H	0	0	general GO
innate_immune_response (46)	OCT1.06	0.216820628		M		M	M	0	general GO
inflammatory_response (163)	OCT1.06	0.216820628					H	H	general GO
immune_response (366)	OCT1.06	0.216820628			H	H	H	H	general GO
inflammatory_response (163)	LHX3.01	0.138047138		H	H	H	H	H	general GO

100bp Window with overlap 50bp

GO group	Motifs	motif GC	-199 - -100	-149 - -50	-99 - 0	-49 - 50	1 - 100	51 - 150	101 - 200	151 - 250	201 - 300	251 - 350	301 - 400	351 - 450	401 - 500	451 - 550	501 - 600	551 - 650	601 - 700	651 - 750	701 - 800	751 - 850	801 - 900	851 - 950	901 - 1000	GO-classes
transcription (930)	GC_rich	1	H	H	0	0	0	M				M						M	H							transcription GO
transcription (930)	poly_C	1	H	H	M	0	0	0	H				H			M	M	M	0	M		H	M	0	H	transcription GO
transcription (930)	SP1.01	0.8	0	0	0	0	0	0	0	0		M	M			M	M	0	0	M	M	M		M	0	transcription GO
transcription (930)	WT1.01	0.8	H	H	0	0	0	H	M	M	M							M	0	0	H	H	0	M		transcription GO
transcription (930)	MAZR.01	0.8	0	0		0	0	0	0			M	M				M	M	0	0	0	0	M	H	0	transcription GO
transcription (930)	ZNF202.01	0.7	H	H	0	0	0	0	H			M	0			M	M	0	0	0		0	0	0	0	transcription GO
transcription (930)	AP2.01	0.7	0	0	0	0	0	H	0	H		M	M	0		M	0	0	H	H	H	H	H			transcription GO
transcription (930)	ZF5.01	0.7	0	0	0	0	0	0	0	0		M	M	M		H	0	0	0	H	0	0	H	H	H	transcription GO
transcription (930)	NRF1_01	0.7	0	0	0	0	0	0	0	0				M	M	M	0	0	0	H	H	H	H	H		transcription GO
transcription (930)	ZBP89.01	0.7	H	H	M	0	M	M	0		M	0	M	M	H	M	M	M	0	0	0	0	0	0	0	transcription GO
transcription (930)	CKROX_01	0.7	H		M	0	M	H	0	0	H		M		H	M	M	0	0	0	0	0	0	0	0	transcription GO
transcription (930)	EGR1.02	0.7	H	H	M	0	M	H	H		M		H	H			M	M	0		H	0	H		H	transcription GO
transcription (930)	GC.01	0.7	0	0	0	0	0	0	0	0	H	M	0	0		M	M	0	0	0	0	0	M	0	0	transcription GO
transcription (930)	MAZ.01	0.7	0	0	0	0	0	0	0	H		M	0	M	M	M	M	M	0	0	0	H	H	0	0	transcription GO
transcription (930)	PLAG1_01	0.6	0	0	0	0	0	0	H		0	0	0	M	M		M	0	0	0	H	H	H	H	0	transcription GO
regulation_of_transcription_DNA_depe	GC_rich	1	H		0	0	0	M											H			H				transcription GO
regulation_of_transcription_DNA_depe	poly_C	1	H	H	M	M	0						0				M	M	0				M	M	H	transcription GO
regulation_of_transcription_DNA_depe	SP1.01	0.8	0	H	0	0	0	0	0							M	M	M	0	M	M	M				transcription GO
regulation_of_transcription_DNA_depe	WT1.01	0.8				0	0	0		M	M							M	0	0	H		M			transcription GO
regulation_of_transcription_DNA_depe	MAZR.01	0.8	H	0	H	M	0	M	0				M				M	M	M	0	0	M	M		0	transcription GO
regulation_of_transcription_DNA_depe	ZNF202.01	0.7	H	H	0	0	0	0	H			M	0			M	M	M	0	H		M	M	M	0	transcription GO
regulation_of_transcription_DNA_depe	AP2.01	0.7	0	0	0	0	0	H	M			M	M	H		M	H	H	H	H	H	H	H			transcription GO
regulation_of_transcription_DNA_depe	ZF5.01	0.7	0	0	0	0	0	0	H	M		M		M	M		H	H	H	H	H	0	H	H		transcription GO
regulation_of_transcription_DNA_depe	ZF9.01	0.7	0	0	0	0	0	0	0	H	H		M	0	H	H	H	H	M	M	M	M			0	transcription GO
regulation_of_transcription_DNA_depe	NRF1_01	0.7	0	0	0	0	0	0	M	0	M			M		0	0	H	H	H	H	H	H	H	H	transcription GO
regulation_of_transcription_DNA_depe	ZBP89.01	0.7	H	H	M	0	M	M	M						H		M	M	0	0	H	0	0	0	0	transcription GO
regulation_of_transcription_DNA_depe	CKROX_01	0.7	H			0	M	M		0							M	0	0		H	0	M	0	0	transcription GO
regulation_of_transcription_DNA_depe	EGR1.02	0.7	0		M	0	0	0						H			M	M	M		H	0				transcription GO
regulation_of_transcription_DNA_depe	EGR1.01	0.7	0	0	0	0	0	0	0	0		M	0	0	H	0	M	M	0	0	0	0	0	0	0	transcription GO
regulation_of_transcription_DNA_depe	MAZ.01	0.7	0	0	0	0	0	0	0	H		M	M	M			M	M	M	0	0	H		0	0	transcription GO
regulation_of_transcription_DNA_depe	PLAG1_01	0.6	0	M	M	0	0	0	H	0	0	M	M	M			M	M	0	0	M	H	H	H	0	transcription GO
regulation_of_transcription_DNA_depe	HES1.01	0.6	0	0	0	0	0	M	M	0	0	0	0	0	M	0	0	0	0	H	H	0		H	0	transcription GO
regulation_of_transcription_DNA_depe	MUSCLE_INI.0	0.6	0	0	0	0	0	0	0	0		0	0	M	0	0	H	H	0	0	0	M	0	0	0	transcription GO
regulation_of_transcription_DNA_depe	MUSCLE_INI.0	0.6	0	0	0	0	0	0	0	0	0	0	M		0	0	H	0	0	0	0	0	0	0	0	transcription GO
regulation_of_transcription_DNA_depe	CDE.01	0.6	0	0	0	0	0	0	0	M	M	0	M	0	H		M	M	M	0	M	M	0	H	H	transcription GO
regulation_of_transcription_DNA_depe	E2F.02	0.6	0	0	0	H	0	0	0	M	0	0	0	0	H	0	0	H	M		0	0	0	0		transcription GO
regulation_of_transcription_DNA_depe	GAGA.01	0.6		H	0	0	H	H	0	0	0	H	0	M	H	H	M	0	0	0	0	H		0	0	transcription GO
regulation_of_transcription (266)	poly_C	1	0	0	0	0	0	0	M	H			0	H	H	H	0	0	0	0	0	0	M	0	0	transcription GO
regulation_of_transcription (266)	WT1.01	0.8	0	0	0	0	0	0	0	H	M		0	0	H	M	0	0	0	0	0	0	0	0	0	transcription GO
regulation_of_transcription (266)	ZF5.01	0.7	0	0	0	0	0	0	H	M		0	0	0	0	0	0	0	0	0	0	0	0	0	0	transcription GO
regulation_of_transcription (266)	ZBP89.01	0.7	0	0	0	0	0	0	H		0	0	0	0	H	0	0	0	0	0	0	0	0	0	0	transcription GO
regulation_of_transcription (266)	CKROX_01	0.7	0	0	0	0	M	M	0	0	H		0	0	H	M	M	0	0	0	0	0	0	0	0	transcription GO
regulation_of_transcription (266)	EGR1.02	0.7	0	0	0	0	0	0		H	M	0	0	0	H	0	0	0	0	0	0	0	0	0	0	transcription GO
digestion (51)	HFH8.01	0.3	H	0	0	0		0	0	0	0	0	0	0	0	0	0	0	0	0	0	0	0	0	0	transcription GO
development (530)	poly_C	1	0	0	M	0	H				H	H	H	H	H	0	0	H	0	0	0	M	0	0	M	transcription GO
development (530)	WT1.01	0.8	H	0	0	0	0	H	0	0		H	0	H	0	0	0		0	0	H	M	0	H	0	transcription GO
development (530)	MAZR.01	0.8	0	0	0	0	0	0	H		H	H	M		H	H		0	0	0	H	H	0	H	H	transcription GO
development (530)	ZNF202.01	0.7	0	0	0	0	H	H		H	H	H	0	H	H	0	0	0	0	0	0	M	0	0	0	transcription GO
development (530)	ZBP89.01	0.7	0	0	0	0	H	H		H	H	H	0	H	H	0	M	0	0	H	H	0	M	0	0	transcription GO
development (530)	CKROX_01	0.7	0	0	0	0	H	H	H		H	H	M	H	H	0	0	0	0	0		0	0	0	0	transcription GO
development (530)	MZF1.01	0.7	0	0	0	0	H	0	H	0	0	H	H		M	H	H	0	H	H	0	0	0	H	0	transcription GO

development (530)	PLAG1_01	0.6	0	0	M	0	H	H	H	H	H	H	H		H	0	0	H	0	0	H	0	0	0	0	transcription GO
development (530)	GAGA.01	0.6	0	H	0		H	H	0		H	H			H	0	0	0	0	0	0	H	0	H	0	transcription GO
development (530)	GKLF.01	0.5	0	H		M	0	0	0	H	H	0	H	H	0	0	0	0	0	0	0	0	0	0	0	transcription GO
development (530)	MTATA.01	0.5	0	0	0	H		M	0	0	0	0	0	0	0	0	0	0	0	0	0	0	0	0	0	transcription GO
development (530)	AMEF2.01	0.4	0	0	0	M		0	0	0	0	0	0	0	0	0	0	0	0	0	0	0	0	0	0	transcription GO
development (530)	MMEF2.01	0.4	0	0	H	0		0	0	0	0	0	0	0	0	0	0	0	0	0	0	0	0	0	0	transcription GO
Wnt_receptor_signaling_pathway (66)	poly_C	1	0	0	0	0	0		H	H	H	0	0	0	0	0	0	0	0	0	0	0	0	0	H	general GO
visual_perception (142)	PTX1.01	0.4	0	0	0	0			0	0	0	0	0	0	0	0	0	0	0	0	0	0	0	0	0	general GO
visual_perception (142)	GSC.01	0.4	0	0	0	0		H	0	0	0	0	0	0	0	0	0	0	0	0	0	0	0	0	0	general GO
visual_perception (142)	CRX.01	0.4	0	0	0	0			0	0	0	0	0	0	0	0	0	0	0	0	0	0	0	0	0	general GO
visual_perception (142)	OTX2.01	0.4	0	0	0	0		0	0	0	0	0	0	0	0	0	0	0	0	0	0	0	0	0	0	general GO
visual_perception (142)	SIX3.01	0.4	0	0	0	0		H	H	H	H	0	0	H	0	0	0	0	0	0	0	0	0	0	H	general GO
visual_perception (142)	NKX31.01	0.2	0	0	0	0	0		0	0	0	0	0	0	0	0	0	0	0	0	0	0	0	0	0	general GO
transport (940)	HNF1.03	0.3	0	0	0	0			H	0	0	0	0	0	0	0	0	0	0	0	0	0	0	0	0	general GO
transport (940)	HNF1.01	0.3	0	0	0	0			0	0	0	0	0	0	0	0	0	0	0	0	0	0	0	0	0	general GO
transcription_RNA_polyII_promoter	ZNF202.01	0.7	0	0	0	0	0	0	H		M	0	0	M	0	0	0	0	0	0	0	0	0	0	0	general GO
transcription_RNA_polyII_promoter	CDE.01	0.6	0	0	0	0	0	0	0	0	0	0	0	0	0	0	0	0	0	H		0	0	0	0	general GO
transcription_RNA_polyII_promoter	PAX5.01	0.6	H	0	0	0	0	0	0	0	0	0	0	0	0	0	H		0	0	0	0	0	0	0	general GO
steroid_biosynthesis (42)	NFY.01	0.5	0	0	0	0	M		H	0	0	0	0	0	0	0	0	0	0	0	0	0	0	0	0	general GO
steroid_biosynthesis (42)	CAAT.01	0.5	0	0	0	0	M		H	0	0	0	0	0	0	0	0	0	0	0	0	0	0	0	0	general GO
steroid_biosynthesis (42)	NFY.02	0.5	0	0	0	0	0		H	0	0	0	0	0	0	0	0	0	0	0	0	0	0	0	0	general GO
sodium_ion_transport (75)	HNF1.03	0.3	0	0	0	0			0	0	0	0	0	0	0	0	0	0	0	0	0	0	0	0	0	general GO
sodium_ion_transport (75)	HNF1.01	0.3	0	0	0	0			0	0	0	0	0	0	0	0	0	0	0	0	0	0	0	0	0	general GO
small_GTPase_mediated_signal_trans	GC_rich	1	H	H	H		H	H	H	0	0	0	0	0	0	0	0	0	0	0	0	0	0	0	0	general GO
small_GTPase_mediated_signal_trans	SP1.01	0.8	H	0	M		H	H	H	0	0	0	0	0	0	0	0	0	0	0	0	0	0	0	0	general GO
small_GTPase_mediated_signal_trans	EGR1.02	0.7	H	0				H	0	0	0	0	0	0	0	0	0	0	0	0	0	0	0	0	0	general GO
signal_transduction (1189)	NFKAPPAB.02	0.6	0	0	0	0		H	0	0	0	0	0	0	0	0	0	0	0	0	0	0	0	0	0	general GO
signal_transduction (1189)	NFKAPPAB.03	0.5	H	0	0	0		0	0	0	0	0	0	0	0	0	0	0	0	0	0	0	0	0	0	general GO
signal_transduction (1189)	HIVEP1_01	0.5	0	0	0	0		H	0	0	0	0	0	0	0	M	0	0	0	0	0	0	0	0	0	general GO
signal_transduction (1189)	IRF4.01	0.4	0	0	0	0		0	0	0	0	0	0	0	0	0	0	0	0	0	0	0	0	0	0	general GO
signal_transduction (1189)	EVI1.02	0.3	0	0	0	M		H	0	0	0	0	0	0	0	0	0	0	0	0	0	0	0	0	0	general GO
signal_transduction (1189)	EVI1.01	0.3	0	0	0	M		H	0	0	0	0	0	0	0	0	0	0	0	0	0	0	0	0	0	general GO
sensory_perception (201)	NFKAPPAB.02	0.6	0	0	0	0	H		M	0	0	0	0	0	0	0	0	0	0	0	0	0	0	0	0	general GO
sensory_perception (201)	HIVEP1_01	0.5	0	0	0	H	H		0	0	0	0	0	0	0	0	0	0	0	0	0	0	0	0	0	general GO
sensory_perception (201)	GKLF_02	0.5	0	0	0	0		0	0	0	0	0	0	0	0	0	0	0	0	0	0	0	0	0	0	general GO
sensory_perception (201)	GSC.01	0.4	0	0	0	0		H	H	0	0	0	0	0	0	0	0	0	0	0	0	0	0	0	0	general GO
sensory_perception (201)	SRF.01	0.4	H	H	0	H		H	H	H	0	0	0	0	0	0	0	0	0	0	0	0	0	0	0	general GO
sensory_perception (201)	GFI1.01	0.4	0	0	0	H		0	0	0	0	H	H	0	0	0	0	0	0	0	0	0	0	0	0	general GO
sensory_perception (201)	CRX.01	0.4	0	0	0	0			0	0	H	0	0	0	0	0	0	0	0	0	0	0	0	0	0	general GO
sensory_perception (201)	XFD3.01	0.4	0	0	0	H		H	0	0	0	0	0	0	0	0	0	0	0	0	0	0	0	0	0	general GO
sensory_perception (201)	OCT1P.01	0.4	0	0	H	0	H			H	H	0	H	H	H	0	0	0	0	0	0	0	0	0	0	general GO
sensory_perception (201)	SIX3.01	0.4	0	0	0	0		H	H	0	H	H	0	H	0	0	0	0	0	0	0	0	0	0	0	general GO
sensory_perception (201)	GATA3.02	0.3	0	0	0		H	H	H	H	H	H	0	H	H	0	0	0	0	0	0	0	0	0	0	general GO
sensory_perception (201)	EVI1.05	0.3	0	0	H	H	H		H	H	0	0	0	H	H	0	0	0	0	0	0	0	0	0	0	general GO
sensory_perception (201)	TATA.01	0.3	0	0	0			H	H	0	0	0	0	0	0	0	0	0	0	0	0	0	0	0	0	general GO
response_to_virus (55)	ISRE.01	0.4	0	0	0	0	H			M	0	0	H	0	0	0	0	0	0	0	0	0	0	0	0	general GO
response_to_virus (55)	IRF2.01	0.4	0	0	0	M		H	H	0	0	H	H	H	0	0	0	0	0	0	0	0	0	0	0	general GO
response_to_virus (55)	IRF3.01	0.4	0	0	0	0	H	H		M	0	0	0	0	0	0	0	0	0	0	0	0	0	0	0	general GO
response_to_virus (55)	IRF1.01	0.4	0	0	0	M		H	H	0	0	H	H	H	0	0	0	0	H	0	0	0	0	0	0	general GO
response_to_virus (55)	TATA.01	0.3	0	0	0	H		0	0	H	0	H	H	H	H	H	H	0	0	0	0	0	0	0	0	general GO
response_to_DNA_damage_stimulus	E2F.02	0.6	0	0		M	0	0	0	0	0	M	0	0	0	0	0	0	0	0	0	0	0	0	0	general GO
response_to_DNA_damage_stimulus	E2F.03	0.5	0	H	H		0	0	0	0	0	0	0	0	0	0	0	0	0	0	0	0	0	0	0	general GO
regulation_of_transcription_from_RNA	ZNF202.01	0.7	0	0	0	0	0	0	H		0	0	0	0	0	0	0	0	0	0	0	0	0	0	0	general GO
proteolysis (297)	HNF1.03	0.3	0	0	0	M		H	0	0	0	0	0	0	0	0	0	0	0	0	0	0	0	0	0	general GO
proteolysis (297)	HNF1.01	0.3	0	0	0	M		H	0	0	0	0	0	0	0	0	0	0	0	0	0	0	0	0	0	general GO
proteolysis (297)	TATA.01	0.3	0	0	0		H	0	0	0	0	0	0	0	0	0	0	0	0	0	0	0	0	0	0	general GO
protein_transport (254)	EGR1.02	0.7	0	0		H	H	0	0	0	0	0	0	0	0	0	0	0	0	0	0	0	0	0	0	general GO
protein_targeting (37)	NRF2.01	0.6	0	0	0	M		0	0	0	0	0	0	0	0	0	0	0	0	0	0	0	0	0	0	general GO
protein_targeting (37)	ELK1.02	0.5	0	0	0		H	0	0	0	0	0	0	0	0	0	0	0	0	0	0	0	0	0	0	general GO
protein_biosynthesis (197)	GABP.01	0.6	0	0	0	H		0	0	0	0	0	0	0	0	0	0	0	0	0	0	0	0	0	0	general GO
protein_biosynthesis (197)	NRF2.01	0.6	0	0	H			M	0	0	0	0	0	0	0	0	0	0	0	0	0	0	0	0	0	general GO
protein_biosynthesis (197)	CETS1P54.01	0.6	0	0	0			0	0	0	0	0	0	0	0	0	0	0	0	0	0	0	0	0	0	general GO
protein_biosynthesis (197)	YY1.01	0.6	0	0		H	0	0	0	0	0	0	0	0	0	0	0	0	0	0	0	0	0	0	0	general GO
protein_biosynthesis (197)	ELK1.02	0.5	0	0	H			H	0	0	0	0	0	0	0	0	0	0	0	0	0	0	0	0	0	general GO

protein_biosynthesis (197)	ELF2.01	0.5	0	0	H			0	0	0	0	0	0	0	0	0	0	0	0	0	0	0	0	0	0	general GO
protein_biosynthesis (197)	FLI.01	0.5	0	0	H				0	0	0	0	0	0	0	0	0	0	0	0	0	0	0	0	0	general GO
protein_biosynthesis (197)	STAF.01	0.5	0	0	0	H		0	0	0	0	0	0	0	0	0	0	0	0	0	0	0	0	0	0	general GO
protein_biosynthesis (197)	STAT1.01	0.5	0	0	0	H		H	0	0	0	0	0	0	0	0	0	0	0	0	0	0	0	0	0	general GO
protein_biosynthesis (197)	ELK1.01	0.5	0	0	H			0	0	0	0	0	0	0	0	0	0	0	0	0	0	0	0	0	0	general GO
protein_biosynthesis (197)	AARE.01	0.4	0	0	0		M	0	0	0	0	0	0	0	0	0	0	0	0	0	0	0	0	0	0	general GO
nucleosome_assembly (71)	NFY.01	0.5	0	0	0	0			H	H	H	0	0	0	0	0	0	0	0	0	0	0	0	0	0	general GO
nucleosome_assembly (71)	CAAT.01	0.5	0	0	0	0	0	H		H	0	0	0	0	0	0	0	H	0	0	0	0	0	0	0	general GO
nucleosome_assembly (71)	NFY.02	0.5	0	0	0	0	H		H	H	H	0	0	0	0	0	0	0	0	0	0	0	0	0	0	general GO
nucleosome_assembly (71)	NFY.03	0.5	0	0	0	0	H	H		H	0	0	0	0	0	0	0	0	0	0	0	0	0	0	0	general GO
nucleosome_assembly (71)	TATA.01	0.3	0	0	0	H		H	0	H	H	H	0	0	0	0	0	0	0	0	0	0	0	0	0	general GO
nervous_system_development (225)	CKROX_01	0.7	0	0	0	0	H	H	H	0	0	0	0	0	H	0	0	0	0	0	0	0		0	0	general GO
negative_regulation_of_cell_proliferati	TATA.01	0.3	0	0	0	0		0	0	0	0	0	0	0	0	0	0	0	0	0	0	0	0	0	0	general GO
muscle_development (107)	SRF.02	0.5	0	0	0	0	H			H	H	0	0	0	0	0	0	0	0	0	0	0	0	0	0	general GO
muscle_development (107)	MTATA.01	0.5	0	0	0			0	0	0	0	0	0	0	0	0	0	0	0	0	0	0	0	0	0	general GO
muscle_development (107)	SRF.03	0.5	0	0	0	0	H			H		0	0	0	0	0	0	0	0	0	0	0	0	0	0	general GO
muscle_development (107)	SRF.01	0.4	0	0	0	0	H			0	0	0	0	0	0	0	0	0	0	0	0	0	0	0	0	general GO
mitosis (86)	NFY.01	0.5	0	0	0	0			M	H	H	0	0	0	0	0	0	0	0	0	0	0	0	0	0	general GO
mitosis (86)	CAAT.01	0.5	0	0	0	0			0	0	0	0	0	0	0	0	0	0	0	0	0	0	0	0	0	general GO
mitosis (86)	NFY.02	0.5	0	0	0	0		M	M	0	0	0	0	0	0	0	0	0	0	0	0	0	0	0	0	general GO
mitosis (86)	CHR.01	0.4	0	0	H			0	0	0	0	0	0	0	0	0	0	0	0	0	0	0	0	0	0	general GO
innate_immune_response (46)	BRN4.01	0.4	H		0	0	0	0	0	0	0	0	0	0	0	0	0	H	0	0	0	0	0	0	0	general GO
inflammatory_response (163)	NFKAPPAB.01	0.6	0	0	0	0				0	0	0	0	0	0	0	0	0	0	0	0	0	0	0	0	general GO
inflammatory_response (163)	NFKAPPAB.02	0.6	0	0	0	0	H			H	0	0	0	0	0	0	0	0	0	0	0	0	0	0	0	general GO
inflammatory_response (163)	NFKAPPAB65.	0.6	0	0	0	H				0	0	0	0	0	0	0	0	0	0	0	0	0	0	0	0	general GO
inflammatory_response (163)	CREL.01	0.5	0	0	0	H			M	0	0	0	0	0	0	0	0	0	0	0	0	0	0	0	0	general GO
inflammatory_response (163)	NFKAPPAB.03	0.5	0	0	0	0				H	0	0	0	0	0	0	0	0	0	0	0	0	0	0	0	general GO
inflammatory_response (163)	HIVEP1_01	0.5	0	0	0	H	H			H	0	0	0	0	0	0	0	0	0	0	0	0	0	0	0	general GO
inflammatory_response (163)	ETS1.01	0.5	0	0	0	0	0	0		0	0	0	0	0	0	0	0	0	0	0	0	0	0	0	0	general GO
inflammatory_response (163)	PU1.01	0.5	0	0	0	0	0		H	0	0	0	0	0	0	0	0	0	0	0	0	0	0	0	0	general GO
inflammatory_response (163)	ETS2.01	0.5	0	0	0	0	H	H		0	0	0	0	0	0	0	0	0	0	0	0	0	0	0	0	general GO
inflammatory_response (163)	COMP1.01	0.5	0	0	0		0	0	0	0	0	0	0	0	0	0	0	0	0	0	0	0	0	0	0	general GO
inflammatory_response (163)	TCF11MAFG.0	0.4	0	0		H	H	H	0	0	0	0	0	0	0	0	0	0	0	0	0	0	0	0	0	general GO
inflammatory_response (163)	ISRE.01	0.4	0	0	0	0	H		M	0	0	0	0	0	0	0	0	0	0	0	0	0	0	0	0	general GO
inflammatory_response (163)	IRF3.01	0.4	0	0	0	0	H		M	0	0	0	0	0	0	0	0	0	0	0	0	0	0	0	0	general GO
inflammatory_response (163)	SRF.01	0.4	0	0	0	H		H	0	0	0	0	0	0	0	0	0	0	0	0	0	0	0	0	0	general GO
inflammatory_response (163)	CEBPB.01	0.4	0	0	0	0	H			H	0	H	H	0	0	0	0	0	0	0	0	0	0	0	0	general GO
inflammatory_response (163)	AARE.01	0.4	0	0	0	0			0	0	0	0	0	0	0	0	0	0	0	0	0	0	0	0	0	general GO
inflammatory_response (163)	BRN2.01	0.4	0	0	0	0	0			H	0	0	0	0	0	0	0	0	0	0	0	0	0	0	0	general GO
inflammatory_response (163)	IRF4.01	0.4	0		0	0	0	0	M	0	H	0	0	0	0	0	0	0	0	0	0	0	0	0	0	general GO
inflammatory_response (163)	NFAT.01	0.4	0	0	0	0	M				0	0	0	0	0	0	0	0	0	0	0	0	0	0	0	general GO
inflammatory_response (163)	CDX2.01	0.4	0	0	0	H	H	H		0	0	0	0	0	0	0	H	H	0	0	0	0	0	0	0	general GO
inflammatory_response (163)	MMEF2.01	0.4	0	0	0	H		0	H	H	0	0	0	0	0	0	0	0	0	0	0	0	0	0	0	general GO
inflammatory_response (163)	IRF7.01	0.4	M	0	0	0	H		0	0	0	0	0	0	0	0	0	0	0	0	0	0	0	0	0	general GO
inflammatory_response (163)	CDX1.01	0.3	M	0	0	0		0	0	0	0	0	0	0	0	0	0	0	0	0	0	0	0	0	0	general GO
inflammatory_response (163)	HOXC13_01	0.3	0	0	0		0	0	H	H	0	0	0	0	0	0	0	0	0	0	0	0	0	0	0	general GO
inflammatory_response (163)	MEF2.05	0.3	0	0	0	H		0	H	0	0	0	0	0	0	0	0	0	0	0	0	0	0	0	0	general GO
inflammatory_response (163)	RSRFC4.02	0.3	0	0	0	H		0	0	0	0	0	0	0	0	0	0	0	0	0	0	0	0	0	0	general GO
inflammatory_response (163)	TATA.01	0.3	0	0	0			M	H	0	0	0	0	0	0	0	0	0	0	0	0	0	0	0	0	general GO
inflammatory_response (163)	TATA.02	0.3	0	0	0			M	0	0	0	0	0	0	0	0	0	0	0	0	0	0	0	0	0	general GO
inflammatory_response (163)	PIT1.01	0.3	0	H	0	H		H	H	0	0	0	0	0	0	0	0	0	0	0	0	0	0	0	0	general GO
inflammatory_response (163)	OCT1.01	0.3	H	0	0	0	0		0	H	H	0	0	0	0	0	0	0	0	0	0	0	0	0	0	general GO
inflammatory_response (163)	OCT1.06	0.2	0	0	0	0	0		H	H	0	0	0	0	0	0	0	0	0	0	0	0	0	0	0	general GO
inflammatory_response (163)	poly_A	0	0	0	0	0	H		0	0	0	0	0	0	0	0	0	0	0	0	0	0	0	0	0	general GO
immune_response (366)	NFKAPPAB.01	0.6	0	0	0	0			M	0	0	0	0	0	0	0	0	0	0	0	0	0	0	0	0	general GO
immune_response (366)	NFKAPPAB.02	0.6	0	0	0	0			M	H	0	0	0	0	H	0	0	0	0	0	0	0	0	0	0	general GO
immune_response (366)	NFKAPPAB65.	0.6	0	0	0	H				0	0	0	0	0	H	H	0	0	0	0	0	0	0	0	0	general GO
immune_response (366)	CREL.01	0.5	0	0	0	H			0	0	0	0	0	0	0	0	0	0	0	0	0	0	0	0	0	general GO
immune_response (366)	NFKAPPAB.03	0.5	0	0	0	H				H	0	0	0	0	0	0	0	0	0	0	0	0	0	0	0	general GO
immune_response (366)	HIVEP1_01	0.5	0	0	0	H			H	0	0	0	0	0	0	0	0	0	0	0	0	0	0	0	0	general GO
immune_response (366)	ETS1.01	0.5	0	0	0	H		H	H	H	0	0	0	0	0	0	0	0	0	0	0	0	0	0	0	general GO
immune_response (366)	AML3.01	0.5	0	0	H	0	M			H	0	0	0	0	0	0	H	0	0	0	0	0	0	0	0	general GO
immune_response (366)	PU1.01	0.5	0	0	0	0			0	0	0	0	0	0	0	0	0	0	0	0	0	0	0	0	0	general GO
immune_response (366)	ETS2.01	0.5	0	0	H	H			H	H	0	0	0	0	0	0	0	0	0	0	0	0	0	0	0	general GO

immune_response (366)	GRE.01	0.5	0	0	H		0	0	0	0	H	0	0	0	0	0	0	0	0	0	0	0	0	0	0	general GO
immune_response (366)	PPARA.01	0.5		M	M	0	0	0	0	0	0	H	0	0	0	0	0	0	0	0	0	0	0	0	0	general GO
immune_response (366)	NFE2L2.01	0.5	0	0	H	0			M	0	0	0	H	0	0	0	0	0	0	0	0	0	0	0	0	general GO
immune_response (366)	STAT6.01	0.5	0	0	H	0	H	H		0	0	H	0	0	0	0	0	0	0	0	0	0	0	0	0	general GO
immune_response (366)	PXRCAR.01	0.5	0	0	0	0	H	0	M		0	0	0	0	0	0	0	0	0	0	0	0	0	0	0	general GO
immune_response (366)	RTR.01	0.4	0	0	M		H	H	0	0	0	0	0	0	0	0	H	0	0	0	0	0	0	0	0	general GO
immune_response (366)	MYT1L.01	0.4	0	0	0	0	H			0	0	0	0	0	0	0	0	0	0	0	0	0	0	0	0	general GO
immune_response (366)	STAT.01	0.4	0	0	0	0	0		H	H	0	0	0	0	0	0	0	0	0	0	0	0	0	0	0	general GO
immune_response (366)	PRE.01	0.4	0	H	H		H	H	0	0	H	0	0	H	0	0	0	0	0	0	0	0	0	0	0	general GO
immune_response (366)	BCL6.02	0.4	0	0	0	0	0			H	0	0	0	0	0	0	0	0	0	0	0	0	0	0	0	general GO
immune_response (366)	TCF11MAFG.0	0.4	0	0	0	0	0		H	H	H	0	0	0	0	0	0	0	0	0	0	0	0	0	0	general GO
immune_response (366)	AP1.01	0.4	0	H	0	0	M		0	0	0	H	H	0	0	0	H	0	0	0	0	0	0	0	0	general GO
immune_response (366)	ISRE.01	0.4	0	0	M					H	0	0	0	0	0	0	0	0	0	0	0	0	0	0	0	general GO
immune_response (366)	IRF2.01	0.4	0	0						0	0	0	0	0	0	0	0	0	0	0	0	0	0	0	0	general GO
immune_response (366)	IRF3.01	0.4	0	0	0					M	0	0	0	0	0	0	0	0	0	0	0	0	0	0	0	general GO
immune_response (366)	PRDM1.01	0.4	0	0	0				H	0	0	0	0	0	0	0	0	0	0	0	0	0	0	0	0	general GO
immune_response (366)	HMGIY.01	0.4	0	0	0	H				0	0	0	0	0	0	0	0	0	0	0	0	0	0	0	0	general GO
immune_response (366)	RORA1.01	0.4	0	0		M	0	0	0	0	0	0	0	0	0	0	H	0	0	0	0	0	0	0	0	general GO
immune_response (366)	PSE_02	0.4	0	0	0	M	0			0	0	0	0	H	0	0	0	0	0	0	0	0	0	0	0	general GO
immune_response (366)	MTBF.01	0.4		H	0	H	H	H	0	0	0	0	0	0	0	0	0	0	0	0	0	0	0	0	0	general GO
immune_response (366)	IRF1.01	0.4	0	0						M	0	0	0	0	0	0	0	0	0	0	0	0	0	0	0	general GO
immune_response (366)	OCT1.05	0.4	H	0	0	H	H		H	H	H	H	0	0	0	0	0	0	0	0	0	0	0	0	0	general GO
immune_response (366)	AARE.01	0.4	0	0	0	H		H	H	H	H	0	0	0	0	0	0	0	0	0	0	0	0	0	0	general GO
immune_response (366)	LTATA_01	0.4	H	0	0		H	H	H	0	0	0	0	0	0	0	0	0	0	0	0	0	0	0	0	general GO
immune_response (366)	IRF4.01	0.4	H	H	M				H	0	0	H	0	M	0	0	0	0	0	0	0	0	0	0	0	general GO
immune_response (366)	STAT5.01	0.4	0	0	0	H	H			H	H	0	0	H	0	0	0	0	0	0	0	0	0	0	0	general GO
immune_response (366)	BCL6.01	0.4	0	0	0	0	0			H	0	H	M	0	0	0	0	0	0	0	0	0	0	0	0	general GO
immune_response (366)	OCT1.04	0.4				H	0	H	H	0	0	0	H	0	0	0	0	0	0	0	0	0	0	0	0	general GO
immune_response (366)	GFI1B.01	0.4	0	0	0	0	0	0		H	0	0	0	0	0	0	0	0	0	0	0	0	0	0	0	general GO
immune_response (366)	AMEF2.01	0.4	H	0	0	H	H	M		0	0	0	0	0	0	0	0	0	0	0	0	0	0	0	0	general GO
immune_response (366)	NFAT.01	0.4	M	0	0	M			H	0	0	0	0	0	0	0	0	0	0	0	0	0	0	0	0	general GO
immune_response (366)	GATA1.05	0.4		0	0	H	H	0	0	0	0	H	0	0	0	0	0	0	0	0	0	0	0	0	0	general GO
immune_response (366)	IRF7.01	0.4	H	H	H					H	0	0	0	0	0	0	0	0	0	0	0	0	0	0	0	general GO
immune_response (366)	OCT.01	0.4	H			H		H	0	0	0	0	0	0	0	0	0	0	0	0	0	0	0	0	0	general GO
immune_response (366)	CDX1.01	0.3	H	H	0	H		H	H	0	0	0	0	0	0	0	0	0	0	0	0	0	0	0	0	general GO
immune_response (366)	OCT1.02	0.3	H	M		H	0	0	0	0	0	0	0	0	H	0	0	0	0	0	0	0	0	0	0	general GO
immune_response (366)	HOXC13_01	0.3	0	0	0		H	0	0	0	0	H	0	0	0	0	0	0	0	0	0	0	0	0	0	general GO
immune_response (366)	EVI1.04	0.3	0	0	0	H	H		0	0	0	0	0	0	0	0	0	0	0	0	0	0	0	0	0	general GO
immune_response (366)	EVI1.02	0.3	0	0	M	M	H	0	H	H	H	0	0		0	0	0	0	0	0	0	0	0	0	0	general GO
immune_response (366)	XFD2.01	0.3	H	0	0	M		H	H	H	0	0	0	0	0	0	0	0	0	0	0	0	0	0	0	general GO
immune_response (366)	CABL.01	0.3	0	0	0	0	0		H	0	0	0	0	0	0	0	0	0	0	0	0	0	0	0	0	general GO
immune_response (366)	EVI1.01	0.3	0	0	0		H	0	0	0	0	0	0	0	0	0	0	0	0	0	0	0	0	0	0	general GO
immune_response (366)	MEF2.02	0.3	0	0	0			0	0	0	0	0	0	0	0	0	0	0	0	0	0	0	0	0	0	general GO
immune_response (366)	MEF2.03	0.3	H	0	0		H	H	H	0	0	0	0	0	0	0	0	0	0	0	0	0	0	0	0	general GO
immune_response (366)	HNF1.01	0.3		0	0	H	H	H	H	0	0	0	0	0	0	0	0	0	0	0	0	0	0	0	0	general GO
immune_response (366)	MEF2.05	0.3	H	0	0	M		H	H	0	0	0	0	0	0	0	0	0	0	0	0	0	0	0	0	general GO
immune_response (366)	RSRFC4.02	0.3	0	0	0			H	H	0	0	0	0	0	0	0	0	0	0	0	0	0	0	0	0	general GO
immune_response (366)	MEL1_01	0.3		0	0	M	H	H	H	H	H	0	0	M	0	0	0	0	0	0	0	0	H	0	0	general GO
immune_response (366)	TATA.01	0.3	H	0	0			0	H	H	0	0	0	0	0	0	0	0	0	0	0	0	0	0	0	general GO
immune_response (366)	RSRFC4.01	0.3	0	0	0			H	H	0	0	0	0	0	0	0	0	0	0	0	0	0	0	0	0	general GO
immune_response (366)	TATA.02	0.3	0	0	0			H	H	0	0	0	0	0	0	0	0	0	0	0	0	0	0	0	0	general GO
immune_response (366)	PIT1.01	0.3	H	0	0			H	0	0	0	0	0	0	0	0	0	0	0	H	0	0	0	0	0	general GO
immune_response (366)	NKX31.01	0.2	H	0	0	0	M		H	0	0	0	0	0	0	0	0	0	0	0	0	0	0	0	0	general GO
immune_response (366)	EVI1.03	0.2		0	0	M		0	0	H	H	0	0		0	0	0	0	0	0	0	0	0	0	0	general GO
immune_response (366)	OCT1.06	0.2	H	M		H	H		H	H	0	0	0	0	H	0	0	0	0	0	0	0	0	0	0	general GO
immune_response (366)	poly_A	0	H	0	0	H				0	0	0	0	0	0	0	0	0	0	0	0	0	0	0	0	general GO
homophilic_cell_adhesion (70)	NFAT.01	0.4	0	0	0	0		0	0	0	0	0	0	0	0	0	0	0	0	0	0	0	0	0	0	general GO
G_protein_coupled_receptor_protein_	TCF11MAFG.0	0.4	0	0	0	0	H		M	0	0	H	0	0	H	H	0	0	0	0	0	0	0	0	0	general GO
G_protein_coupled_receptor_protein_	EVI1.02	0.3	0	0	0	H			0	0	0	0	0	0	0	0	0	0	0	0	0	0	0	0	0	general GO
G_protein_coupled_receptor_protein_	EVI1.01	0.3	H	0	0	H			H	H	0	0	0	0	0	0	0	H	0	0	0	0	0	0	0	general GO
G_protein_coupled_receptor_protein_	EVI1.03	0.2	H	0	H	H			0	0	0	0	0	0	0	0	0	0	0	0	0	0	0	0	0	general GO
epidermis_development (59)	LTATA_01	0.4	0	0	0	H		0	0	0	0	0	0	0	0	0	0	0	0	0	0	0	0	0	0	general GO
epidermis_development (59)	TATA.01	0.3	0	0	0			0	0	0	0	0	0	0	0	0	0	0	0	0	0	0	0	0	0	general GO
epidermis_development (59)	TATA.02	0.3	0	0	0	H		0	0	0	0	0	0	0	0	0	0	0	0	0	0	0	0	0	0	general GO

DNA_repair (148)	E2F.02	0.6	0	0			0	0	0	0	0	M	0	0	0	0	0	0	0	0	0	0	0	0	0	general GO
DNA_repair (148)	E2F.03	0.5	0	H	H		H	0	0	H	0	0	0	0	0	0	0	0	0	0	0	0	0	0	0	general GO
digestion (51)	TATA.01	0.3	0	0	0		M	0	0	0	0	0	0	0	0	0	0	0	0	0	0	0	0	0	0	general GO
digestion (51)	TATA.02	0.3	0	0	0		H	0	0	0	0	0	0	0	0	0	0	0	0	0	0	0	0	0	0	general GO
defense_response (100)	ISRE.01	0.4	0	0	0	0	H		H	0	0	0	0	0	0	0	0	0	0	0	0	0	0	0	0	general GO
defense_response (100)	IRF1.01	0.4	0	0	0	0		H	H	0	0	0	H	H	0	0	0	0	0	0	0	0	0	0	0	general GO
chromosome_organization_and_bioge	CAAT.01	0.5	0	0	0	0	0	H	H		0	0	0	0	0	0	0	0	0	0	0	0	0	0	0	general GO
chromosome_organization_and_bioge	NFY.02	0.5	0	0	0	0	H	H	H		H	0	0	0	0	0	0	0	0	0	0	0	0	0	0	general GO
chromosome_organization_and_bioge	MTATA.01	0.5	0	0	0		H	H	0	H	H	0	0	0	0	0	0	0	0	0	0	0	0	0	0	general GO
chromosome_organization_and_bioge	NFY.03	0.5	0	0	0	0	H	H	H		0	0	0	0	0	0	0	0	0	0	0	0	0	0	0	general GO
chromosome_organization_and_bioge	OCT1.04	0.4	0	0	0	0	H		H	H	0	0	0	0	0	0	0	0	0	0	0	0	0	0	0	general GO
chromosome_organization_and_bioge	TATA.01	0.3	0	0	0			H	0	H	H	H	0	0	0	0	0	0	0	0	0	0	0	0	0	general GO
chromatin_modification (53)	AP2.01	0.7	0	0	0	0	0	0	0	0	0	0	0	0	0		H	0	0	0	0	0	0	0	0	general GO
chemotaxis (98)	NFKAPPAB.01	0.6	0	0	0	0	0		M	0	0	0	0	0	0	0	0	0	0	0	0	0	0	0	0	general GO
chemotaxis (98)	NFKAPPAB.02	0.6	0	0	0	0	0		M	0	0	0	0	0	0	0	0	0	0	0	0	0	0	0	0	general GO
chemotaxis (98)	NFKAPPAB65.	0.6	0	0	0	0	H			0	0	0	0	0	0	0	0	0	0	0	0	0	0	0	0	general GO
chemotaxis (98)	CREL.01	0.5	0	0	0	0	H		M	0	0	0	0	0	0	0	0	0	0	0	0	0	0	0	0	general GO
chemotaxis (98)	NFKAPPAB.03	0.5	0	0	0	0	H			H	0	0	0	0	0	0	0	0	0	0	0	0	0	0	0	general GO
chemotaxis (98)	HIVEP1_01	0.5	0	0	0	0	H			0	0	0	0	0	0	0	0	0	0	0	0	0	0	0	0	general GO
chemotaxis (98)	IK3.01	0.5	0	0	0	0	0		M	0	0	0	0	0	0	0	0	0	0	0	0	0	0	0	0	general GO
chemotaxis (98)	HMGIY.01	0.4	0	0	0	0	H		0	H	0	0	0	H	0	0	0	0	0	0	0	0	0	0	0	general GO
chemotaxis (98)	TATA.01	0.3	0	0	0			0	0	0	0	0	0	0	0	0	0	0	0	0	0	0	0	0	0	general GO
cellular_defense_response (68)	ETS1.01	0.5	0	0	0	0		0	0	0	0	0	0	0	0	0	0	0	0	0	0	0	0	0	0	general GO
cell_motility (106)	TATA.02	0.3	0	0	H	M		0	0	0	0	0	0	0	0	0	0	0	0	0	0	0	0	0	0	general GO
cell_division (103)	NFY.01	0.5	0	0	0	0	0	0			H	0	0	0	0	0	0	0	0	0	0	0	0	0	0	general GO
cell_division (103)	CHR.01	0.4	0	0	H			0	0	0	0	0	0	0	0	0	0	0	0	0	0	0	0	0	0	general GO
cell_differentiation (192)	TATA.01	0.3	0	0	0	H		0	0	0	0	0	0	0	0	0	0	0	0	0	0	0	0	0	0	general GO
cell_cycle (273)	E2F.02	0.6	0	0	0		0	0	0	0	0	H	0	0	0	0	0	0	0	0	0	0	0	0	0	general GO
cell_cycle (273)	NFY.01	0.5	0	0	0	H	H			0	0	0	0	0	0	0	0	0	0	0	0	0	0	0	0	general GO
cell_cycle (273)	CAAT.01	0.5	0	0	0	H	0		M	0	0	0	0	0	0	0	0	0	0	0	0	0	0	0	0	general GO
cell_cell_signaling (268)	NFKAPPAB.02	0.6	0	0	0	H	H		0	0	0	0	0	0	0	0	0	0	0	0	0	0	0	0	0	general GO
cell_cell_signaling (268)	CREL.01	0.5	0	0	0	0	H		0	0	0	0	0	0	0	0	0	0	0	0	0	0	0	0	0	general GO
cell_cell_signaling (268)	HIVEP1_01	0.5	0	0	0	H	H		0	0	0	0	0	0	0	0	0	0	0	0	0	0	0	0	0	general GO
cell_cell_signaling (268)	MTATA.01	0.5	0	0	0	H		0	0	0	0	0	0	0	0	0	0	0	0	0	0	0	0	0	0	general GO
cell_cell_signaling (268)	LTATA_01	0.4	0	0	0	H		0	0	0	0	0	0	0	0	0	0	0	0	0	0	0	0	0	0	general GO
cell_cell_signaling (268)	ATATA.01	0.3	0	0	0		M	0	0	0	0	0	0	0	0	0	0	0	0	0	0	0	0	0	0	general GO
cell_cell_signaling (268)	TATA.01	0.3	0	0	H			0	0	0	0	0	0	0	0	0	0	0	0	0	0	0	0	0	0	general GO
cell_cell_signaling (268)	TATA.02	0.3	0	0	0			0	0	0	0	0	0	0	0	0	0	0	0	0	0	0	0	0	0	general GO
calcium_ion_homeostasis (30)	TATA.01	0.3	0	0	0	H		0	0	0	0	0	0	0	0	0	0	0	0	0	0	0	0	0	0	general GO
blood_coagulation (70)	HNF1.03	0.3	M	0	0	0		H	0	0	0	0	0	0	0	0	0	0	0	0	0	0	0	0	0	general GO
blood_coagulation (70)	SATB1.01	0.3			H	0	0	0	0	0	0	0	0	0	0	0	0	0	0	0	0	0	0	0	0	general GO
biosynthesis (32)	NFY.02	0.5	0	0	0	0	0		H	0	0	0	0	0	0	0	0	0	0	0	0	0	0	0	0	general GO
antimicrobial_humoral_response (87)	ISL1.01	0.4	0	0	0		H	0	0	0	H	0	0	0	0	H	0	0	0	0	0	0	0	0	0	general GO
antimicrobial_humoral_response (87)	GATA1.04	0.4	0	0	H	0	0		H	H	0	0	0	0	0	0	0	0	0	0	0	0	0	0	0	general GO
