# Supplementary material for: Wide-Scale Analysis of Human Functional Transcription Factor Binding Reveals a Strong Bias towards the Transcription Start Site
Source: PLoS One. 2007 Aug 29;2(8):e807. doi: 10.1371/journal.pone.0000807 (PMC1950076; doi:10.1371/journal.pone.0000807)
Supplement: Table S3 — Analysis of conserved BS in human mouse and rat. 39829 conserved BS in human mouse and rat from 409 Transfac PSSMs (v8.3 created by Biobase) (Wingender et al. 2000) were downloaded from UCSC browser (Kent et al. 2002). For each motif (first column) the number of BS found in the first 3000bp from the closest downstream TSS is given in the second column, the number of BSs found in the first 200bp upstream of the TSS are presented in the third column and the corresponding percentage and P-value for this over-representation (derived using the binomial distribution) are given in the fourth and fifth columns (0.03 MB PDF) [file pone.0000807.s006.pdf]

Motifs name	number of BM (3000bp)	BM (location<200)	precent in 200bp	p-value
V$AP2_Q6_01	2	2	100%	0.004444
V$ELK1_02	56	47	84%	1.11E-15
V$NRF2_01	81	66	81%	1.11E-15
V$GABP_B	147	114	78%	2.22E-15
V$USF_Q6_01	44	34	77%	6.66E-16
V$USF_01	79	61	77%	8.88E-16
V$TATA_01	146	110	75%	2E-15
V$E2F1_Q6	4	3	75%	0.001126
V$NFY_01	77	57	74%	1.55E-15
V$NRF1_Q6	194	140	72%	2.44E-15
V$STRA13_01	136	98	72%	2E-15
V$NFY_Q6_01	32	23	72%	6.66E-16
V$CREB_01	158	113	72%	2.66E-15
V$SREBP1_01	166	118	71%	2.44E-15
V$CREB_Q4	240	166	69%	3.33E-15
V$CREBATF_Q6	111	76	68%	2E-15
V$CREBP1CJUN_0	339	232	68%	5.55E-15
V$ARNT_02	161	110	68%	2.22E-15
V$MYCMAX_03	50	34	68%	8.88E-16
V$KROX_Q6	28	19	68%	4.44E-16
V$CREB_Q4_01	91	61	67%	1.55E-15
V$CREBP1_Q2	289	193	67%	4E-15
V$ARNT_01	30	20	67%	4.44E-16
V$CREB_Q2_01	222	143	64%	3.77E-15
V$CREBP1_01	375	240	64%	6.44E-15
V$ELK1_01	77	49	64%	1.55E-15
V$ATF_B	238	151	63%	3.55E-15
V$E4F1_Q6	228	144	63%	3.55E-15
V$NFY_C	238	148	62%	3.55E-15
V$ALPHACP1_01	209	129	62%	3.11E-15
V$ATF1_Q6	179	109	61%	2.66E-15
V$USF_Q6	52	31	60%	1.11E-15
V$ATF3_Q6	239	140	59%	3.33E-15
V$ATF_01	157	91	58%	2.89E-15
V$EGR3_01	79	44	56%	8.88E-16
V$E2F1_Q3_01	20	11	55%	1.1E-08
V$ATF4_Q2	159	87	55%	2.44E-15
V$CREB_Q2	143	77	54%	2.44E-15
V$CREB_02	94	50	53%	1.55E-15
V$STAT1_01	79	41	52%	8.88E-16
V$ATF6_01	99	50	51%	1.55E-15
V$STAT3_01	103	52	50%	1.78E-15
V$USF_02	4	2	50%	0.024356
V$ZF5_B	123	61	50%	1.78E-15
V$NGFIC_01	109	54	50%	2E-15
V$TAXCREB_01	87	43	49%	1.11E-15
V$TEL2_Q6	90	44	49%	1.55E-15
V$PAX5_02	33	16	48%	5.92E-11
V$PAX4_01	31	15	48%	2.45E-10
V$E2F_Q4_01	29	14	48%	1.02E-09

V$E2F_01	131	63	48%	2.44E-15
V$MIF1_01	138	66	48%	2E-15
V$TATA_C	141	67	48%	2E-15
V$NERF_Q2	80	38	48%	1.33E-15
V$NMYC_01	19	9	47%	1.3E-06
V$E2F_Q3	98	46	47%	1.55E-15
V$E2F_Q3_01	32	15	47%	4.32E-10
V$RFX1_02	235	110	47%	3.77E-15
V$ETS1_B	90	42	47%	1.55E-15
V$E2F1DP1RB_01	129	60	47%	2.22E-15
V$AHRARNT_02	54	25	46%	0
V$AHR_Q5	78	36	46%	1.33E-15
V$E2F_02	104	48	46%	1.78E-15
V$E2F_Q6_01	42	19	45%	4.49E-12
V$E2F4DP2_01	166	75	45%	2.44E-15
V$MAX_01	31	14	45%	3.06E-09
V$CP2_02	20	9	45%	2.22E-06
V$E2F4DP1_01	109	49	45%	2E-15
V$MAF_Q6	69	31	45%	1.33E-15
V$AP2_Q6	58	26	45%	4.44E-16
V$CMYB_01	76	34	45%	1.11E-15
V$E2F1DP2_01	174	77	44%	3.11E-15
V$EGR2_01	100	44	44%	1.78E-15
V$E2F_03	69	30	43%	1.33E-15
V$YY1_Q6	171	74	43%	2.66E-15
V$E2F1DP1_01	140	60	43%	1.78E-15
V$TCF1P_Q6	85	36	42%	1.55E-15
V$SP3_Q3	78	33	42%	1.33E-15
V$EGR_Q6	24	10	42%	1.42E-06
V$NFMUE1_Q6	149	62	42%	2.22E-15
V$EGR1_01	94	39	41%	1.55E-15
V$RFX1_01	156	64	41%	2.89E-15
V$E2F_Q4	61	25	41%	3.12E-14
V$STAT_01	81	33	41%	1.11E-15
V$SPZ1_01	37	15	41%	5.19E-09
V$YY1_02	168	68	40%	2.66E-15
V$MYB_Q3	25	10	40%	2.23E-06
V$PAX5_01	15	6	40%	0.000259
V$PAX4_03	10	4	40%	0.002992
V$NFY_Q6	5	2	40%	0.03881
V$MZF1_02	101	40	40%	1.78E-15
V$P300_01	33	13	39%	8.24E-08
V$TAXCREB_02	84	33	39%	1.33E-15
V$TFIII_Q6	140	55	39%	1.78E-15
V$EFC_Q6	107	42	39%	2E-15
V$YY1_01	95	37	39%	1.33E-15
V$PAX2_01	62	24	39%	4.68E-13
V$ELF1_Q6	75	29	39%	2.22E-16
V$MYCMAX_01	57	22	39%	4.67E-12
V$RFX_Q6	78	30	38%	6.66E-16
V$MYCMAX_B	13	5	38%	0.001076
V$SRF_Q4	34	13	38%	1.25E-07

V$MTF1_Q4	79	30	38%	2.22E-16
V$PAX3_01	116	44	38%	2E-15
V$ETS2_B	77	29	38%	2.66E-15
V$IRF_Q6	178	67	38%	3.11E-15
V$HIF1_Q3	83	31	37%	6.66E-16
V$IRF_Q6_01	116	43	37%	2E-15
V$XBP1_01	57	21	37%	4.03E-11
V$HTF_01	109	40	37%	2E-15
V$E2F1_Q4	102	37	36%	2.22E-15
V$AR_Q6	14	5	36%	0.001582
V$WHN_B	43	15	35%	5.72E-08
V$VDR_Q3	52	18	35%	3.17E-09
V$LXR_DR4_Q3	96	33	34%	2.22E-16
V$CEBPDELTA_Q6	155	53	34%	1.78E-15
V$CDPCR1_01	79	27	34%	5.7E-13
V$CACCCBINDING	41	14	34%	2.15E-07
V$AHR_01	85	29	34%	8.34E-14
V$COMP1_01	85	29	34%	8.34E-14
V$DEC_Q1	65	22	34%	9.63E-11
V$HNF1_Q6	125	42	34%	1.78E-15
V$PAX3_B	87	29	33%	1.67E-13
V$GCM_Q2	60	20	33%	9.22E-10
V$CEBPA_01	45	15	33%	1.15E-07
V$MYOD_Q6_01	39	13	33%	7.98E-07
V$AP1_Q6	9	3	33%	0.018347
V$NFAT_Q4_01	3	1	33%	0.186963
V$GFI1_01	157	52	33%	2.89E-15
V$RREB1_01	91	30	33%	8.9E-14
V$SRF_Q5_01	68	22	32%	2.61E-10
V$PPAR_DR1_Q2	75	24	32%	5.3E-11
V$ER_Q6_02	25	8	32%	0.00015
V$OCT_Q6	307	98	32%	4.66E-15
V$LXR_Q3	44	14	32%	5.78E-07
V$MEIS1_01	22	7	32%	0.000408
V$GATA1_03	139	44	32%	2E-15
V$HNF1_01	179	56	31%	2.66E-15
V$NFKAPPAB_01	77	24	31%	9.88E-11
V$OCT1_B	276	86	31%	3.33E-15
V$PBX1_01	91	28	31%	4.02E-12
V$AHRARNT_01	98	30	31%	8.1E-13
V$HES1_Q2	49	15	31%	4.05E-07
V$FAC1_01	171	52	30%	2.66E-15
V$HNF1_Q6_01	173	52	30%	2.66E-15
V$NFKAPPAB65_01	97	29	30%	3.75E-12
V$PTF1BETA_Q6	64	19	30%	2.1E-08
V$SMAD4_Q6	27	8	30%	0.000274
V$SRF_Q5_02	129	38	29%	9.99E-16
V$ETS_Q4	17	5	29%	0.004132
V$CP2_01	58	17	29%	1.41E-07
V$CREL_01	106	31	29%	1.32E-12
V$BACH2_01	113	33	29%	2.65E-13
V$ISRE_01	180	52	29%	2.66E-15

V$IRF1_01	149	43	29%	2.22E-15
V$COUP_01	70	20	29%	1.86E-08
V$AP2_Q3	28	8	29%	0.000361
V$GATA1_04	102	29	28%	1.49E-11
V$OCT_C	389	110	28%	6E-15
V$IRF2_01	170	48	28%	2.89E-15
V$STAT5A_01	170	48	28%	2.89E-15
V$ARP1_01	57	16	28%	6.27E-07
V$CIZ_01	43	12	28%	1.67E-05
V$RSRFC4_01	90	25	28%	6.3E-10
V$SRF_C	72	20	28%	3.15E-08
V$MEF2_03	65	18	28%	1.59E-07
V$MYCMAX_02	40	11	28%	4.35E-05
V$NRF2_Q4	124	34	27%	8.68E-13
V$DR1_Q3	66	18	27%	2.05E-07
V$GATA1_01	11	3	27%	0.032606
V$SMAD_Q6	11	3	27%	0.032606
V$COUP_DR1_Q6	92	25	27%	1.05E-09
V$AP1_Q2_01	59	16	27%	1.05E-06
V$PAX1_B	74	20	27%	5.21E-08
V$NFKB_Q6_01	89	24	27%	2.65E-09
V$OCT1_01	127	34	27%	1.83E-12
V$E2F1_Q6_01	45	12	27%	2.77E-05
V$GATA3_01	15	4	27%	0.014866
V$PAX8_B	65	17	26%	8.51E-07
V$AP1_Q4	46	12	26%	3.52E-05
V$HIF1_Q5	58	15	26%	4.31E-06
V$PPARA_01	74	19	26%	2.69E-07
V$PPARG_01	78	20	26%	1.35E-07
V$PPARG_03	78	20	26%	1.35E-07
V$AP1_01	82	21	26%	6.79E-08
V$NRSE_B	119	30	25%	1.79E-10
V$SOX_Q6	128	32	25%	5.73E-11
V$NFE2_01	92	23	25%	2.69E-08
V$VDR_Q6	24	6	25%	0.004149
V$CRX_Q4	173	43	25%	3.8E-14
V$IRF7_01	173	43	25%	3.8E-14
V$TST1_01	133	33	25%	3.61E-11
V$STAT5A_02	129	32	25%	7.13E-11
V$OCT1_Q5_01	97	24	25%	1.69E-08
V$PPARA_02	69	17	25%	2.1E-06
V$CEBPB_02	122	30	25%	3.46E-10
V$HLF_01	139	34	24%	2.81E-11
V$OCT1_Q6	139	34	24%	2.81E-11
V$STAT_Q6	45	11	24%	0.00014
V$STAT5B_01	168	41	24%	2.98E-13
V$TCF11MAFG_01	119	29	24%	8.47E-10
V$NFKB_Q6	78	19	24%	6.52E-07
V$GATA_C	103	25	24%	1.3E-08
V$TGIF_01	66	16	24%	5.21E-06
V$AREB6_03	62	15	24%	1.05E-05
V$CEBPB_01	50	12	24%	8.54E-05

V$CEBP_Q2	50	12	24%	8.54E-05
V$BACH1_01	139	33	24%	1.28E-10
V$S8_01	114	27	24%	6.18E-09
V$HNF3ALPHA_Q6	76	18	24%	1.97E-06
V$P53_01	76	18	24%	1.97E-06
V$SEF1_C	55	13	24%	5.2E-05
V$OCT1_05	195	46	24%	4.04E-14
V$SMAD_Q6_01	34	8	24%	0.001473
V$ICSBP_Q6	282	66	23%	4E-15
V$E2A_Q2	47	11	23%	0.000212
V$MYB_Q6	47	11	23%	0.000212
V$ZID_01	60	14	23%	3.17E-05
V$IK1_01	86	20	23%	7.43E-07
V$LUN1_01	86	20	23%	7.43E-07
V$LMO2COM_02	110	25	23%	5.31E-08
V$LYF1_01	110	25	23%	5.31E-08
V$HSF1_01	66	15	23%	2.34E-05
V$PBX_Q3	81	18	22%	5.21E-06
V$MYOGNF1_01	54	12	22%	0.000188
V$HSF2_01	68	15	22%	3.4E-05
V$SREBP1_02	73	16	22%	2.05E-05
V$MEF2_02	105	23	22%	3.6E-07
V$NFKB_C	105	23	22%	3.6E-07
V$OLF1_01	46	10	22%	0.000763
V$SMAD3_Q6	60	13	22%	0.000135
V$CDP_01	172	37	22%	2.12E-10
V$PR_01	70	15	21%	4.88E-05
V$CEBP_Q2_01	28	6	21%	0.009238
V$E47_02	14	3	21%	0.061993
V$MYOD_01	47	10	21%	0.000912
V$PAX4_04	85	18	21%	1.07E-05
V$ER_Q6	57	12	21%	0.000322
V$TFIIA_Q6	38	8	21%	0.003126
V$HNF4_DR1_Q3	67	14	21%	0.000115
V$E47_01	24	5	21%	0.019238
V$HEN1_02	29	6	21%	0.011003
V$NFKAPPAB50_01	63	13	21%	0.000227
V$MAF_Q6_01	68	14	21%	0.000136
V$SRF_01	112	23	21%	1.2E-06
V$RSRFC4_Q2	127	26	20%	2.67E-07
V$HMX1_01	93	19	20%	1.06E-05
V$MRF2_01	202	41	20%	1.56E-10
V$MEF2_Q6_01	169	34	20%	6.86E-09
V$AMEF2_Q6	150	30	20%	5.86E-08
V$CHOP_01	75	15	20%	0.000112
V$PEBP_Q6	60	12	20%	0.000529
V$ZIC3_01	45	9	20%	0.002558
V$PXR_Q2	40	8	20%	0.004366
V$CEBP_Q3	30	6	20%	0.012994
V$EBF_Q6	30	6	20%	0.012994
V$AP1FJ_Q2	25	5	20%	0.022759
V$HEN1_01	20	4	20%	0.040491

V$CEBP_01	15	3	20%	0.073775
V$NRSF_01	146	29	20%	1.13E-07
V$HMEF2_Q6	121	24	20%	1.37E-06
V$SREBP_Q3	66	13	20%	0.000368
V$SRF_Q6	66	13	20%	0.000368
V$ZIC2_01	112	22	20%	4.38E-06
V$POU3F2_02	293	57	19%	3.18E-13
V$GATA1_05	129	25	19%	1.31E-06
V$AP1_Q2	31	6	19%	0.015223
V$TTF1_Q6	31	6	19%	0.015223
V$FOXP3_Q4	109	21	19%	9.79E-06
V$CEBP_C	99	19	19%	2.69E-05
V$FREAC2_01	302	57	19%	1.18E-12
V$FREAC7_01	122	23	19%	5.54E-06
V$HNF4ALPHA_Q6	80	15	19%	0.000238
V$E12_Q6	16	3	19%	0.086463
V$HOXA4_Q2	59	11	19%	0.001629
V$HMGIY_Q3	102	19	19%	4.15E-05
V$PAX8_01	43	8	19%	0.006894
V$GR_01	97	18	19%	6.87E-05
V$HOXA3_01	124	23	19%	7.35E-06
V$NCX_01	124	23	19%	7.35E-06
V$CHX10_01	178	33	19%	8.91E-08
V$LEF1TCF1_Q4	162	30	19%	3.42E-07
V$TITF1_Q3	81	15	19%	0.000275
V$PAX6_01	130	24	18%	5.12E-06
V$E4BP4_01	223	41	18%	3.45E-09
V$DR4_Q2	98	18	18%	7.9E-05
V$FOXO4_01	44	8	18%	0.007943
V$NFAT_Q6	11	2	18%	0.16398
V$MMEF2_Q6	139	25	18%	5.35E-06
V$AR_03	39	7	18%	0.013604
V$DR3_Q4	39	7	18%	0.013604
V$FOXO1_02	230	41	18%	8.73E-09
V$HNF1_C	135	24	18%	1E-05
V$PITX2_Q2	135	24	18%	1E-05
V$HNF4_01	45	8	18%	0.009107
V$HNF4_01_B	51	9	18%	0.006127
V$HOX13_01	51	9	18%	0.006127
V$SRY_02	57	10	18%	0.00414
V$COREBINDINGF	120	21	18%	4.43E-05
V$FOXO4_02	263	46	17%	2.13E-09
V$IK3_01	103	18	17%	0.000153
V$HAND1E47_01	46	8	17%	0.010394
V$TAL1BETAE47_0	23	4	17%	0.063249
V$AIRE_02	127	22	17%	3.47E-05
V$OCT1_04	81	14	17%	0.000888
V$MSX1_01	128	22	17%	3.93E-05
V$NKX25_01	163	28	17%	3.84E-06
V$FREAC4_01	169	29	17%	2.66E-06
V$ALX4_01	105	18	17%	0.000197
V$FREAC3_01	216	37	17%	1.29E-07

V$PAX6_Q2	82	14	17%	0.001006
V$ROAZ_01	100	17	17%	0.000324
V$LEF1_Q6	136	23	17%	3.46E-05
V$RP58_01	71	12	17%	0.00245
V$PAX_Q6	95	16	17%	0.000532
V$HNF3B_01	101	17	17%	0.000365
V$PBX1_02	90	15	17%	0.000876
V$ERR1_Q2	42	7	17%	0.020067
V$OCT1_03	36	6	17%	0.030368
V$HELIOSA_01	24	4	17%	0.072051
V$GATA2_01	12	2	17%	0.188504
V$NF1_Q6	12	2	17%	0.188504
V$LMO2COM_01	6	1	17%	0.338971
V$BRN2_01	170	28	16%	8.77E-06
V$FXR_Q3	119	19	16%	0.000342
V$GATA1_02	119	19	16%	0.000342
V$EVI1_03	102	16	16%	0.001176
V$PAX4_02	109	17	16%	0.000897
V$CEBPGAMMA_Q	104	16	15%	0.001451
V$AML_Q6	52	8	15%	0.021086
V$FOXO3_01	287	44	15%	2.46E-07
V$HSF_Q6	98	15	15%	0.002116
V$MEIS1BHOXA9_	138	21	15%	0.000338
V$CDPCR3HD_01	40	6	15%	0.047837
V$EVI1_02	40	6	15%	0.047837
V$FXR_IR1_Q6	54	8	15%	0.025944
V$MEIS1AHOXA9_	176	26	15%	0.000118
V$P53_DECAMER_	34	5	15%	0.073019
V$PR_02	48	7	15%	0.038827
V$IPF1_Q4_01	118	17	14%	0.002176
V$CDPCR3_01	112	16	14%	0.003148
V$MYB_Q5_01	14	2	14%	0.238719
V$P53_02	7	1	14%	0.383039
V$ZIC1_01	71	10	14%	0.01907
V$PPARG_02	51	7	14%	0.051469
V$NKX61_01	124	17	14%	0.003686
V$POU6F1_01	219	30	14%	0.000151
V$PIT1_Q6	73	10	14%	0.022774
V$SOX9_B1	112	15	13%	0.007594
V$GATA4_Q3	75	10	13%	0.026971
V$HNF3_Q6	61	8	13%	0.048935
V$TAL1ALPHAE47_	46	6	13%	0.083705
V$EVI1_05	39	5	13%	0.115612
V$BRACH_01	86	11	13%	0.027675
V$FOXD3_01	47	6	13%	0.090815
V$POU1F1_Q6	134	17	13%	0.008048
V$RORA2_01	167	21	13%	0.003898
V$AP4_01	40	5	13%	0.125294
V$AR_01	89	11	12%	0.034519
V$FOX_Q2	138	17	12%	0.01067
V$NKX62_Q2	116	14	12%	0.022574
V$AREB6_01	25	3	12%	0.23081

V$OCT1_02	184	22	12%	0.005837
V$MEF2_01	202	24	12%	0.00447
V$GCNF_01	120	14	12%	0.029228
V$MEF2_04	112	13	12%	0.035986
V$NKX25_02	234	27	12%	0.00404
V$EVI1_04	123	14	11%	0.035086
V$FOXM1_01	80	9	11%	0.084806
V$OCT1_07	198	22	11%	0.013276
V$FOXJ2_02	218	24	11%	0.011194
V$HFH1_01	209	23	11%	0.012858
V$IPF1_Q4	101	11	11%	0.073615
V$TAL1BETAITF2_	83	9	11%	0.101111
V$AIRE_01	47	5	11%	0.202218
V$CDC5_01	179	19	11%	0.030732
V$FOXJ2_01	104	11	11%	0.086554
V$HP1SITEFACTO	156	16	10%	0.057484
V$EVI1_01	117	12	10%	0.090653
V$AFP1_Q6	166	17	10%	0.052076
V$FOXP1_01	118	12	10%	0.095111
V$AR_02	51	5	10%	0.251821
V$HFH3_01	41	4	10%	0.291102
V$GRE_C	104	10	10%	0.15561
V$OCT1_06	73	7	10%	0.21289
V$AR_Q2	105	10	10%	0.162394
V$IK2_01	21	2	10%	0.4129
V$CDP_02	180	17	9%	0.093804
V$CART1_01	266	25	9%	0.053518
V$GR_Q6	32	3	9%	0.360522
V$SOX5_01	75	7	9%	0.232627
V$HNF6_Q6	282	26	9%	0.059969
V$NKX22_01	88	8	9%	0.23248
V$RORA1_01	33	3	9%	0.379071
V$PR_Q2	11	1	9%	0.531829
V$TCF11_01	11	1	9%	0.531829
V$CDX2_Q5	195	17	9%	0.156985
V$NF1_Q6_01	23	2	9%	0.459346
V$HNF3_Q6_01	61	5	8%	0.384566
V$HELIOSA_02	63	5	8%	0.411464
V$LHX3_01	76	6	8%	0.396077
V$CDX_Q5	166	13	8%	0.31497
V$EVI1_06	80	6	8%	0.444285
V$HFH4_01	123	9	7%	0.43627
V$POU3F2_01	128	8	6%	0.625546
V$TEF_Q6	118	7	6%	0.678224
V$NKX3A_01	87	4	5%	0.83944
V$E2F1_Q4_01	1	0	0%	1
V$MAZR_01	1	0	0%	1
