# Supplementary material for: Wide-Scale Analysis of Human Functional Transcription Factor Binding Reveals a Strong Bias towards the Transcription Start Site
Source: PLoS One. 2007 Aug 29;2(8):e807. doi: 10.1371/journal.pone.0000807 (PMC1950076; doi:10.1371/journal.pone.0000807)
Supplement: Text S1 — (0.03 MB DOC) [file pone.0000807.s007.doc]

**Our findings are reproduced using a different method and other databases.**

To check whether our central findings, regarding the significance of the regions near the TSS, depend on the specific method and data used, we turned to validate our results on the basis of single genes that have orthologues in three different organisms (note that Fig 3A was not based on alignment of the BSs in single genes, but rather on overrepresentation on the GO cluster level). We used the UCSC browser [65] (http://genome.ucsc.edu/cgi-bin/hgGateway) which identifies *conserved BS*s – binding sequences with high similarity in human, mouse and rat, that scored above some threshold using the PSSMs obtained from the Transfac Matrix Database (v8.3 created by [Biobase](http://www.gene-regulation.com/pub/databases.html)) [66] and appear in all three organisms at similar locations on the promoters of the orthologous genes. 409 Transfac motifs had at least one conserved binding site in the regions extending to 3000bp upstream from the closest TSS in the human genome. The total number of conserved BSs of these motifs is 39829; of which 11,829 (30%) were found in the first 200bp, compared to approximately 4% found in each of the other 200bp long windows between 400bp to 3000bp upstream (see Figure S3). Checking separately the distribution of BSs of each conserved motif shows that 353 out of the 409 (86%) are significantly over-represented (p <0.05, using the binomial distribution) in the first 200bp-long window (Table S3). Since the PSSM data contain some redundant entries, we repeated the analysis by using a single PSSM from each one of the 274 matrix families. As expected, by using families instead of single PSSM we obtained very similar results

Note that here we introduced an entirely different method of identifying functionally important BSs, using conservation on individual promoters, as given by an independent database (UCSC browser) and even the database used for BS characterization was different (Transfac). Nevertheless, we still find that the most proximal region to the TSS differs significantly from the rest of the promoter; it contains a significantly larger fraction of conserved motifs. This demonstrates the robustness of our findings regarding the special functional role of the proximal region.
